# Targeting Oncogenic Pathways in the Era of Personalized Oncology: A Systemic Analysis Reveals Highly Mutated Signaling Pathways in Cancer Patients and Potential Therapeutic Targets

**DOI:** 10.3390/cancers14030664

**Published:** 2022-01-28

**Authors:** Alexandros Karagiannakos, Maria Adamaki, Antonis Tsintarakis, Borek Vojtesek, Robin Fåhraeus, Vassilis Zoumpourlis, Konstantinos Karakostis

**Affiliations:** 1 Biomedical Applications Unit, Institute of Chemical Biology, National Hellenic Research Foundation (NHRF), 48 Vassileos Constantinou Avenue, 11635 Athens, Greece; alkarg96@gmail.com (A.K.); madamaki@eie.gr (M.A.); Tsintarakis@hotmail.com (A.T.); 2 Research Centre for Applied Molecular Oncology (RECAMO), Masaryk Memorial Cancer Institute, 65653 Brno, Czech Republic; vojtesek@mou.cz (B.V.); robin.fahraeus@inserm.fr (R.F.); 3 Inserm UMRS1131, Institut de Génétique Moléculaire, Université Paris 7, Hôpital St. Louis, F-75010 Paris, France; 4 Department of Medical Biosciences, Umeå University, 90185 Umeå, Sweden; 5 International Centre for Cancer Vaccine Science, University of Gdansk, 80-822 Gdansk, Poland; 6 Institut de Biotecnologia i de Biomedicina, Universitat Autònoma de Barcelona, 08193 Barcelona, Spain

**Keywords:** molecular oncology, precision medicine, tumor, NGS, clinical implementation, cancer patients, mutations

## Abstract

**Simple Summary:**

Currently, genomic databases offer a vast amount of information on mutational profiles and records of statistically significant genetic associations with diseases. However, the functional interpretation of frequently mutated genes implicated in cancer is essential for the development and clinical implementation of efficient targeted therapies. This review identifies and describes the most affected signaling pathways that are found deregulated in several cancer types and reports on corresponding targeted therapies, aiming to unravel literature gaps and to highlight targets of high therapeutic potential. To this purpose, we collected gene lists from the TCGA Pan-Cancer Atlas and OncoKB and employed the Onco Query Language (cBioPortal) search, to identify somatic driver events in 10,439 tumor samples. Each mutation was linked to the affected pathways and to the corresponding tumorigenic potential. Based on this analysis, the 10 most frequently mutated signaling pathways were selected for this review. A detailed description of the mechanistic implications of the identified mutations, as well as the corresponding cancer types and cognate therapeutic applications currently being employed or being under clinical investigation in clinical trials, is discussed. This review aims to explain how the utilization of available genomic mutation data can be employed for the development of targeted therapies, thereby advancing personalized medicine.

**Abstract:**

Cancer is the second leading cause of death globally. One of the main hallmarks in cancer is the functional deregulation of crucial molecular pathways via driver genetic events that lead to abnormal gene expression, giving cells a selective growth advantage. Driver events are defined as mutations, fusions and copy number alterations that are causally implicated in oncogenesis. Molecular analysis on tissues that have originated from a wide range of anatomical areas has shown that mutations in different members of several pathways are implicated in different cancer types. In recent decades, significant efforts have been made to incorporate this knowledge into daily medical practice, providing substantial insight towards clinical diagnosis and personalized therapies. However, since there is still a strong need for more effective drug development, a deep understanding of the involved signaling mechanisms and the interconnections between these pathways is highly anticipated. Here, we perform a systemic analysis on cancer patients included in the Pan-Cancer Atlas project, with the aim to select the ten most highly mutated signaling pathways (p53, RTK-RAS, lipids metabolism, PI-3-Kinase/Akt, ubiquitination, b-catenin/Wnt, Notch, cell cycle, homology directed repair (HDR) and splicing) and to provide a detailed description of each pathway, along with the corresponding therapeutic applications currently being developed or applied. The ultimate scope is to review the current knowledge on highly mutated pathways and to address the attractive perspectives arising from ongoing experimental studies for the clinical implementation of personalized medicine.

## 1. Personalized Therapeutic Strategies Require Understanding of the Underlying Signaling Pathways’ Mechanisms

In 2020, over 19 million people were diagnosed with cancer, with mortality rates reaching approximately 10 million [1]. This makes cancer one of the most critical health concerns of our times and highlights the urgency for devising new and more effective therapies. In this context, it is critical to comprehend the main routes whereby different cancer types arise and progress. Effective treatments are expected to derive from personalized molecular approaches, especially if one considers that cancer usually arises from a variety of causative mutations. A robust impetus was given towards this direction after the completion of the Human Genome Project (HGP) in 2003 [2,3], and several individualized anti-cancer therapies have been developed and applied since then [4,5,6].

Cancer can be regarded as a collection of diseases, all of which share a common feature; the deregulation of key signaling cascades that leads to uncontrolled cellular proliferation [7]. Malignancies arise from alterations in the DNA sequence of genes, as well as from epigenetic changes [8,9,10,11,12]. Both changes induce the activation of oncogenes or the inactivation of tumor-suppressor genes, leading to evasion of growth suppressor function, resistance to cell death (apoptosis), uncontrolled cell cycle, immune evasion as well as increased invasive and metastatic potential [13]. Cancer diagnosis is becoming more reliant on the characterization of mutated pathways that can provide vital information to the separation and clustering of cancer types [14]. Several signaling cascades, including the b-catenin/Wnt, RTK/RAS, p53 and Sonic Hedgehog (SHH) pathways, are linked to cancer formation due to genetic variations that often occur in the respective genes [15,16]. Identification of mutations in genes implicated in such pathways has the potential to reveal the causative driver events of carcinogenesis [17]; it therefore constitutes a topic of paramount importance in precision medicine and for the development of efficient tailor-made drug therapies targeting each cancer type at the molecular level.

Personalized cancer therapy is a treatment strategy that takes into account the molecular profile of patients in order to stratify them into groups that are more likely to benefit from different therapeutic approaches [18,19]. Especially during the past fifteen years, numerous targeted therapies have been approved, extending the survival rates of cancer patients. For example, administration of monoclonal antibodies such as cetuximab, currently used to treat colorectal cancers expressing the *EGFR* gene [20], as well as tyrosine kinase inhibitors such as crizotinib, which is used for the treatment of non-small cell lung cancers (NSCLC) that are positive for *ALK* fusions [21], have been dynamically integrated into oncology practice. In addition to pharmacogenetic approaches, gene editing approaches have also begun to attract a lot of interest and in particular the clustered regularly interspaced short palindrome repeats (CRISPR)/Cas9 system; the latter is currently being tested in multiple clinical trials for its ability to genetically modify immune cells ex vivo (e.g., PD-1 knockout T-cells), and to enhance the anti-cancer immune response when these cells are infused back into the patient [22,23]. Functional studies and clinical implementation of CRISPR/Cas9 are highly anticipated for the development of more effective anti-cancer therapies that target specific genetic alterations, and are thereby capable of restoring their function.

## 2. Systemic Analysis Determines Prevalent Mutated Carcinogenic Pathways

The aim of this review is to highlight the importance of the underlying mechanisms in crucial carcinogenic signaling pathways. To this purpose, we focus on the signaling pathways that are most frequently found deregulated in cancers. We have specifically focused on cancer-related pathways previously identified by Pan-Cancer Atlas project studies [14,24,25,26,27,28] and have calculated the frequencies of each driver mutation to the corresponding pathways. For our analysis we used publicly available data on 10,439 tumor samples from 32 TCGA Pan-Cancer Atlas studies [29]. Our results suggest that the ten main pathways known to be implicated in cancer development can be sorted in descending order based on their driver mutation frequency, as follows: p53, RTK-RAS, lipids metabolism, PI-3-Kinase/Akt, ubiquitination, b-catenin/Wnt, Notch, cell cycle, homology directed repair (HDR) and splicing.

**Figure 1:**
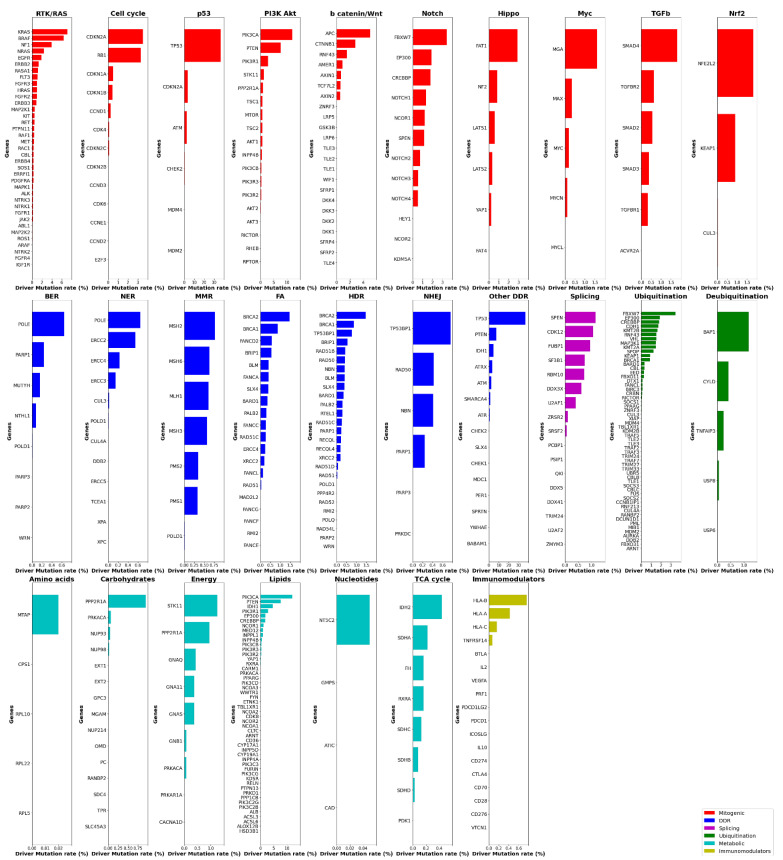
Somatic driver mutational frequency of 204 cancer genes involved in 27 signaling pathways implicated in six major cellular procedures. For the calculations, mutational data from 10,439 tumor samples were examined. Percentages refer to the total number of samples examined (10,439).

**Figure 2:**
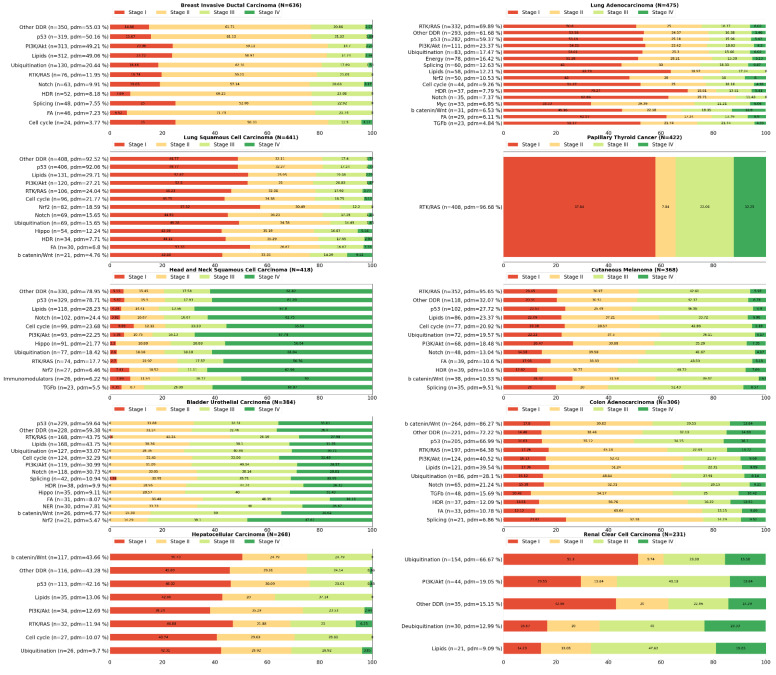

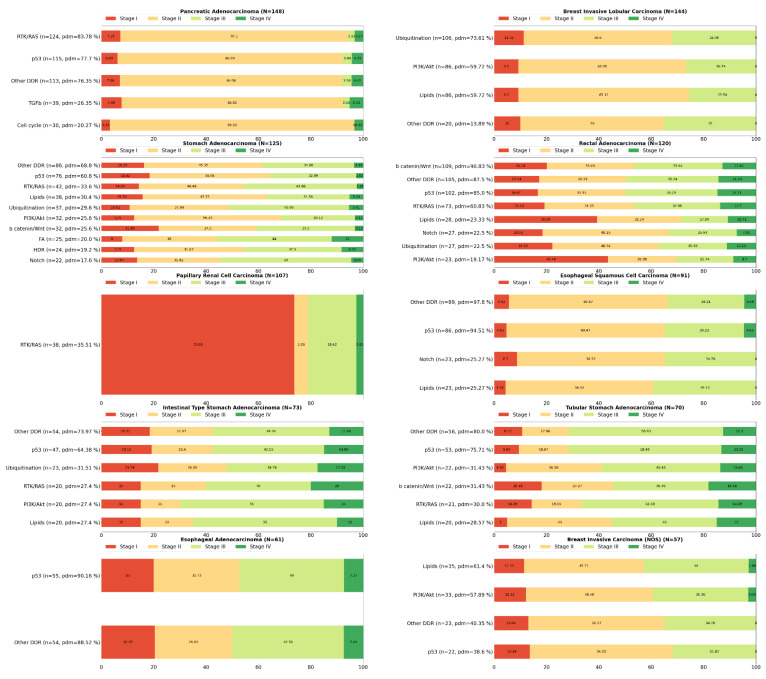

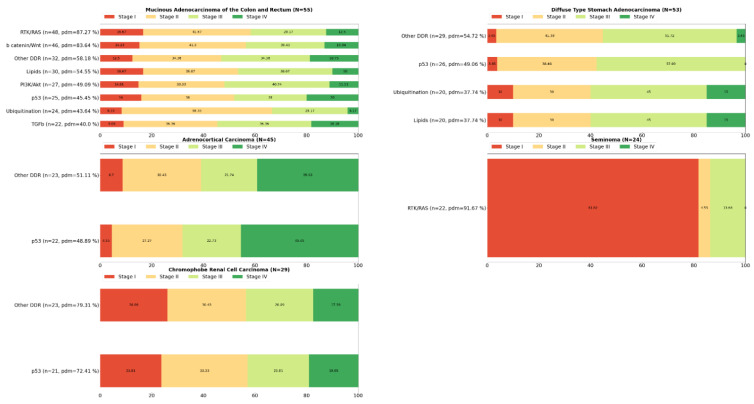
Crucial signaling pathways implicated in 25 cancer types/subtypes per disease stage. For this analysis, 5283 tumor samples with an available mutational profile and disease stage were examined. Here, only signaling pathways altered in at least 20 of the examined samples for each cancer type/subtype are shown. N: number of samples examined; n: number of samples that harbor somatic driver mutations in each signaling pathway; pdm: proportion of samples of a particular cancer type/subtype that harbor at least one somatic driver mutation in genes of a particular signaling pathway.

Gene lists were created by collecting data on signaling pathways from the TCGA Pan-Cancer Atlas publications (3004 genes in total, available in https://www.cell.com/pb-assets/consortium/pancanceratlas/pancani3/index.html (accessed on 1 November 2021)). Then, we filtered only those genes that belong to the OncoKB cancer gene list (340 genes), (version update: February 2021). Using the Onco Query Language (OQL) provided by cBioPortal, we searched specifically for somatic driver mutations in these genes in 10,439 mutationally profiled tumor samples (10,066 primary, 364 metastatic and 9 recurrent) included in 32 TCGA Pan-Cancer Atlas studies. We ascertained that more than 95% of the samples affected by driver events in the genes of interest (7915 out of 8276) belonged to the “primary origin” category, and therefore became the focus of subsequent analyses. Data were extracted from the cBioPortal for Cancer Genomics open-access resource. Somatic driver mutations were identified in 204 of the 340 cancer genes in this particular dataset, so their corresponding pathways were marked for further analyses. The selection of the driver events was made in line with the definition of cBioPortal (https://www.cbioportal.org/oql#driver (accessed on 1 November 2021)) and OncoPrint (default: OncoKB and CancerHotspots). As such, mutations, fusions and copy number alterations constitute ‘drivers’. Note that germline and somatic modifiers only apply to mutations and that very few studies on the public cBioPortal contain germline data. The types of alterations found in the 7915 samples are the following: frame_shift deletions or insertions, fusions, in frame deletions or insertions, missense mutations, nonsense mutations, nonstop mutations, slice regions or sites and translation start sites. Data management, calculations and graph construction were performed using Spyder IDE, a scientific Python development environment. Numerous TCGA Pan-Cancer Atlas efforts have been made to elucidate the genomic background of cancer, each focusing on the deregulation of a different physiological cellular procedure [14]. Each one of these procedures involves a number of signaling pathways that contain genes with a distinct tumorigenic potential (Figure 1). Furthermore, our mutational data analysis has shown that most cancers are characterized by deregulation of a group of pathways, rather than a single signaling pathway, but in a distinct pattern from other cancer types, while in some tumor types, a subtype-related differential representation of altered pathways or even a stage-dependent pathway perturbation also seems to exist (Figure 2). The Python code, as well as the typed query for driver events in the genes of interest using the cBioPortal Onco Query Language (OQL) can be found in the supplementary information (Supplementary Materials S1 and S2).

This review therefore focuses on the ten most frequently deregulated signaling pathways and as such only the results pertaining to these pathways are discussed below. In the next sections, we describe how specific genetic alterations/mutations in encoding genes of a given pathway may lead to carcinogenesis and/or cancer progression. For each pathway, a brief description is provided regarding its implication in cancer development, along with a description of the most highly mutated genes, and their percentile frequencies, as calculated in our analysis. In addition, we list some related targeted therapies that have been approved by the Food and Drug Administration (FDA) and are currently being integrated into clinical practice, as well as some emerging therapies which are currently being tested in clinical trials.

## 3. The p53 Pathway

**Figure 3:**
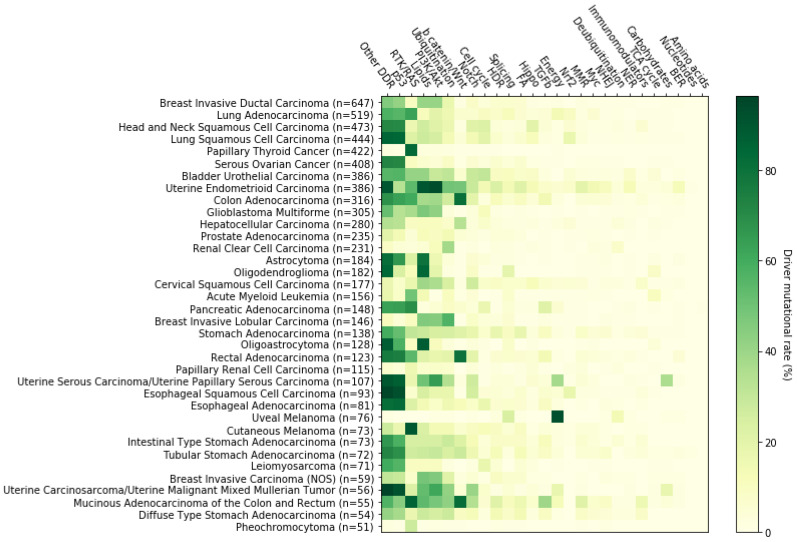
Alteration rate of 27 signaling pathways across 36 cancer types/subtypes. For this analysis, 10,066 tumor samples of primary origin and with available mutational profiles were examined for the presence of somatic driver mutations in our 204 genes of interest. Calculations were performed by taking into account all the available mutationally profiled primary tumor samples of the cBioPortal selected studies (10,066 samples) instead of the driver event-harboring mutationally profiled primary tumor samples (7915 samples). If for a selected cancer type there were x mutationally profiled primary tumor samples available in the cBioPortal selected studies, then, y samples would bear at least one driver event in at least one of the 204 genes of interest and z samples would harbor driver events in genes participating in a selected signaling pathway. The corresponding driver mutational rate for this cancer type-signaling pathway pair is calculated as (z/x) × 100. Here, only cancer types/subtypes that entail more than 50 analyzed samples are shown. From left to right, signaling pathways are displayed in descending order of total mutational frequency. n: number of samples examined.

Components of the p53 pathway function cooperatively to ensure that the cell will respond effectively against a variety of stress signals that threaten cellular homeostasis and genomic integrity. In this process, p53 target genes trigger several pathways linked to cancer, such as cell cycle arrest, senescence or apoptosis, blood vessel formation and the regulation of metabolism [30,31,32,33,34]. The importance of *TP53* in cancer development is evident by the fact that it occupies the first place in the ranking of genes found to be most frequently altered in cancer [35,36]. Indeed, the number of cancer types/subtypes where p53 pathway is likely not perturbed is very small (e.g., uveal melanoma) (Figure 3). In the majority of cancer types, more than 30% of the samples examined harbored driver mutations in at least one key gene of this pathway, while in some cases this percentage exceeded 90% (e.g., esophageal squamous cell carcinoma). Even though there is a high number of cancers that bear a variant in one or more components of the p53 pathway, the affected components are specific to three genes in this pathway. We found that among those patients, 93.66% had a driver mutation in *TP53*, whereas *CDKN2A* and *ATM* genes were affected in 7.71% and 6.27% respectively, dictating the simultaneous presence of some of these mutations in a number of cases. *TP53* driver mutations can be located in virtually any region of the gene, thus affecting the ability of the p53 protein to appropriately interact with either its protein effectors or the DNA [37]. According to our analysis, arginine residues on positions 273, 248 and 175, all of which belong to the DNA-binding domain [38], are the most frequently mutated, representing 7.06%, 5.87% and 4.41% of patients that have an affected *TP53* gene, respectively. R273 variants have been associated with an increased proliferative and invasive potential, which in the case of R273H, are induced by the inhibition of tumor suppressor *KLF6* [39,40]. Similar effects seem to take place when a R248 mutant is present, but this particular mutation leads to a robust binding of certain variants—mainly the mutp53^R248Q^—to the STAT3 protein, and to the subsequent activation of the latter [41,42]. The R175H variant, on the other hand, is associated with a radical change in p53 conformation which triggers the transactivation of a panel of genes and results in elevated c-met protein levels and to the induction of tumor invasion, among other events [43,44].

In addition to missense mutations and other mutation types, the *CDKN2A* gene is frequently affected by nonsense mutations, in line with our analysis, which demonstrates that R80*, R58*, W110*, E120*, Y44* and E88* together constitute almost a third of cancer cases that harbor a defective *CDKN2A* gene. Such alterations shut down one of the cell cycle’s checkpoints, as they force the p16^INK4a^ protein to lose its Cdk-binding ability, thereby being unable to prevent the G1/S transition [45,46,47]. From the remaining two thirds of cases, an aberrant splicing involving the D153 residue appears in 5.16% of all *CDKN2A* affected cancer patients. Changes in this position may inactivate both *CDKN2A* gene products, i.e., p16^INK4a^ and p14^ARF^, leading to loss of cell cycle control via both pRB and p53 inactivation [46].

Factors upstream of p53 are also affected and, along with the ATM kinase, play a vital role in the activation of p53 in response to DNA damage [48,49,50,51,52,53,54]. In our dataset, non-sense mutations leading to premature truncated forms of the ATM protein product represented more than one third of all *ATM* mutated cases, with R250* being the main representative. In these cases, the final gene product loses its functionality as a main DNA damage sensor, either partially or completely, thus increasing the vulnerability of the cell to malignant transformation [55]. Our analysis has also revealed that R337C/H are the most common cancer-related missense mutations in the *ATM* gene, accounting together for 7.94% of all *ATM* variant carriers. Although these mutations have been documented in cancer patients [56,57], to the best of our knowledge their functional impact has not been elucidated yet [58].

As expected, the dynamic presence of these genes on the mutational cancer map, has made the p53 pathway—and especially the *TP53* component—an attractive therapeutic target. Among a plethora of p53-targeting strategies, the most promising fall into one of the following categories: (i) restoring the function of p53 protein or (ii) impeding the interaction between p53 and its main negative regulator, the MDM2 E3 ubiquitin ligase [35,59,60]. In this context, eprenetapopt (APR-246), a mutant-p53 conformation resetting agent, is probably the most promising compound [61] and the only therapy of this category that is currently being tested in a phase III clinical trial (NCT03745716); on the other hand, a phase III trial of idasanutlin (NCT02545283), another emerging MDM2 inhibitor, was recently terminated due to low efficacy. The results of such clinical trials, as well as the efforts to improve the properties of the relevant compounds are highly anticipated. Apart from p53, there are four FDA approved anticancer drugs targeting *CDKN2A* signaling: abemaciclib, palbociclib, ribociclib and trilaciclib [62,63,64,65]. All these therapeutic agents act as CDK4/6 inhibitors, aiming to restore the inactivated *CDKN2A* [66,67]. However, it has been noted that p16^ΙΝΚ4a^ loss does not necessarily predict response to CDK4/6 inhibitors [68,69,70,71,72]. The rationale behind *ATM* deficient-related therapeutic approaches however, is to cause synthetic lethality. There is evidence that this is feasible by further weakening DNA repair mechanisms via administration of appropriate inhibitors, mainly PARP and ATR inhibitors, either alone or in combination [73,74,75]. Ongoing clinical trials will validate the potential of preliminary preclinical trial results and their translation into clinical practice.

## 4. The RTK-RAS Pathway

RTK-RAS is probably the most thoroughly studied cancer-related signaling pathway. Its involvement in a multitude of crucial physiological processes, such as cell growth, proliferation, differentiation, angiogenesis, integrin signaling and cell migration [76,77,78], among many others, makes it clear that deregulation of this pathway can facilitate both tumor initiation and tumor progression. Indeed, our analysis showed that in 12 of 36 cancer types and subtypes examined, more than 30% of patients carried driver mutations in RTK-RAS pathway genes, with this percentage rising above 80% in papillary thyroid cancer, cutaneous melanoma and mucinous adenocarcinoma of the colon and rectum. On the other hand, as with the p53 pathway, no uveal melanoma patient was found to be affected (Figure 3). The predominantly mutated genes involved in this pathway, i.e., *KRAS*, *BRAF* and *NF1*, were found mutated in 26.05%, 17.06% and 13.06% of RTK-RAS perturbed cases respectively, while additional mutations in all cancer-related receptor tyrosine kinase-encoding genes represented 33.2% of all mutations. In descending order of mutational frequency, these were as follows: *EGFR*, *ERBB2*, *FGFR3*, *FLT3*, *FGFR2*, *ERBB3*, *RET*, *KIT*, *MET*, *ERBB4*, *PDGFRA*, *ALK*, *FGFR1*, *NTRK3*, *NTRK1*, *ROS1*, *NTRK2*, *IGF1R* and *FGFR4*.

Ιn our cohort, we ascertained that *KRAS* is almost exclusively mutated (missense mutations) on the G12, G13, Q61 and A146 residues, corresponding to more than 95% of all cases (75.41%, 11.68%, 5.84% and 3.67%, respectively). Mutants of codon 12, 13 and 61 decrease the GTP-hydrolysis rate, with mutants of codon 12 and 16 also being capable of increasing the rate of GDP-exchange [79,80], while mutants of codon 146 act mainly through the second mechanism [81,82]. Both mechanisms lead to increased levels of the activated GTP-bound form of the KRAS protein, resulting in uncontrolled mitogenic processes via a constitutively active signal transduction [80,83].

V600 is the most prevalent mutated codon of the *BRAF* gene, occurring in 81.74% of all *BRAF* mutated primary tumors in our dataset, while rearrangement of *BRAF* with a variety of genes seemed to occur in only 4.36% of these samples. In the majority of cases where a V600 variant exists, the valine residue is substituted by glutamic acid (V600E), enhancing the serine/threonine kinase activity of BRAF, thus leading to the abrogation of extracellular signals, promoting cell growth and proliferation [84,85,86].

In contrast to *BRAF*, there is no hotspot position for the *NF1* gene. The most common somatic mutation of *NF1*, namely R2450*, barely exceeds 3% of all identified variants in our cohort. In addition, our analysis showed that nonsense mutations, frameshift deletions and splice-site variants are the most frequent mutation types for this gene in cancer, accounting for 39.91%, 20.83% and 15.79% of all identified variants, respectively. Regardless of the mutation type, in the majority of *NF1*-affected cases, mutations lead to a loss of neurofibromin GTPase-activating function, resulting in sustainably high GTP-bound levels of RAS proteins and thereby in a continuous tumor-promoting signaling process via RTK-RAS and PI3K/AKT pathway hyperactivation [87,88,89,90].

Regarding the development of targeted anti-cancer therapies, RTK-RAS is the most extensively utilized signaling pathway. Indicatively, 64 targeted therapies have already been approved by the FDA, targeting 18 out of the 37 genes of this pathway (Table 1) [91]. For many years the *KRAS* gene was considered untargetable [92,93,94,95]. A milestone study in 2021, leading to the discovery of the first KRAS-targeted therapy using sotorasib, an eclectic KRAS^G12C^ inhibitor, was approved for adult NSCLC patients carrying this variant who had previously been treated with at least one systemic therapy [96]. Another selective KRAS^G12C^ inhibitor, adagrasib, owing to its tolerability and clinical effectiveness, was granted a Breakthrough Therapy Designation by the FDA and its approval is expected soon [97]. With several more clinical trials underway e.g., JDQ-443/NCT04699188, mesenchymal stromal cells-derived exosomes with KRAS G12D siRNA/NCT03608631 and anti-KRAS G12V mTCR PBL/NCT03190941), a new era seems to be emerging for cancer treatment. Furthermore, over the last ten years, three selective BRAF^V600E^ inhibitors have been approved by the FDA: vemurafenib, dabrafenib and encorafenib [98,99,100]. These therapeutic agents, especially in combination with MEK inhibitors cobimetinib, trametinib and binimetinib respectively, have provided substantial progress-free survival (PFS) and overall survival (OS) benefit to melanoma, non-small cell lung cancer and thyroid cancer patients harboring BRAF^V600E^. Notably, dabrafenib and encorafenib target both *BRAF* and *RAF1*, both of which belong to the RAF family, but constitute different genes. Therefore and in line with the “Personalized Cancer Therapy” knowledge base for precision oncology (https://pct.mdanderson.org/#/home/BRAF?section=Drugs (accessed on 19 January 2022)), these genes have not been colored in blue in Table 1.

## 5. Lipid Metabolism

Both lipid synthesis and catabolism are essential for the maintenance of membrane functionality, protein trafficking, immune cell responses, signaling, as well as coverage of metabolic demands, such as energy production and storage [101,102,103,104,105]. It has been shown that cancer cells manage to retain a high proliferative potential, through various metabolic modifications [13,106,107]. According to our analysis, more than 40% of patients of one in three cancer types/subtypes are characterized by driver mutations in genes involved in lipid metabolism. In uterine endometrioid carcinoma, astrocytoma, oligodendroglioma and oligoastrocytoma this proportion reached or exceeded 80%, while, as with the two pathways discussed above, no primary uveal melanoma tumor of our dataset was affected (Figure 3). At the gene level, *PIK3CA*, *PTEN* and *IDH1* occupy the first places in the mutagenicity ranking, as they were found altered in 45.21%, 27.56% and 16.93% of the patients, respectively. Over 95% of *PIK3CA* somatic alterations in cancer are missense mutations, half of which include three substitutions: E545K, H1047R and E542K. These substitutions exert a gain-of-function effect on the PI3K protein via two different mechanisms. Amino acid (aa) residues E545 and E542 are located in the helical domain of p110alpha protein and their substitution by a lysine residue attenuates the inhibitory interaction between the catalytic subunit (p110alpha) and the nSH2 domain of the regulatory subunit (p85alpha) of the PI3K protein, promoting the continuous activation of PI3K [108,109]. A constitutive PI3K activation is also the result of the kinase domain variant H1047R, but in this case, p110alpha abolishes its C-terminal tail self-inhibitory capacity [110]. Both mechanisms lead to a strong activation of PI3K downstream effectors, such as the AKT and P70S6K proteins, which mediate protein synthesis, cell growth, cell proliferation, angiogenesis, survival, and thus contribute to tumorigenic transformation [111,112,113,114]. At the same time, hyper-activated PI3K/AKT signaling induces the expression of genes involved in fatty acid anabolism, a process that generates the essential building blocks for the synthesis of new cells [115,116].

*PTEN* alterations show greater variety than *PIK3CA* alterations, with missense mutations, frameshift insertion-deletions (indels) and nonsense mutations representing 91% of all carriers. The most prevalent mutations involve R130, R233 and T319 aa residues and are found in 17.5%, 5% and 4.74% of all affected patients, respectively. In the majority of cases, arginine in position 130 is either substituted by a glutamine or a glycine residue, or, as in the cases of R233 and T319, the codon that is responsible for encoding it, is converted to a stop codon. Whereas truncating mutations such as R130*, R233* and T319* lead to an unstable, non-functional protein product [117,118,119], R130Q and R130G variants generate stable proteins, which, however, lack phosphatase activity [118,120]. Absence or non-functionality of the PTEN protein prevents PIP3 dephosphorylation, which in turn accumulates and recruits PI3K signaling effectors, such as AKT and PDK1 [121]. Furthermore, it was recently shown that PTEN^R130Q^ mutants tend to accumulate at the cell periphery where they form leading edges that increase tumor invasiveness and further activate the PI3K/AKT signaling axis [122].

A very impressive paradigm of a predominant mutational hotspot is represented by the *IDH1* gene. Of the 467 somatic driver mutations that we identified in an equal number of cancer patients, 466 carried an R132 replacement, with histidine being the most frequent substitute (388/466 cases); cysteine, glycine, serine and leucine substitutions occurred in 50, 16, 11 and one patients, respectively. These variants, which are mapped in the catalytic pocket of the enzyme, make isocitrate dehydrogenase-1 convert the normal final product of its catalytic activity, alpha-ketoglutarate (aKG), into R(−)-2-hydroxyglutarate (2HG), with concomitant NADPH consumption [123,124]. The following 2HG-mediated inhibition of aKG-dependent deoxygenases, such as TET2 and JMJD2A/C, promotes global gene expression changes, which along with the redox stress arising from the declined NADPH levels, reflect the tumorigenic impact of these mutations [124,125,126,127]. Likewise, even though the decreased levels of two lipogenesis components, NADPH and aKG [128] would be expected to abrogate lipid synthesis, certain lipid precursors, such as glycerol-phosphates and glycerophosphocholine, are present in elevated quantities; on the other hand, other lipid precursors, as for example myo-inositol phosphate, are present in reduced levels, compared to unaffected tumors, suggesting that cancer cells harboring *IDH1* variants, alter their phospholipid expression profile, probably in a tumor-assisting manner [129,130,131].

As made clear from the above, signaling changes involving lipid metabolism regulators perturb a wide spectrum of cellular processes and contribute to cancer development. This observation has led to the development of therapies that mitigate these changes. Currently, there are eleven clinically available therapies that target the signaling of four out of the 24 lipid metabolism-related genes found mutated in our dataset (Table 2) [91]. As our analysis suggests, when lipid metabolism is deregulated on account of a genetic change, this alteration invariably affects one of its aforementioned PI3K/AKT signaling-mediating regulators. Hence, inhibition of the PI3K/AKT signaling axis is suggested to be the main goal of personalized medicine for the treatment of tumors bearing such alterations. When PI3K catalytic subunit inhibitors, both isoform-specific, such as alpelisib, and pan-isoform, such as copanlisib and duvelisib, as well as selective MTOR inhibitors, including everolimus and temsirolimus, are employed, they seem to offer a significant PFS—or even OS—benefit to patients with a variety of blood or solid malignancies [132,133,134,135,136]. In addition, ivosidenib, an inhibitor of *IDH1*^R132^ mutants, has been approved by the FDA, for the treatment of acute myeloid leukemia (AML) patients harboring these variants [137], while its effectiveness in other *IDH1*-deficient cancer types is currently being tested in several ongoing clinical trials (e.g., advanced and metastatic cholangiocarcinoma/NCT02989857, chondrosarcoma/NCT04278781, cholangiocarcinoma—chondrosarcoma—glioma—other advanced solid tumors/NCT02073994, recurrent ependymoma—recurrent ewing sarcoma—recurrent hepatoblastoma/NCT04195555, etc.).

## 6. The PI3K/AKT Pathway

The impact of the PI3K/AKT signaling network on diverse cellular functions is well established. Many of these, including cell growth and proliferation, survival, motility, cellular metabolism, immune system functions and angiogenesis, are tightly intertwined with cancer development and progression and as a result a lot of research has been dedicated to unraveling the deregulation mechanisms of this pathway in cancer [138,139,140,141]. In 26 of the 36 cancer types of our dataset, more than 10% of patients carried at least one driver mutation in genes involved in this pathway. Uterine malignancies showed the highest mutational rates with 93.3% of uterine endometrioid carcinoma patients being affected, while the corresponding proportion of the remaining uterine tumors analyzed exceeded 60%. On the other hand, less than 5% of serous ovarian cancer, papillary thyroid cancer, pheochromocytoma, uveal melanoma and acute myeloid leukemia patients appeared to bear such changes (Figure 3). Among all PI3K/AKT-deregulated tumors examined, 53.75% harbored driver mutations in *PIK3CA*, 32.76% in *PTEN* and 12.93% in *PIK3R1*, with the rest of the genes in this pathway being altered in less than 6% of the tumors each. Interestingly, simultaneous mutations in at least two, or in certain cases in all of the above mentioned top mutated genes, were present in 16.34% of these patients. As driver mutations and therapeutic applications regarding *PIK3CA* and *PTEN* genes were discussed earlier in this report, we will now focus on the *PIK3R1* gene. Somatic mutations in *PIK3R1* exhibit a highly scattered pattern. The absence of predominant hotspots is reflected by the mutational rates of the most prevalent genetic changes of this gene; 6.67% for R348*, 5.67% for X582_splice and 4% for G376R. R348* is a truncating but gain-of-function mutation that exerts its tumorigenic impact in both a PI3K/AKT-dependent and a PI3K/AKT-independent manner. In addition to activating PI3K/AKT signaling, p85alpha mutants are localized into the nucleus and promote the activation of ERK and JNK kinases, thereby inhibiting FASL-mediated apoptosis and inducing cell survival, growth, proliferation and invasion [142,143]. Even though X582 splice alteration has been previously identified and considered as pathogenic [144], its exact functional consequences have not been elucidated yet. Finally, the nSH2 domain-located G376R substitution acts in the same way as the previously discussed E545 and E542 substitutions of the p110alpha protein, thus attenuating the inhibitory interaction between the regulatory and the catalytic subunit of the PI3K complex and enhancing the PI3K/AKT signaling [145,146].

Overall, the PI3K/AKT signaling-promoting behavior of p85alpha mutants, places *PIK3R1*-targeting in the same therapeutic context as the other major effectors of this pathway, *PIK3CA* and *PTEN*. As shown in Table 2, the available agents targeting the signaling of these genes are essentially the same. In addition to PI3K and MTOR inhibitors, significant efforts are being made to develop AKT inhibitors [147,148]. Most of the ongoing clinical trial test agents exert a pan-AKT (AKT1, AKT2 and AKT3) inhibition. Of those, capivasertib and ipatasertib have demonstrated the most encouraging results and are now tested in several phase III trials mainly regarding breast and prostate cancer patients [149] (NCT03997123/NCT04493853/NCT04862663/NCT04305496 and NCT03072238/NCT03337724/NCT04060862/NCT04650581/NCT04177108, respectively).

## 7. Ubiquitination and Acetylation Pathways

Ubiquitination is a reversible modification that leads either to protein degradation or to the regulation of protein–protein interactions. It is essential for the appropriate execution of various cellular events, namely inflammation, translation, endocytosis, DNA damage response (DDR), protein trafficking, differentiation and signal transduction [150,151,152,153,154]. Thus, deregulation of the ubiquitin pathway can lead to cancer initiation and/or progression. Mutations in genes encoding components or regulators of the ubiquitination machinery are not uncommon in cancer samples. Our analysis demonstrates that such mutations are present in more than 10% of patients, in two out of three cancer types (24/36). The highest rates are shown in uterine carcinosarcoma, mucinous adenocarcinoma of the colon and rectum, uterine endometrioid carcinoma and breast invasive lobular carcinoma, ranging from ~42% to ~57%. Contrariwise, oligodendroglioma, papillary thyroid cancer, leiomyosarcoma, uveal melanoma and pheochromocytoma, exhibited the lowest rates with less than 3% of the patients being affected (Figure 3). At the gene level, the most frequently mutated genes, *FBXW7* (or *FBW7*), *EP300* and *CREBBP* (or *CBP*) were detected in 19.6%, 10.72% and 9.88%, respectively, of ubiquitination/deubiquitination-deficient tumors, while the mutational rate of nine more genes ranged between ~5% and ~9%. In descending order of mutational frequency, these are as follows: *KMT2B*, *RNF43*, *VHL*, *MAP3K1*, *KMT2A*, *SPOP*, *BAP1*, *KEAP1* and *BRCA1*.

Three arginine residues of the FBW7 ubiquitin ligase—R465, R505 and R479—represent 43.59% of all FBW7 somatic driver mutations in primary tumor samples. These positions are located into the WD40 domain, which constitutes the substrate-binding site of the SCF complex (a type of E3 ligase), which is responsible for the ubiquitin-labeling and the subsequent proteasome-mediated degradation of its protein effectors [155,156]. These mutations induce changes in the WD40 domain, altering the interaction potential of FBW7, probably via both hydrophobic and electrostatic interactions, as well as by limiting the contact surface because of the shorter substitute sidechains (mainly cysteine, histidine, glycine and glutamine) [157]. Given that several FBW7 interactors act as regulators of cell growth, apoptosis and proliferation [158,159,160,161,162,163]; prevention of their degradation may result in tumorigenesis.

Even though the *EP300* gene is usually altered by nonsense mutations, the most recurrent mutations fall into three other categories. The D1399N substitution is the most prevalent somatic mutation of this gene, accounting for 7.29% of *EP300*-mutated primary tumors in our working dataset. Missense mutations in this position were shown to change the conformation of the p300 protein histone acetyltransferase (HAT) domain, leading to abolishment of its autoacetylation activity, which is essential for appropriate function [164,165]. The subsequent inability of p300 to stimulate other tumor suppressors in the nucleus, such as RB1, BRCA1, p53 and AP-2alpha [166,167,168,169,170], paves the way for the predominance of its spatial distinct, cytoplasmic ubiquitin ligase activity, which targets p53 for degradation [171,172,173]. These changes, together with the reduced global levels of histone H3 acetylation [174], contribute to the tumorigenic impact of this genetic alteration. The second and third most prevalent mutations of the *EP300* gene are the frameshift deletion M1470Cfs*26 and the splice site variant X1429 splice, and were each identified in 2.6% of *EP300*-affected patients. However, their functional impact is not clear. Another gene with intrinsic histone acetyltransferase activity, sharing high similarity to *EP300*, is the *CREBBP* gene. Missense mutations involving the R1446 are the most recurrent somatic mutations of *CREBBP*, followed by frameshift indels involving I1084 and substitutions of D1435, accounting for 7.91%, 5.65% and 2.82% of *CREBBP*-affected primary tumors, respectively. Both R1446 and D1435 are located in the HAT domain, which is responsible for the catalytic activity of the CBP transcriptional coactivator. Substitution of these aa residues, reduces the acetyl-CoA binding affinity of CBP, thereby impairing its acetyltransferase activity [175,176,177]. Consequently, CBP can neither activate p53 tumor suppressor nor inactivate BCL6 proto-oncoprotein. Furthermore, as with its structurally and functionally related p300 protein, CBP also exhibits an E4 ubiquitin ligase activity targeting p53 for degradation in the cytoplasm [171]. These observations, in conjunction with the arisen extended transcriptional changes, dictate a tumor promoting effect for these mutations [175,178]. The functional impact of frameshift deletion I1084Sfs*15, which is found in 4.52% of *CREBBP*-altered samples thus far remains obscure.

Proteins involved in either the attachment or the removal of ubiquitin moieties are barely exploited in clinical practice nowadays [179]. An exception to this rule is provided by thalidomide analogues lenalidomide and pomalidomide, which have been approved for the treatment of various blood malignancies and exhibit anti-tumor activities by altering the specificity of cereblon, which constitutes the substrate recognition component of the CRL4 E3 ubiquitin ligase complex [180,181]. Nonetheless, targeting of the proteolytic machinery is primarily oriented to proteasome inhibition, with three inhibitors—bortezomib, carfilzomib and ixazomib—already approved for the treatment of multiple myeloma and mantle cell lymphoma [182], while other agents, such as delanzomib (CEP-18770) and marizomib or salinosporamide A (NPI-0052) [183] are currently being tested in clinical trials as potential treatment options for solid tumors (NCT00572637 and NCT03345095, respectively).

## 8. The WNT/b Catenin Pathway

The WNT/b catenin pathway is one of the best-studied signaling cascades in cancer development, known to be implicated in several cancer-related cellular functions, such as cell proliferation, stem cell maintenance, differentiation, cell–cell adhesion, morphogenetic processes, migration, angiogenesis and immune evasion [184,185,186,187,188]. Mutations in WNT/b catenin components are very common in cancer patients. Our analysis revealed that carcinogenic mutational occurrences in genes of this pathway are limited compared to the pathways discussed so far. Specifically, the proportion of patients with at least one driver mutation in this pathway exceeded 10% in only nine of the thirty-six cancer types examined here. Intestinal malignancies ranked highest in terms of mutational landscape with at least 80% of colon adenocarcinoma, rectal adenocarcinoma and mucinous adenocarcinoma of the colon and rectum patients harboring driver alterations. On the other hand, no such mutations were found in uveal melanoma or leiomyosarcoma patients (Figure 3). Among WNT/b catenin effectors, the *APC*, *CTNNB1* and *RNF43* genes were found mutated in 48.59%, 26.41% and 14.94% of the affected samples, respectively, while four other genes (in descending order of mutational frequency: *AMER1*, *AXIN1*, *TCF7L2* and *AXIN2*) exhibited a driver mutational rate between ~5% and ~8%.

The most frequent mutations are nonsense mutations in the *APC* tumor suppressor, present in 40.6% of patients. Conversion of arginine-encoding codons into stop codons predominantly takes place at positions 1450, 876 and 1114 of the APC protein (8.51%, 4.84% and 4.64% respectively). R1450 is located into the so-called mutation cluster region (MCR) [189]. Truncation of the APC protein in this position leads to loss of all axin- and most b catenin-binding sites, therefore abrogating the ability of APC to negatively regulate b catenin via the formation of the destruction complex AXIN-APC-CK1alpha-GSK3beta [190,191,192,193]. Similarly, two other truncations, namely the R1114* and R876* mutants, also lose the axin- and most b catenin-binding sites [194,195]. In all three cases, the subsequent b catenin accumulation and nuclear entry permits activation of the TCF/LEF1 transcriptional complex, thereby promoting cellular proliferation and tumorigenesis [192].

Somatic mutations in the *CTNNB1* (catenin-beta 1) gene, which is involved in the regulation of cell adhesion and gene transcription, are almost exclusively missense mutations. In particular, aa substitutions at six specific positions (32, 33, 34, 37, 41 and 45) represent more than 85% of all affected patients in our dataset. Among them, S33, S37 and D32 replacements were the most prevalent, affecting 18.51%, 17.44% and 16.01% of all cases, respectively. The protein region between D32 and S45 participates in the phosphorylation of b catenin from CK1alpha and GSK3beta (components of its destruction complex), as well as in the interaction of phosphorylated b catenin with its E3 ligase substrate recognition component, FBW1 [196,197]. Consequently, mutations in these protein sites exert similar signaling implications to the ones exerted by APC, as b catenin escapes proteasomal degradation and confers an increased proliferative potential to the cell [197,198,199,200,201].

Another gene found mutated in cancer patients is the *RNF43* (Ring Finger Protein 43), a downstream target gene of Wnt/b catenin signaling. Almost seventy-three percent of *RNF43*-mutated primary tumors carry a frameshift deletion in this gene, while the second most frequent alteration type is a non-sense mutation (~17%). By far the most common alteration of the RNF43 protein is G659Vfs*41, being present in approximately 60% of all *RNF43*-mutated tumors. Interestingly, despite its recurrent presence in cancer samples, this frameshift deletion leaves protein function intact, with the relevant RNF43-mutant being able to exert its E3 ubiquitin ligase activity [202]; this results in the tagging of FZD family (frizzled transmembrane proteins) WNT receptors for proteasomal degradation and in the inactivation of WNT/b catenin signaling [203,204]. The second and third most frequent somatic mutations of the RNF43 protein are R519* and R145*, affecting only 2.52% and 1.89% of all carriers, respectively. Both of these aberrations lead to a truncated protein with a WNT signaling-enhancing role, however the exact mechanism differs depending on the truncation position. The catalytic RING domain of RNF43 lies among P270 and I316 [202,205], and as such the R519* mutant retains its E3 ubiquitin ligase activity. Nonetheless, it simultaneously gains the ability to snare CK1alpha at the plasma membrane, thus assisting b catenin to escape degradation [206]. On the other hand, R145* variants lack this catalytic activity and are therefore expected to abort their FZD degradation and b catenin destabilization role [202].

Significant efforts have been made to therapeutically target the WNT/b catenin network, mostly for the development of small molecule stabilizers of the b catenin destruction complex components or destabilizers of the b catenin-TCF/LEF interaction, but also for the development of antibodies and regulatory peptides that directly or indirectly affect—mostly inhibit—WNT or FZD proteins [192,207,208]. Four such constructs have exhibited the most encouraging results and their efficacy is currently evaluated in phase II clinical trials of both solid and blood cancer patients. In this context, WNT974 (NCT02278133)—a porcupine inhibitor that impedes WNT secretion and activity, Foxy-5 (NCT03883802)—a WNT5a mimetic, PRI-724 (NCT01606579)—an antagonist of the b catenin coactivator CBP, and DKN-01 (NCT03395080)—a monoclonal antibody that neutralizes the activity of WNT/b catenin axis inhibitor DKK1, are now in the spotlight of WNT pathway targeting.

## 9. The Notch Pathway

Notch is a cancer-related signaling pathway well-known for its involvement in a variety of developmental processes, as well as cellular differentiation, proliferation, stem cell maintenance, angiogenesis, EMT, inflammation and apoptosis [209,210,211]. Mutations in the Notch pathway, were found in at least one in ten patients in fifteen out of thirty-six assessed cancer types. Uterine carcinosarcoma exhibited the highest driver mutational rate (40.35%), followed by mucinous adenocarcinoma of the colon and rectum (33.93%) and bladder urothelial carcinoma (29.02%), while other gynecological and upper digestive tract malignancies, were also highly affected. On the other hand, none of the patients with pheochromocytoma and no more than 2% of patients with leiomyosarcoma, uveal melanoma and papillary thyroid cancer carried such mutations (Figure 3). At the gene level, the most frequently mutated components or regulators of the Notch pathway appeared to be identical to the above discussed ubiquitination pathways; *FBXW7*, *EP300* and *CREBBP* were found altered in 33.65%, 18.41% and 16.97% of Notch-impaired tumors, respectively, while somatic driver events in each of the *SPEN*, *NCOR1* and *NOTCH1* genes, were detected in ~12% of cases. Upon activation of the NOTCH receptors by their ligands (e.g., delta-like protein 1, protein jagged-1 etc), the NOTCH-intracellular domain (NICD) is released and transferred into the nucleus where it acts as a transcription regulator. NICD is one of the FBW7 substrates [159] and mutations within the FBW7 WD40 domain have been reported to impede this interplay, therefore leading to NICD accumulation and enhanced Notch signaling, which may lead to a tumorigenic outcome [157,212,213]. Furthermore, despite the previously reported p300 requirement for NICD transcriptional activity [214,215], it was recently demonstrated that loss-of-function mutations in either the *EP300* or *CREBBP* gene can also activate the Notch axis, due to the subsequent low histone acetylation levels of the *FBXW7* promoter [216], suggesting the implication of additional transcriptional NICD co-activators.

So far, no targeted therapies for Notch signaling regulation have entered the clinical practice. However, remarkable efforts have been made to overcome the obstacles associated with Notch pathway signaling, given the highly context-specific behavior of this signaling axis in cancer [217]. To this end, diverse strategies have been utilized, such as the targeting of NOTCH biosynthesis enzymes, receptor-ligand interplay, NOTCH cleavage-performing effectors or NICD-containing transcriptional complexes assemblage [217,218,219]. The most encouraging results come from the development of inhibitors against gamma-secretase—which is responsible for the final NICD-releasing NOTCH cleavage—and delta-like protein 3, a NOTCH ligand, with some of the relevant cancer-related clinical trials being in advanced stages. Specifically, the efficacy of gamma-secretase inhibitors (GSIs) nirogacestat/PF-03084014 (NCT03785964) and MK0752 (NCT00756717) is currently evaluated in phase III trials in adults with desmoid tumor and in early stage breast cancer patients in combination with tamoxifen respectively, while the tesirine conjugated anti-delta-like protein 3 mAb rovalpituzumab (Rova-T) (NCT03061812) is being tested in a phase III clinical trial in small-cell lung cancer (SCLC) patients with disease progression following platinum-based chemotherapy and overexpressing delta-like protein 3.

## 10. The Cell Cycle Pathway

The cell cycle is a set of fine-tuned, strictly inspected processes which mediate between the end of two consecutive cell divisions and are responsible for the apt preparation of the cell towards a complete, equal and accurate cell material distribution between daughter cells. Consequently, functional disruption of key cell cycle mediators can provoke loss of proliferation control, as well as a disturbance of the genomic integrity, both fundamental features of cancer [220,221]. Driver somatic mutations of crucial cell cycle components are present in more than 10% of patients in 25% of the cancer types studied. For some malignancies the occurrence rate exceeds even 20%, with bladder urothelial carcinoma, head and neck squamous cell carcinoma and lung squamous cell carcinoma patients being affected in 30.24%, 22.52% and 20.04% of cases, respectively. At the same time, no such alterations were found in patients with renal clear cell carcinoma, acute myeloid leukemia, uveal melanoma or pheochromocytoma (Figure 3).

Indisputably, the retinoblastoma tumor suppressor *RB1* and the previously discussed *CDKN2A* are the most commonly mutated cell cycle genes, identified in 44.43% and 41.11% of all cell cycle-perturbed primary tumors, respectively; despite ranking third in mutational frequency, the *CDKN1A* gene was found mutated in 6.76% of assessed tumors. Somatic mutations were highly dispersed along the *RB1* gene, with 203 of the 928 aa-encoding codons found to be implicated in a driver event in our dataset. Even though more than 40% of these genomic changes were non-sense mutations, the most recurrent appears to be the splice site mutation X405_splice, which nevertheless accounts for only ~3% of *RB1*-mutated tumors. Among the nonsense alterations, R320* and R552* were the most prevalent, being present in 2.09% of cases each. All three of these alterations constitute inactivating mutations that impair both RB1 functional domains (A- and B-boxes) [222,223,224,225]. Such alterations abrogate one of the cell cycle restriction points, as a non-functional or absent RB1 protein permits E2F-mediated G1/S transition, thereby enhancing the cell’s proliferative potential and contributing to tumorigenesis or tumor progression [226,227]. On the other hand, somatic driver genomic alterations in *CDKN1A* are extremely rare, as only 51 out of 10,066 (~0.5%) assessed primary tumor samples appeared to be affected, with the majority detected in bladder urothelial carcinoma (39/51) and hepatocellular carcinoma (7/51) patients. Frameshift indels and nonsense mutations represent the lion’s share of *CDKN1A* gene alterations. Q10* and M38Nfs*10 are the most recurrent among them, accounting for 7.84% and 5.88% of p21-deficient tumors, respectively. Although experimentally uncharacterized, both mutations are expected to abrogate p21 functionality due to loss of most functional domains. Such aberrations are likely to assist tumor progression, as deficient p21 is unable to prevent cell cycle progression and DNA synthesis through cyclin-CDK complexes or PCNA inhibition [228,229,230,231,232], while in the case of M38Nfs*10 frameshift insertion, the intact 33 NH2-terminal aa residues, where procaspase-3 binding domain is located, might further facilitate tumor progression due to sustenance of p21 anti-apoptotic activity [233,234].

Thus far, therapeutic targeting of the cell-cycle is largely based on CDK4/6 inhibition [235]. Three such inhibitors, palbociclib, ribociclib and abemaciclib have already received FDA approval either as monotherapy or in combination with hormone therapies, for the treatment of advanced HR^+^/HER2^-^ breast cancer [62,63,64]. Recently, another CDK4/6 inhibitor, trilaciclib, was also approved for the prevention of chemotherapy-induced myelosuppression in small cell lung cancer (SCLC) patients [65]. In parallel, many more such inhibitors are currently being tested in clinical trials for their potential to confer improved anti-tumor activity, but accompanied by less toxicity. Of these, dalpiciclib (SHR6390) is the only compound currently tested in phase III trials (NCT03966898 and NCT03927456), while many pan-CDK inhibitors are being evaluated in earlier phases of clinical development [91]. Apart from CDKs, several inhibitors of other cell-cycle components, such as CHK1, PLKs and Aurora proteins, have also entered clinical testing [236].

## 11. The HDR Pathway

Homology-directed DNA repair (HDR) is one of the two major mechanisms responsible for double strand breaks (DSBs) repair [237,238]. Given the detrimental impact of DSBs on genomic stability [239], malfunction of the HDR pathway may facilitate malignant transformation [240]. Our analysis showed that somatic driver mutations in HDR pathway components are not rare events in cancer. In particular, 23.71% of patients with uterine endometrioid carcinoma harbored such mutations, while the relative fractions in five gastrointestinal cancer types—mucinous adenocarcinoma of the colon and rectum, stomach adenocarcinoma, diffuse type stomach adenocarcinoma, tubular stomach adenocarcinoma and colon adenocarcinoma—were 21.43%, 17.39%, 13.89%, 12.66% and 10.95%, respectively. In contrast, less than 1% of patients with papillary thyroid cancer and oligodendroglioma, and none of the oligoastrocytoma patients carried such alterations (Figure 3). Among HDR compounds, *BRCA2*, *BRCA1* and *TP53BP1* were the most frequently affected genes, being altered in 25.17%, 14.73% and 13.01% of HDR-impaired patients, respectively; the relative mutational rates of eight additional genes (in descending order of mutational frequency: *BRIP1*, *RAD51B*, *NBN*, *RAD50*, *BLM*, *SLX4*, *BARD1* and *PALB2*), ranged between ~5 and ~9%.

Almost 41% of somatic mutations in the *BRCA2* tumor suppressor are truncating mutations, most of which result in loss of multiple BRCA2 functional domains. However, the most recurrent BRCA2 protein changes are the frameshift insertion N1784Kfs*3, the frameshift deletion K1691Nfs*15 and the missense mutation R2842C, which were detected in 3.4%, 2.72% and 2.04% of all *BRCA2*-mutated primary tumor samples in our analysis, respectively. The R2842 substitution by a cysteine residue has been demonstrated to mitigate HDR efficiency [241]. Although N1784Kfs*3 and K1691Nfs*15 have been previously identified [242], their exact functional consequences remain unresolved. Nevertheless, the final shortened variants lack crucial BRCA2 C-terminal domains, such as some of the RAD51-binding sites, the DNA-binding domain and their nuclear localization sequences (NLSs) [238,243], likely dictating a loss-of-function effect. A dysfunctional BRCA2 protein is incapable of efficiently recruiting RAD51 at DSB sites, thereby preventing RAD51 nucleoprotein filament formation. This hinders homology-directed invasion of damaged DNA to the intact sister chromatid [244,245,246], thereby contributing to genome instability, one of the hallmarks in cancer. Similar to *BRCA2*, nonsense mutations are also predominant in the *BRCA1* gene (~41%). At the protein level, the most common mutations appear to be the frameshift insertion E111Gfs*3 and the nonsense mutations E720* and E572*, accounting for 3.49% and 2.33% of all BRCA1-affected tumors, respectively. Despite the previous identification of all three variants [247], their functional consequences have not been experimentally validated yet. However, the truncation sites of these mutants dictate the loss of coiled-coil domain, RAD50- and RAD51-binding domains and the two BRCT domains, while E572* and E111Gfs*3 variants are additionally expected to lack one or both BRCA1 nuclear localization sequences, respectively [248,249,250,251]. Such variants are most likely unable both to prevent 53BP1-mediated inhibition of the initial HDR step, viz., DNA end resection by MRE11-RAD50-NBS1 (MRN) complex [252], and to recruit and stabilize the RAD51 protein onto the DSB sites [251], thus resulting in HDR deficiency. Nonsense mutations and frameshift deletions account for ~80% of *TP53BP1* driver somatic mutations in cancer, mainly corresponding to E737* (6.58%), N1017Mfs*20 (6.58%) and E711Nfs*12 (3.95%). All three constitute truncating mutations leading to loss of both the NLS and the four C-terminal functional domains of 53BP1 protein. It was recently demonstrated that such changes result in significant retardation of DSB repair, suggesting impairment of the 53BP1-mediated non-homologous end joining (NHEJ) repair mechanism [253]. Although 53BP1 can act as an HDR negative regulator [252,254], the predominant role of NHEJ on DSB repair, primarily during the G1 phase, when the HDR is inactive [255], renders 53BP1 deficiency a genome stability-threatening situation.

Targeted therapy of HDR-deficient tumors is highly based on the synthetic lethality concept [256,257,258], wherein mutations in different genes can lead to cell death when they appear concurrently. In this context, BRCA1/2-deficient tumors, which lack the ability to accurately repair DSBs, can be treated with single strand break (SSB) repair inhibitors, such as inhibitors of the base excision repair-mediators PARP1/2 [259]. Four such agents, olaparib, rucaparib, niraparib and talazoparib, are clinically available for the treatment of pretreated advanced breast, ovarian and fallopian tube or primary peritoneal cancer patients harboring BRCA1/2 mutations (this restriction does not apply to niraparib), either germline or/and somatic [260,261,262,263,264]. Several more PARPis are in clinical development with three of them, fluzoparib (NCT03863860), pamiparib (NCT03519230) and veliparib (NCT02470585, NCT02163694 and NCT02152982), currently being tested in phase III trials. In addition to the BRCA1/2m—PARPis couple, increasing evidence demonstrates that synthetic lethality may also take place following ATR inhibition in HDR-deficient tumors carrying inactivated ATM [265,266,267], with active ongoing phase II clinical trials testing two such agents: berzosertib (NCT02567409, NCT03517969 and NCT02595892) and ceralasertib (NCT03330847, NCT03328273, NCT03787680 and NCT02937818), in a variety of human cancers.

## 12. The Splicing Pathway

Splicing is the process of exon joining following intron exclusion that takes place during the conversion of pre-mRNA to its mature translatable form. Under certain circumstances, an aberrant splicing procedure can be oncogenic [268,269,270,271]. Our analysis revealed that somatic driver events involving splicing machinery components are not uncommon in malignancies. Thus, cancers from different anatomical regions, namely uveal melanoma, oligodendroglioma, uterine endometrioid carcinoma, lung adenocarcinoma, mucinous adenocarcinoma of the colon and rectum, and bladder urothelial carcinoma, exhibited a splicing pathway-related mutation rate of 23.75%, 18.52%, 13.92%, 10.95%, 10.71% and 10.24%, respectively. In contrast, less than 1% of patients with glioblastoma multiforme and pheochromocytoma, and none of the patients with papillary thyroid cancer or leiomyosarcoma harbored such mutations (Figure 3). At the gene level, *SPEN*, *CDK12* and *FUBP1* appeared to be the most commonly mutated genes, being identified in 20.82%, 19.89% and 17.84% of splicing-impaired tumors, respectively, whereas the corresponding proportion of four additional genes fluctuated between ~8% and ~14% (in descending order of mutational frequency: *RBM10*, *SF3B1*, *DDX3X* and *U2AF1*).

The *SPEN* gene, which encodes for the SMRT/HDAC1-associated repressor protein (SHARP or SPEN), is mostly affected by nonsense mutations and frameshift deletions (~81% of driver events). The most recurrent protein alterations include I1052Sfs*40, A2251Qfs*102 and P2495Lfs*4 (2.68% each), however none of these has so far been functionally characterized. Nonetheless, the undetectable SPEN protein levels accompanying an insertion/truncation in position 1184 [272], point towards a loss-of-function in the I1052Sfs*40 variant. Furthermore, all three variants lack the C-terminal SPOC domain [205], which is essential for SPEN transcriptional co-repressor activity, as it mediates the interaction with SMRT/NCoR [273]. SPEN-deficient cells are not capable of hindering ER alpha oncogenic effects, thereby contributing to tumor formation [272]. However, the mechanism via which abolishment of SPEN splicing-related activity—likely a link between RNA splicing and mRNA nuclear export machines [274]—may contribute to tumorigenesis, remains unclarified. Another highly mutated gene involved in splicing is the cyclin-dependent kinase 12 (*CDK12).* Nearly 80% of patients affected by a somatic driver mutation in *CDK12* gene, harbor a fusion, a frameshift deletion or a nonsense mutation. Nevertheless, the R890H substitution is the most recurrent variation (3.74%), followed by the frameshift deletion P683Qfs*70 and the CDK12-IKZF3 fusion (2.8% each). The functional consequences of these alterations have not been experimentally delineated yet. R890 is located into the kinase domain (aa residues 737–1020) [275]. Proximal aa substitutions have been shown to either prevent CDK12 interaction with its protein partner cyclin K, or to considerably decrease its kinase activity [276]. In addition, P683Qfs*70 truncation results in the elimination of almost its entire kinase domain. The inability of CDK12 to phosphorylate the C-terminal domain of RNA polymerase II, prevents it from transcriptionally-activating several target genes of the HDR pathway, as well as of other DDR components [276,277,278]. Furthermore, CDK12-deficiency may promote proximal alternative last exon (ALE) splicing of certain DDR genes, such as *ATM*, thus limiting the abundance of full-length protein products [279], and in this way impeding DNA repair processes and further contributing to genome instability and cancer development.

The *FUBP1* gene is another highly mutated gene involved in splicing (far upstream element-binding protein 1, FBP), which regulates MYC expression by binding to a single-stranded far-upstream element (FUSE). The most frequent *FUBP1* mutations are frameshift deletions, detected in 37.5% of all carriers. The frameshift deletion S11Lfs*43 is the most frequently repeated somatic alteration, being present in 9.38% of patients, followed by R430C substitution (8.33%)—the only missense mutation identified in 96 FBP-mutated patients—and frameshift indels involving I301 (5.20%). All these changes have been identified by massive sequencing approaches, but are not yet characterized [280,281]. Variants harboring either S11Lfs*43 or I301-involving out-of-frame mutations, namely deletions I301Yfs*22 and I301Nfs*22 as well as insertion I301Nfs*4, lose more than 90% or at least half of their protein body respectively, which suggests a loss-of-function effect. Although known as a positive regulator of the MYC proto-oncogene [282], it is the previously reported FBP participation in a MYC-repressing complex with FIR [283] that is consistent with a tumor-promoting scenario. Additionally, it has been demonstrated that FBP loss hampers proper MDM2 splicing, giving rise to the tumorigenesis-accelerating *MDM2*-*ALT1* splice variant [284].

Therapeutic targeting of the splicing pathway is still far from entering the clinic. However, strategies have been developed towards this direction. These include three main approaches: (i) spliceosome assembly disruption through the inhibition of core spliceosome components (e.g., SF3B1) or their regulators (e.g., SRPKs and CLKs) using small molecules, (ii) control of the utilized splicing regulatory elements (exonic and intronic splicing enhancers or silencers) or inhibition of cancer-related aberrantly expressed splicing factors by oligonucleotides, and (iii) targeting of abnormal splice isoforms [285,286,287]. Currently, the first strategy has come to the fore, being the only approach to include compounds under clinical evaluation. Thus, two SF3B-complex (a component of U2 snRNP) inhibitors, E7107 (NCT00459823 and NCT00499499) and H3B-8800 (NCT02841540) are currently being tested in phase I trials for their safety and anti-tumor activity, against solid and hematological malignancies, respectively.

## 13. Conclusive Remarks

In this study, we review the most popular cancer-related signaling pathways as sorted by their somatic driver mutational rates calculated in our analyses. Several pathways can be simultaneously deregulated in a tumor. Our analysis showed that this applies to the majority of tumors. This may result either from putatively independent co-alterations of two or more genes implicated in discrete signaling pathways, or due to alterations in genes acting as nodes between multiple signaling cascades (e.g., the *PIK3CA* and *PTEN* genes in PI3K/AKT and lipid metabolism pathways). The complexity of the underlying interlinked mechanisms makes it difficult to identify a single disease-causative implicated pathway. From a conceptual point of view, genes may play distinct roles in more than one pathway and a driver mutation can be implicated in the dysregulation of several pathways. As such, the designation of one particular pathway (or a part of the associated pathways) as the ‘most highly associated to a tumor’, is supported by the mutational profiles (the combination of mutations occurring in the genes involved in each pathway). Analysis of the ten pathways with the highest mutational frequencies led to the identification of the cancer types most frequently affected, which in turn facilitated the description of the most repeatedly identified alterations/mutations in key members of these pathways and their direct or indirect consequences in the signaling process; importantly, the potential for therapeutic exploitation of these alterations is discussed, which may further highlight new targets for personalized cancer treatment opportunities.

Despite the complexity that the simultaneous deregulation of multiple signaling cascades in many tumor types creates, the present study manages to unravel, at least to a certain extent, the remarkable diversity of mechanisms by which somatic mutations can drive cell signaling perturbance in cancer. For example, in our analysis, somatic driver mutations in key regulators of RTK-RAS, lipid metabolism and WNT/b catenin pathways, appeared to co-exist in nearly 40% of patients with mucinous adenocarcinoma of the colon and rectum. Furthermore, mutations in different regions of a single gene may deregulate distinct signal transduction networks. Such a paradigm is represented by the R348* mutation in PIK3R1 which, in contrast to other protein region alterations that seem to suffer only PI3K/AKT axis activation as a functional consequence, it appears to additionally enhance MAPK signaling, making cells sensitive to MEK and JNK inhibition [142]. These observations underline the importance of patient mutational profiling for therapeutic decision-making and provide valuable insight to the functional and mechanistic characterization of cancer-related variants.

Several recurrent cancer-related variants remain uncharacterized. These involve components of crucial signaling pathways, including PI3K/AKT, ubiquitination, cell cycle and splicing. *CREBBP* and *FUBP1* genes represent some of the most interesting cases. The cellular compartment-dependent roles of CBP either as a histone acetyltransferase or as an E4 ubiquitin ligase and their opposing effects on p53 activation [171], highlight the importance of investigating I1084-related frameshift effects, due to their potential therapeutic value. S11Lfs*43, R430C and the three identified I301-involving frameshift indels, together account for more than one fifth of *FUBP1*-affected tumors. The recent inclusion of the *FUBP1* gene in the “long-tail driver” category of the less-frequently mutated genes due to its alternative splicing regulatory role in several oncogenes and tumor suppressors [288], makes it a very attractive therapeutic target. New horizons in cancer treatment may also arise from more adequately understood cancer contributors. Thus far, targeting of the ubiquitin-proteasome system (UPS) is primarily based on proteasome inhibition. However, broad use of this strategy is limited due to problems caused by the accumulated ubiquitin-labeled proteins [179]. Therefore, targeting earlier steps of the process might surmount this hurdle. Furthermore, the tumor-suppressor *FBXW7*, which is mutated in one fifth of ubiquitination-deficient tumors, also appears to be an appealing target, given its role in the destabilization of many proto-oncoproteins [289]. Considering the well-studied and clinically exploitable synthetic lethality concept, exploration of new DDR member couples, apart from BRCA1/2-PARPs, may also prove valuable. For example, *TP53BP1* is one of the most frequently mutated members of the major DNA DSB repair pathways (HDR and NHEJ), and driver mutations in this gene are mutually exclusive to *BRCA1*/*2* mutations in 80% of cases, thereby providing a template for extending the synthetic lethality concept to HDR-proficient tumors. The already established synthetic lethal interaction between 53BP1 and DNA polymerase theta, the key mediator of another DSB repair mechanism called theta mediated end joining (TMEJ) [290,291], is in line with this notion.

Despite focusing on just one of the –omics areas, this work gives a useful overview of the current knowledge on signaling pathway deregulation in cancer. Understanding the impact of such molecular events in cell signaling could provide the basis for better exploitation of the existing targeted therapies, and also facilitate the enrichment of the current therapeutic arsenal with agents targeting frequently affected but still considered as undruggable pathways, such as the splicing pathway, thereby paving the road to more effective combinatorial therapies.

## Figures and Tables

**Table 1:** FDA approved drugs targeting the signaling of 18 cancer-related genes of the RTK-RAS pathway. Drugs that specifically target only the corresponding gene are shown in blue color. Drugs granted with a Breakthrough Therapy Designation but not yet approved by the FDA, are not included in this table.

**Gene Signaling**	**Drugs**
*ABL1*	Bosutinib, Brigatinib, Dasatinib, Ibrutinib, Imatinib, Niotinib, Pazopanib, Ponatinib, Regorafenib, Sunitinib, Tivozanib, Vandetanib
*ALK*	Alectinib, Brigatinib, Ceritinib, Crizotinib, Entrectinib, Gilteritinib, Lorlatinib, Sunitinib
*BRAF*	Binimetinib, Cobimetinib, Dabrafenib, Dasatinib, Encorafenib, Regorafenib, Sorafenib, Trametinib, Vemurafenib
*EGFR*	Afatinib, Brigatinib, Ceritinib, Cetuximab, Dacomitinib, Erlotinib, Gefitinib, Ibrutinib, Lapatinib, Lorlatinib, Mobocertinib, Necitumumab, Neratinib, Osimertinib, Panitumumab, Vandetanib.
*ERBB2*	Afatinib, Dacomitinib, Everolimus, Gefitinib, Ibrutinib, Lapatinib, Margetuximab, Metformin, Mobocertinib, Nelfinavir, Neratinib, Pertuzumab, Sirolimus, Temsirolimus, Trastuzumab, Trastuzumab Deruxtecan, Trastuzumab Emtansine, Tucatinib
*FGFR1*	Brigatinib, Dasatinib, Erdafitinib, Infigratinib, Lenvatinib, Nintedanib, Pazopanib, Ponatinib, Regorafenib, Sorafenib, Sunitinib, Tivozanib, Vandetanib
*FGFR2*	Brigatinib, Ceritinib, Erdafitinib, Infigratinib, Lenvatinib, Nintedanib, Pazopanib, Regorafenib, Sorafenib, Sunitinib, Vandetanib
*FLT3*	Brigatinib, Cabozatinib, Ceritinib, Fedratinib, Gilteritinib, Ibrutinib, Midostaurin, Nintedanib, Pexidartinib, Sorafenib, Sunitinib, Vandetanib
*KIT*	Axitinib, Cabozatinib, Dasatinib, Fedratinib, Imatinib, Infigratinib, Lenvatinib, Midostaurin, Nilotinib, Pazopanib, Pexidartinib, Ponatinib, Regorafenib, Sorafenib, Sunitinib, Tivozanib
*KRAS*	Binimetinib, Cobimetinib, Sotorasib, Trametinib
*MET*	Cabozatinib, Capmatinib, Crizotinib, Tepotinib, Tivozanib
*NRAS*	Binimetinib, Cobimetinib, Trametinib
*NTRK1*	Cabozatinib, Crizotinib, Entrectinib, Larotrectinib, Lorlatinib, Regorafenib, Sorafenib, Sunitinib
*NTRK2*	Cabozatinib, Entrectinib, Larotrectinib, Lorlatinib, Sorafenib, Sunitinib
*PDGFRA*	Axitinib, Dasatinib, Ibrutinib, Imatinib, Lenvatinib, Midostaurin, Nilotinib, Nintedanib, Olaratumab, Pazopanib, Ponatinib, Regorafenib, Sorafenib, Sunitinib, Tivozanib
*PTPN11*	Binimetinib, Cobimetinib, Trametinib
*RET*	Alectinib, Brigatinib, Cabozatinib, Ceritinib, Fedratinib, Ibrutinib, Lenvatinib, Pazopanib, Ponatinib, Pralsetinib, Regorafenib, Selpercatinib, Sorafenib, Sunitinib, Vandetanib
*ROS1*	Brigatinib, Cabozatinib, Ceritinib, Crizotinib, Entrectinib, Lorlatinib

**Table 2:** FDA approved drugs targeting the signaling of four cancer-related genes of the lipid metabolism pathway. Drugs that specifically target only the corresponding gene are shown in blue color.

Gene Signaling	Drugs
*IDH1*	Ivositenib
*PIK3CA*	Alpelisib, Copanlisib, Duvelisib, Everolimus, Metformin, Midostaurin, Sirolimus, Temsirolimus
*PIK3R1*	Alpelisib, Copanlisib, Duvelisib, Everolimus, Idelalisib, Midostaurin, Sirolimus, Temsirolimus, Umbralisib
*PTEN*	Alpelisib, Copanlisib, Duvelisib, Everolimus, Metformin, Midostaurin, Sirolimus, Temsirolimus

## Supplementary Materials

The following are available online at https://www.mdpi.com/article/10.3390/cancers14030664/s1, Supplementary Material S1: Code of the analysis, Supplementary Material S2: Query for searching for driver events of the genes of interest using cBioPortal Onco Query Language (OQL).
