# Supplementary material for: Targeting Oncogenic Pathways in the Era of Personalized Oncology: A Systemic Analysis Reveals Highly Mutated Signaling Pathways in Cancer Patients and Potential Therapeutic Targets
Source: Cancers (Basel). 2022 Jan 28;14(3):664. doi: 10.3390/cancers14030664 (PMC8833388; doi:10.3390/cancers14030664)
Supplement: Supplementary file 1 [file cancers-14-00664-s001.zip › Supplementary Material S1 (code).pdf]

Program that isolates only the Literature reviewed genes from a matrix containing both Literature reviewed and Computationally predicted genes involved in ubiquitylation process.

```
import pandas as pd

cd ~\python programming\2

ubq = pd.read_csv("ubq.csv")

# In ubq.csv there are Literature reviewd, as well as Computationally predicted
# genes implicated in ubiquitylation process. We want to take into account only
# the Literature reviwed ones.

ubq = ubq[ubq.Validation_status == "Literature reviewed"]

ubq_genes = list(ubq.Gene)
```

Program that creates a table containing driver cancer genes from 30 cancer related pathways, the pathway they belong to and the percent of tumor samples (out of 10439 samples from 32 cancer types of PanCancerAtlas projects) where these genes are found mutated.

```
cd ~\python programming\2

import pandas as pd

genes = pd.read_csv("gene_mutation_frequencies.csv")

i = -1
for freq in genes.Frequency:
   i += 1
   genes.Frequency[i] = float(freq.replace(",", "."))

# Mitogenic pathways

RTK_RAS = "MAP2K2	MAPK1	MAP2K1	RAF1	BRAF	ARAF	NRAS	HRAS
	KRAS	RAC1	RASA1	NF1	ERRFI1	PTPN11	CBL	SOS1	JAK2
	FLT3	ROS1	ALK	RET	ABL1	NTRK3	NTRK2	NTRK1	PDGFRA
	KIT	MET	FGFR4	FGFR3	FGFR2	FGFR1	ERBB4
	ERBB3	ERBB2	IGF1R	EGFR".split()
cell_cycle = "RB1	CDK2	CDK6	CDK4	CCNE1	CCND1	CCND2	CCND3
	CDKN2A	CDKN2B	CDKN2C	CDKN1A	CDKN1B	E2F1
	E2F3".split()
p53 = "CDKN2A	TP53	MDM2	MDM4	ATM	CHEK2
	RPS6KA3".split()
PI3K_Akt = "PTEN	PIK3R2	PIK3R1	PIK3R3	PIK3CA	PIK3CB
	INPP4B	AKT1	AKT2	AKT3	PPP2R1A	STK11	TSC1	TSC2	RHEB	RICTOR
	MTOR	RPTOR".split()
b_catenin_Wnt = "WIF1	SFRP1	SFRP2	SFRP3	SFRP4	SFRP5	DKK1	DKK2	DKK3	DKK4
	LRP5	LRP6	RNF43	ZNRF3	AXIN1	AXIN2	AMER1	CTNNB1	GSK3B
```

```
	APC	TCF7	TCF7L1	TCF7L2	TLE1	TLE2	TLE3	TLE4".split()
Notch = "JAG2	ARRDC1	FBXW7	CUL1	NOV	CNTN6	NOTCH1
	NOTCH2	NOTCH3	NOTCH4	MAML3	CREBBP	KAT2B
	EP300	HES1	HES2	HES3	HES4	HES5	HEY1	HEY2	DNER	PSEN2	NCOR1
	NCOR2	SPEN	KDM5A".split()
Hippo = "DCHS1	DCHS2	FAT1	FAT2	FAT3	FAT4	TAOK1	TAOK2
	TAOK3	SAV1	STK3	STK4	NF2	LATS1	LATS2	MOB1A	MOB1B
	WWC1	CRB1	CRB2	YAP1	TAZ	PTPN14	CSNK1E	CSNK1D
	TEAD2".split()
Myc = "MYC	MYCN	MYCL	MAX	MGA	MXD1	MXD3	MXD4	MXI1	MNT	MLX
	MLXIP	MLXIPL".split()
TGFb = "TGFBR1	TGFBR2	ACVR2A	ACRV1B	SMAD2	SMAD3
	SMAD4".split()
Nrf2 = "KEAP1	CUL3	NFE2L2".split()


# DNA damage response (DDR) pathways

BER = "APLF APTX	LIG1 LIG3	PARG PARP1 PARP3	PNKP POLB POLL	XRCC1
HMGB1	PCNA	POLD1 POLD2 POLD3 POLD4 RFC1 RFC2 RFC3 RFC4 RFC5	ALKBH1
APEX1 APEX2	FEN1	HMGB2	MBD4	MPG
MUTYH	NEIL1 NEIL2 NEIL3	NTHL1	OGG1	PARP2 PARP4	POLE POLE2 POLE3
POLE4	POLK	SMUG1	TDG TDP1	UNG	WRN".split()

NER = "LIG1	PCNA	POLD1 POLD2 POLD3 POLD4 RFC1 RFC2 RFC3
RFC4 RFC5 RPA1 RPA2 RPA3 RPA4	CCNH CDK7 CETN2	CUL3 CUL4A CUL5
DDB1 DDB2	ERCC1 ERCC2 ERCC3 ERCC4 ERCC5 ERCC6 ERCC8	GTF2H1
GTF2H2 GTF2H3 GTF2H4 GTF2H5	MMS19 MNAT1	POLE POLE2
POLE3 POLE4	RAD23A RAD23B	RBX1	TCEA1 TCEB1 TCEB2
UVSSA	XAB2 XPA XPC".split()

MMR = "LIG1	EXO1 HMGB1 MLH1 MLH3 MSH2 MSH3 MSH6 PCNA PMS1
PMS2 POLD1 POLD2 POLD3 POLD4 RFC1 RFC2 RFC3 RFC4 RFC5 RPA1 RPA2 RPA3
RPA4".split()

FA = "XRCC2	APITD1	BARD1 BLM BRCA1 BRCA2 BRE BRIP1
ERCC1	ERCC4	FAAP100 FAAP24 FAAP20	FAN1 FANCA FANCB FANCC FANCD2
FANCE FANCF FANCG FANCI FANCL FANCM	HELQ HES1	MAD2L2
PALB2	RAD51	RAD51C	RMI1 RMI2	SLX1A	SLX4	STRA13
TELO2 TOP3A TOP3B	UBE2T	USP1	WDR48".split()

HDR = "LIG1	MRE11A NBN	PARG PARP1	PARPBP	RAD50	TP53BP1	XRCC2 XRCC3
EXO1	PCNA	POLD1 POLD2 POLD3 POLD4 RFC1 RFC2 RFC3 RFC4 RFC5 RPA1 RPA2
RPA3 RPA4	BARD1 BLM BRCA1 BRCA2	BRIP1	DMC1 DNA2	EID3 EME1
EME2 ERCC1	FANCM FEN1	GEN1	H2AFX HELQ	HFM1	INO80 KAT5
MUS81	NFATC2IP NSMCE1 NSMCE2 NSMCE3 NSMCE4A	PALB2 PARP2	PAXIP1
POLH	POLQ PPP4C PPP4R1 PPP4R2 PPP4R4	RAD51 RAD51B RAD51C RAD51D
RAD52 RAD54B RAD54L	RBBP8	RDM1 RECQL RECQL4 RECQL5	RMI1 RMI2	RTEL1
SHFM1	SLX1A SLX1B SLX4 SMARCAD1 SMC5 SMC6	SPO11	SWSAP1	TOP3A TOP3B
UIMC1	WRN	ZSWIM7".split()

NHEJ = "DNTT	LIG4 MRE11A NBN NHEJ1 PARG PARP1 PARP3	PNKP POLB POLL POLM
```

```
PRKDC RAD50 RNF168 RNF8 TP53BP1    XRCC4 XRCC5 XRCC6
DCLRE1C                FAM175A
RIF1".split()

DR = "ASCC3                                        ALKBH2 ALKBH3
MGMT
".split()

TLS = "POLB  POLM           UBE2A        PCNA
HLTF     MAD2L2                              POLH POLI POLK POLN POLQ       RAD18
REV1 REV3L        SHPRH                   UBE2B UBE2N  UBE2V2   USP1  WDR48".split()

NP = "NUDT1 NUDT15 NUDT18 RRM1 RRM2".split()

Others_DDR = "ATM ATR ATRIP ATRX         CHAF1A CHEK1 CHEK2 CLK2    DCLRE1A
DCLRE1B      DUT                      GADD45A GADD45G           HUS1     MDC1     MPLKIP
MRPL40   NABP2                PER1 POLA1     POLG         PRPF19 RAD1 RAD17
RAD9A        RIF1   RNMT RRM2B  SETMAR      SLX4             TOPBP1 TP53 TREX1 TREX2
TYMS              PTEN TDP2 ENDOV SPRTN RNF4 SMARCA4 IDH1 SOX4 WEE1 RAD9B
AEN PLK3 EXO5 CDC5L BCAS2 PLRG1 YWHAB YWHAG YWHAE CDC25A CDC25B
CDC25C BABAM1 BRCC3 TTK SMARCC1 SWI5 MORF4L1 RNF169 HERC2".split()


# Splicing pathway

Spliceosome = "AGGF1       C9orf78         CCAR1         CD2BP2        CDC5L          CDK11A
        CDK12          CELF4CFAP20        CLK4  CWC22         DDX17          DDX18
        DDX20          DDX23          DDX26B        DDX27         DDX3X          DDX41
        DDX5 DDX50          DHX16          DHX35         DHX36          DHX9 EEF1A1
        EFTUD2         EIF2S2         ELAVL1         ELAVL2         ELAVL4         FAM58A
        FRA10AC1     FUBP1          FUBP3          GPATCH8        HNRNPCL1    HNRNPD
        HNRNPDL       HNRNPH3      HNRNPK         HNRNPL         IGF2BP3        INTS4 INTS7
        KIAA1429      KIN     MBNL2          MOV10          NCBP1          NELFE          NOVA1
        NSRP1          PABPC1         PCBP1 PCBP2 PCBP3 PHF5A          PLRG1          PPIG
        PPIL1  PPIL4  PRPF3 PRPF38B        PRPF39         PRPF40B        PRPF4B         PSIP1
        QKI    RALYL          RBBP6          RBM10          RBM15B         RBM25          RBM26
        RBM27          RBM7 RBM8A          RBMXRBMX2          RNF20SF1      SF3B1 SF3B2
        SF3B3 SKIV2L2        SNRNP200       SNRNP35        SNRNP48        SNRPD3         SNRPN
        SPEN  SRSF2 SRSF5 SYNCRIP        TCERG1         THOC5          THOC6          THOC7
        THRAP3         TIA1    TIAL1 TNPO1          TRIM24         TTC14U2AF1          U2AF2
        U2SURP         WBP11          WBP4 ZC3H13         ZC3H18         ZC3H4          ZCCHC8
        ZCRB1          ZMYM3          ZNF131         ZNF207         ZRSR2".split()


# Ubiquitination pathways

Ubq =
"ABTB1\tAHR\tAIRE\tAMBRA1\tAMFR\tANAPC1\tANAPC10\tANAPC11\tANAPC13\tANAP
C2\tANAPC4\tANAPC5\tANAPC7\tAPC2\tAREL1\tARIH1\tARIH2\tARNT\tASB1\tASB11\tAS
B12\tASB15\tASB2\tASB3\tASB4\tASB6\tASB7\tASB8\tASB9\tATG16L1\tAURKA\tBARD1\tB
CL6B\tBFAR\tBIRC2\tBIRC3\tBIRC6\tBIRC7\tBMI1\tBRAP\tBRCA1\tBRWD1\tBTBD9\tBTRC\
tCADPS2\tCAP1\tCAPS2\tCBL\tCBLB\tCBLC\tCBLL1\tCBX4\tCCNB1IP1\tCCNF\tCDC16\tCD
```

C20\tCDC23\tCDC26\tCDC27\tCDC34\tCDCA3\tCDH1\tCHFR\tCIAO1\tCISH\tCNOT4\tCORO
7\tCRBN\tCREBBP\tCSTF1\tCUL1\tCUL2\tCUL3\tCUL4A\tCUL4B\tCUL5\tCUL7\tCUL9\tDCA
F10\tDCAF11\tDCAF12\tDCAF13\tDCAF15\tDCAF16\tDCAF17\tDCAF4\tDCAF5\tDCAF6\tDC
AF7\tDCAF8\tDCUN1D1\tDCUN1D2\tDCUN1D3\tDCUN1D5\tDDB1\tDDB2\tDTL\tDTX1\tDT
X2\tDTX3\tDTX3L\tDTX4\tDZIP3\tE4F1\tEED\tEP300\tERCC8\tFANCL\tFBXL12\tFBXL14\tF
BXL15\tFBXL17\tFBXL19\tFBXL2\tFBXL20\tFBXL21\tFBXL3\tFBXL4\tFBXL5\tFBXL6\tFBX
L7\tFBXO10\tFBXO11\tFBXO15\tFBXO17\tFBXO18\tFBXO2\tFBXO22\tFBXO25\tFBXO27\tF
BXO28\tFBXO3\tFBXO30\tFBXO31\tFBXO32\tFBXO33\tFBXO4\tFBXO40\tFBXO42\tFBXO44
\tFBXO45\tFBXO6\tFBXO7\tFBXO8\tFBXO9\tFBXW10\tFBXW11\tFBXW2\tFBXW4\tFBXW5\
tFBXW7\tFBXW8\tFEM1B\tFUS\tFZR1\tG2E3\tGAN\tGNB2\tGNB2L1\tGRWD1\tHACE1\tHEC
TD1\tHECTD2\tHECTD3\tHECTD4\tHECW1\tHECW2\tHERC1\tHERC2\tHERC3\tHERC4\tHE
RC5\tHERC6\tHLTF\tHOXB4\tHUWE1\tING1\tING4\tIRF2BP1\tIRF2BPL\tITCH\tKAT2B\tKAT
NB1\tKBTBD13\tKBTBD7\tKCMF1\tKCTD11\tKCTD13\tKCTD21\tKCTD5\tKCTD6\tKDM2B\t
KEAP1\tKLHL10\tKLHL11\tKLHL12\tKLHL13\tKLHL17\tKLHL2\tKLHL20\tKLHL21\tKLHL2
2\tKLHL3\tKLHL40\tKLHL41\tKLHL42\tKLHL7\tKLHL8\tKLHL9\tKMT2A\tKMT2B\tLITAF\t
LNX1\tLRR1\tLRRC41\tLRSAM1\tLTN1\tMAP3K1\tMARCH1\tMARCH10\tMARCH11\tMAR
CH2\tMARCH3\tMARCH4\tMARCH5\tMARCH6\tMARCH7\tMARCH8\tMARCH9\tMDM2\tM
DM4\tMED8\tMGRN1\tMIB1\tMIB2\tMID1\tMID2\tMKRN1\tMNAT1\tMSL2\tMUL1\tMYCBP
2\tMYLIP\tNACC1\tNAE1\tNARF\tNEDD4\tNEDD4L\tNEDD8\tNEURL1B\tNEURL2\tNEURL
3\tNFX1\tNHLRC1\tNLE1\tNOSIP\tNSMCE1\tNSMCE2\tNUP43\tOSTM1\tPAF1\tPAFAH1B1\tP
AM\tPARK2\tPCGF1\tPCGF2\tPCGF3\tPDLIM2\tPDZRN3\tPELI1\tPELI2\tPELI3\tPEX10\tPEX
12\tPEX2\tPHIP\tPHRF1\tPJA1\tPJA2\tPLAA\tPML\tPOC1B\tPPARG\tPPIL2\tPRPF19\tPWP1\tR
AB40C\tRABGEF1\tRAD18\tRAG1\tRANBP2\tRASD2\tRBBP4\tRBBP5\tRBBP6\tRBBP7\tRBC
K1\tRBX1\tRC3H1\tRC3H2\tRCBTB1\tRCHY1\tREN\tRFFL\tRFPL4A\tRFWD2\tRFWD3\tRHO
BTB1\tRHOBTB2\tRHOBTB3\tRICTOR\tRING1\tRLIM\tRNF10\tRNF103\tRNF11\tRNF111\tR
NF113A\tRNF113B\tRNF114\tRNF115\tRNF121\tRNF122\tRNF123\tRNF125\tRNF126\tRNF128
\tRNF13\tRNF130\tRNF133\tRNF135\tRNF138\tRNF139\tRNF14\tRNF141\tRNF144B\tRNF146\
tRNF150\tRNF152\tRNF166\tRNF167\tRNF168\tRNF170\tRNF180\tRNF181\tRNF182\tRNF185\
tRNF186\tRNF19A\tRNF19B\tRNF2\tRNF20\tRNF208\tRNF213\tRNF216\tRNF217\tRNF220\tR
NF24\tRNF25\tRNF26\tRNF31\tRNF34\tRNF38\tRNF4\tRNF40\tRNF41\tRNF43\tRNF5\tRNF6\t
RNF7\tRNF8\tRNFT1\tRWDD3\tSAG\tSART1\tSH3RF1\tSH3RF3\tSHPRH\tSIAH1\tSIAH2\tSK
P2\tSMU1\tSMURF1\tSMURF2\tSNRNP40\tSNURF\tSOCS1\tSOCS2\tSOCS3\tSOCS4\tSOCS5\t
SOCS6\tSOCS7\tSPOP\tSPSB1\tSPSB2\tSPSB4\tSTC1\tSTUB1\tSYVN1\tTAF5\tTBL1X\tTBL1X
R1\tTCEB1\tTCEB2\tTLE1\tTLE2\tTLE3\tTMEM129\tTMF1\tTNFAIP1\tTOPORS\tTRAF2\tTR
AF3\tTRAF3IP2\tTRAF5\tTRAF6\tTRAF7\tTRAIP\tTRIM11\tTRIM13\tTRIM15\tTRIM17\tTRI
M2\tTRIM21\tTRIM22\tTRIM23\tTRIM24\tTRIM25\tTRIM26\tTRIM27\tTRIM28\tTRIM3\tTRI
M31\tTRIM32\tTRIM33\tTRIM34\tTRIM35\tTRIM36\tTRIM37\tTRIM38\tTRIM39\tTRIM4\tTRI
M40\tTRIM41\tTRIM43\tTRIM45\tTRIM46\tTRIM47\tTRIM5\tTRIM50\tTRIM51\tTRIM54\tTRI
M56\tTRIM59\tTRIM6\tTRIM62\tTRIM63\tTRIM65\tTRIM68\tTRIM71\tTRIM72\tTRIM73\tTRI
M74\tTRIM8\tTRIM9\tTRIP12\tTRPC4AP\tTSG101\tTTC3\tUBA1\tUBA2\tUBA3\tUBA5\tUBA
6\tUBA7\tUBE2A\tUBE2B\tUBE2C\tUBE2D1\tUBE2D2\tUBE2D3\tUBE2D4\tUBE2E1\tUBE2E
2\tUBE2E3\tUBE2F\tUBE2G1\tUBE2G2\tUBE2H\tUBE2I\tUBE2J1\tUBE2J2\tUBE2K\tUBE2L3\
tUBE2L6\tUBE2M\tUBE2N\tUBE2O\tUBE2Q1\tUBE2Q2\tUBE2R2\tUBE2S\tUBE2T\tUBE2U\t
UBE2V1\tUBE2V2\tUBE2W\tUBE2Z\tUBE3A\tUBE3B\tUBE3C\tUBE3D\tUBE4A\tUBE4B\tUB
OX5\tUBR1\tUBR2\tUBR3\tUBR4\tUBR5\tUBR7\tUFC1\tUFL1\tUHRF1\tUHRF2\tVHL\tVPRB
P\tVPS18\tVPS41\tWDR11\tWDR12\tWDR26\tWDR5\tWDR53\tWDR59\tWDR5B\tWDR61\tW
DR76\tWDR82\tWDTC1\tWSB1\tWSB2\tWWP1\tWWP2\tXIAP\tZBTB18\tZC3HC1\tZFP91\tZ
MIZ1\tZMIZ2\tZNF230\tZNF645\tZNRF1\tZNRF2\tZNRF3\tZNRF4\tZSWIM2\t".split()

Dubq = "ALG13 ATXN3 ATXN3L BAP1 BRCC3 COPS5 COPS6
CYLD EIF3F EIF3H JOSD1 JOSD2 MPND MYSM1 OTUB1 OTUB2
OTUD1 OTUD3 OTUD4 OTUD5 OTUD6A OTUD6B

OTUD7A OTUD7B PAN2 PARP11 PRPF8 PSMD14 PSMD7
STAMBP STAMBPL1 TNFAIP3 UCHL1 UCHL3 UCHL5
USP1 USP10 USP11 USP12 USP13 USP14 USP15 USP16 USP17L2 USP18 USP19
USP2 USP20 USP21 USP22 USP24 USP25 USP26 USP27X USP28 USP29 USP3
USP30 USP31 USP32 USP33 USP34 USP35 USP36 USP37 USP38 USP39 USP4 USP40
USP41 USP42 USP43 USP44 USP45 USP46 USP47 USP48 USP49 USP5 USP50 USP51
USP53 USP54 USP6 USP7 USP8 USP9X USP9Y VCPIP1 YOD1
ZRANB1 FAM63A FAM63B FAM188A FAM188B".split()

Metabolic pathways

Amino_acid = "SLC3A2 GOT1 GOT2 ACAD8 ACADSB NDUFAB1 ASPA
AGXT2 GPT ARG2 ARG1 ASL ASNS ASS1 SAT1 TAT BCAT2
BCAT1 BCKDK BHMT BBOX1 CBS CNDP2 CTH CPS1
CRYM CSAD CDO1 HIBADH ALDH7A1 AMD1 HDC ODC1 DDC
ALDH9A1 GLUD1 GLUD2 QDPR SLC25A10 DLD DBH SHFM1
FAH IL4I1 FOLH1 FTCD GAMT GATM GCDH GCSH GLDC AMT CGA
GNMT GLUL GLS GLS2 SHMT1 GCLM GCLC HAO1 HSD17B10 HGD
HNMT HPD HAL UROC1 IDO1 INMT DIO3 IVD GCAT CKB CKM
CKMT2 CKMT1A KYNU SLC7A5 LIAS LIPT1 GSTZ1 SLC45A2
AIMP1 AIMP2 EEF1E1 MCCC1 MCCC2 MTR MAT1A
ALDH6A1 MTAP MTRR NAALAD2 NNMT NQO1 OAT OAZ2 OAZ3 OAZ1
DBT BCKDHA BCKDHB AZIN1 SLC25A21 OGDH DLST DLAT PDHA1
PDHB PDHX SLC25A15 SLC25A2 OTC DAO DDO PAH PCBD1
PNMT PAPSS1 PYCR1 PRODH PSMC5 PSMA8
ALDH4A1 OCA2 RPS4Y2 SLC6A7 SLC6A8 SLC6A11 SLC6A12
AHCY SECISBP2 EEFSEC PHGDH PSPH PSAT1 SLC5A5 AANAT
PIPOX AGMAT SRM SMS AGXT SQRDL SUOX DARS EPRS IARS
KARS MARS QARS RARS SARS TDO2 SERINC3 SERINC1 ACAT1
TST TMLHE TXNRD1 TSHB TYRP1 DCT TYR PSMD14
NAGS HIBCH AASS AADAT KMO ACMSD RPS10 RPS11 RPS12
RPS13 RPS14 RPS15 RPS16 RPS17 RPS18 RPS19 RPS15A RPS2 RPS20 RPS21
RPS23 RPS24 RPS25 RPS26 RPS27 RPS27A RPS28 RPS29 RPS3 FAU RPS3A
RPS4X RPS4Y1 RPS5 RPS6 RPS7 RPS8 RPS9 RPSA RPLP0 RPLP1
RPLP2 RPL10 RPL10A RPL11 RPL12 RPL13 RPL13A RPL14 RPL15 RPL17
RPL18 RPL18A RPL19 RPL21 RPL22 RPL23 RPL23A RPL24 RPL26 RPL26L1
RPL27 RPL27A RPL28 RPL29 RPL3 RPL3L RPL30 RPL31 RPL32 RPL34 RPL35
RPL35A RPL36 RPL37 RPL37A RPL38 RPL39 RPL4 UBA52 RPL41
RPL36A RPL5 RPL6 RPL7 RPL7A RPL8 RPL9 AUH PSMC1
PSMC4 PSMC2 PSMC3 PSMC6 PSMA1 PSMA2
PSMA3 PSMA4 PSMA5 PSMA6 PSMA7 PSMB1
PSMB2 PSMB3 PSMB4 PSMB5 PSMB6 PSMB7
PSMB8 PSMB9 PSMB10 PSMD1 PSMD2 PSMD3
PSMD4 PSMD5 PSMD6 PSMD7 PSMD8 PSMD9
PSMD10 PSMD11 PSMD12 PSMD13 PSME1 PSME2
PSME3 PSMF1 RPL39L SERINC2 TPH2 PAOX ADO GRHPR
CCBL1 ALDH18A1 RPS27L SMOX LARS AFMID AMDHD1
APIP ASRGL1 BHMT2 C9orf41 DHTKD1 DUOX1 DUOX2
MRI1 ENOPH1 ETHE1 GADL1 GSR IDO2 CCBL2 ASPG
ADI1 NMRAL1 PYCR2 PYCRL PPM1K PSMB11 PSME4
PSTK RPL10L RPL22L1 RPL36AL SCLY SEPSECS CARNS1

```
	LIPT2	SERINC4	SERINC5	PAPSS2	TH	TPO	TPH1	ASMT	DIO1
	IYD	GPT2	DIO2	ADC".split()

Carbohydrates = "PPP2R1A	PPP2R1B	PGD	PGLS	AAAS	GOT1	GOT2	AKR1A1
	AKR1B1	ALDOA	ALDOB	ALDOC	AMY2B	AMY2A
	AMY1A	NAGLU	ARSB	B3GAT1	B3GAT2	B3GAT3	B3GNT1
	B3GNT2	B3GNT3	B4GALT1	B4GALT2	B4GALT3	B4GALT4
	B4GALT5	B4GALT6	B4GALT7	GLB1	GUSB	CALM1	CD44
	SLC25A12	SLC25A13	CRYL1	DERA	ALDH1A1	SORD	SLC25A10
	SLC26A2	ENO1	ENO3	ENO2	EXT1	EXT2	FBP1	FBP2	PFKFB1	PFKFB2
	PFKFB3	PFKFB4	FGF21	FMOD	GAPDH	GAPDHS	G6PD	GPI
	G6PC	GALNS	GALK1	GALT	GALE	GCKR	AGL	GNS	GLCE	GBE1
	GYG1	GNPDA1	GPC1	GPC3	GPC4	GPC5	GPC6	SLC2A1	SLC2A2
	SLC2A3	SLC2A4	SLC2A5	GYG2	GYS1	GYS2	HAS1	HAS2	HAS3
	HEXA	HEXB	HMMR	NDST1	NDST2	HK1	HK2	HK3	HYAL2
	IDS	IDUA	NUP160	PFKM	PFKL	PFKP	PRKACA	PRKACB	PRKACG
	KERA	KHK	PHKA1	PHKA2	PHKB	PHKG1	PHKG2	PRPS1
	PRPS2	PRPS1L1	PKLR	LALBA	LCT	LUM	GAA	MAN2B1	MAN2B2
	MAN2C1	SLC25A11	MANBA	MDH1	MDH2	MGAM	OGN	ABCC5
	NUP107	NUP153	NUP214	SLC9A1	NUP37	NUP43
	NUP50	NUP62	NUP88	OMD	PPP2CA	PPP2CB	HSPG2
	ACAN	NCAN	VCAN	PGK1	PGK2	PGM1	BGN	DCN	PYGL	PYGM	PYGB	PGAM1
	PGAM2	BPGM	PCK1	PCK2	PAPSS1	PRELP	PC	RAE1	RANBP2
	RPIA	SLC26A1	SDC1	SDC2	SDC3	SDC4	ST3GAL1	ST3GAL2
	ST3GAL4	ST3GAL3	SLC5A1	SLC5A2	SLC5A3	SLC5A4
	SGSH	SI	TALDO1	TKT	TPR	TREH	SLC25A1	NUP155	RPS27A
	UBA52	CHIA	CHIT1	NUP133	NUP54	NUPL1	RPE	TPI1
	AGRN	DCXR	EPM2A	KIAA1199	NUP205	NHLRC1	NUP93
	NUP98	BCAN	SLC45A3	STAB2	SEH1L	ST3GAL6	NUPL2
	NUP85	NUP35	ADPGK	B3GNT4	B3GNT7	B3GALT6
	CSGALNACT1	CSGALNACT2	CHPF2	CHSY1	CHPF	CHSY3
	CHST1	CHST2	CHST3	CHST5	CHST6	CHST7
	CHST9	CHST11	CHST12	CHST13	CHST14	CSPG4
	CSPG5	DSEL	DSE	GLB1L	GLYCTK	GPC2	SLC2A14	HS6ST1
	HS6ST2	HS6ST3	HGSNAT	HPSE2	HPSE	HS2ST1	HYAL1
	HYAL3	LYVE1	NDST3	NDST4	NUP188	HS3ST1
	HS3ST2	HS3ST3A1	HS3ST3B1	HS3ST4	HS3ST5	HS3ST6
	PGM2	NUP210	PPP1R3C	RSC1A1	SLC35B2	SLC35B3	SLC35D2
	SLC5A10	CHST15	UST	XYLB	XYLT1	XYLT2	G6PC2	G6PC3
	SLC5A9	GNPDA2	POM121	POM121C	SLC37A4	PAPSS2
	GCK	PPP2R5D	UGP2	UBC	UBB".split()

Energy = "PPP2R1A	PPP2R1B	ADRA2A	PRKAA2	PRKAB2	PRKAG2
	ACLY	CHRM3	SLC25A4	SLC25A5	SLC25A6	ADIPOQ	ARL2
	CACNA1A	CACNA1E	CACNB2	CACNB3	ACACB	ADCY1
	ADCY2	ADCY3	ADCY4	ADCY5	ADCY6	ADCY7
	ADCY8	ADCY9	PFKFB1	FASN	GNA11	GNA14	GNA15
	GNAS	GNB1	GNB2	GNB3	GNB4	GNGT1	GNG3	GNG4	GNG5	GNG7	GNG8
	GNG10	GNG11	GNG12	GNG13	GNGT2	GNAI1	GNAI2
	GNAQ	GCGR	GCG	FFAR1	SLC2A1	SLC2A2	INS	ITPR3	KCNJ11
	PRKAR1A	PRKAR1B	PRKAR2A	PRKAR2B	PRKACA	PRKACB
	PRKACG	PRKCA	PKLR	MARCKS	MLX	PPP2CA	PPP2CB
```

```
        PLCB1   PLCB2   PLCB3   AGPAT1  RAP1A   AHCYL1
        STK11 SYT5 TALDO1 TKT VAMP2 MLXIPL ADIPOR1
        ADIPOR2 GNG2 ADRA2C CACNA2D2 ARL2BP CACNA1C GLP1R
        AKAP5   KCNS3   IQGAP1  KCNB1   KCNG2   GNB5
        RAPGEF3 SNAP25  STXBP1  CACNA1D ABCC8   ACACA
        ITPR2 ITPR1 PPP2R5D STX1A   RAPGEF4 KCNC2".split()

Lipid = "HSD3B1     HSD3B2  AP2A1   AP2A2   AP2B1 AP2M1     A2M
        AP2S1 PRKAA2 PRKAB2 PRKAG2 ABCB11 ABCA1 ABCG5
        ABCG8   DBI     ACADL   ACADM   ACADS   ACADVL  ACHE
        ACLY SCD NDUFAB1 AGPS PLIN2 FDXR FDX1 GLA AHR AKR1C1
        AKR1C2  AKR1C3  AKR1C4  AKR1D1  ALB     AKR1B1  ABCD1
        AMACR   AGT     ANGPTL3 ANGPTL4 APOA1   APOA2   APOA4
        APOB APOC1 APOC2 APOC3 APOC4 APOE APOF ACER3
        LPA ARF1 ARF3 ARNT2 ARNT ARSA ARSB ARSD ARSE ARSF ASAH1
        SMPD1   ACOT7   CEL     BDH1 GLB1 BHMT BMX CYP11A1
        CYP39A1 ACOT12  CRAT B4GALNT1 ACOX1 ACOX2 ACOX3
        CAV1 NFYB CREBBP CD36 CDS1 CDS2 UGCG CETP ACOT9 CCNC
        UGT8 BCHE CIDEA CLOCK   CPNE1   CPNE3   CPNE6
        CPNE7 ACACB POMC CLPS COQ3 COQ6 COQ7 CYP1A1 CYP1A2
        CYP1B1  CYP27A1 CYP46A1 CYP4B1  CYP4A11 CYP51A1
        CYP7A1  CYP7B1  CYP8B1  CYP2C8  CYP2C9  CYP2C19
        CYP4F2  CYP4F3  CYP4F11 CYP2J2  CYP11B1 CYP11B2
        CYP21A2 CPT1A CPT2 CYP17A1 CPT1B CYP19A1 MED17
        MED7 SPTLC3 FITM2 CTGF PCYT1A PCYT1B MID1IP1
        ALDH7A1 DAB1 GNPAT MLYCD DECR1 DGAT1
        ALDH3B1 ALDH3B2 HSD17B1 HSD17B2 HSD17B3 HSD17B4
        HSD17B8 DHCR7 CBR1 DHCR24 HSD11B1 HSD11B2 EBP
        HADHA   HADHB   ECHS1   EHHADH  ETNK1   ETNK2
        ELOVL1  ELOVL2  ELOVL3  ELOVL4  MVD TM7SF2 SQLE
        LSS ESRRA FAAH FABP4 FABP7 FABP5 FABP3 FABP2 FABP1 FASN FDFT1
        FGF21 ALOX5AP FDPS CIDEC FURIN KDSR FYN PIKFYVE
        G0S2 GALC GGPS1 GGT1 GGT5 GK2 GBA CGA GLIPR1 GK
        GLTP GPD1 GPD2 PNPLA4 GPX1 GPX2 GPX4 TECR HAO2 HADH ALAS1
        HEXA HEXB HMGCS2 HMGCS1 HACL1 HRASLS5 HRASLS
        HRASLS2 PLA2G16 EPHX2 IDH1 IDI1 IDI2 FABP6 KPNB1
        INSIG1  PRKACA  PRKACB  PRKACG  CSNK1G2 CSNK2A2
        CSNK2B GPCPD1 CHKB CHKA MVK PRKD1 PRKD3 PRKD2
        PTGR1 LTC4S LCAT SPTLC1 SPTLC2 ACSL1 ACSL3
        ACSL4 ACSL5 ACSL6 LDLR VLDLR LIPA PNLIPRP1
        LIPG LIPF LIPC LPL PNLIP LIPE LTA4H ALOX15 ALOX5 ALOX12
        ALOX12B LPIN1 LPIN2 LPIN3 LHB ALOX15B DMGDH ME1
        SLC25A20 DPEP1 ABCB4 MED6 STARD3NL MAPKAPK2 STARD3
        ABCC1 ABCC3 MBTPS1 MBTPS2 MTM1 MTTP MTMR1
        MTMR2   MTMR3   MTMR6   MTMR7   MUT     NCOA2   NEU1
        NEU2 NEU3 TRIB3 SCP2 NPAS2 NPC1 NPC2 NR1D1 NRF1 NR1H2
        NSDHL SMPD2 SLC10A2 SLC10A1 CROT OSBPL1A OSBPL2
        OSBPL3  OSBPL5  OSBPL6  OSBPL7  OSBPL8  OSBPL9
        OSBPL10 OSBP PIK3CA PIK3CB PIK3CD PIK3CG EP300
        PIK3R3  PIK3R1  PIK3R2  PLA2G1B PLA2G3  PLA2G4A
        PLA2G5  PLA2G6  PLA2G2A PLA2G2D PLA2G2E PLA2G2F
        PLA2G10 PLA2G12A PHYH PCCA PCCB PCSK5 STARD10 PCYT2
```

P4HB PEMT HPGDS HPGD PTGS1 PTGS2 PTGDS PI4KA PIP4K2A
CDIPT PIK3C2B PIK3C2G AGPAT1 AGPAT2 AGPAT3 AGPAT4
AGPAT5 PLD2 PLIN1 GPAM PLTP SLC25A17 PMVK ACOT13 PON1
PON2 PON3 PPP1CA PPP1CB PPP1CC PPARA PPARD
ALPI PCTP PITPNB PPT1 PPT2 CTSA PTDSS1 ACOT8 ACOT2
PTEN PTGES PTGIS PTPN13 RAB14 RAB5A RELN RGL1
RORA RXRA SLCO1A2 SLCO1B1 SLCO1B3 SEC23A SEC24A
SEC24B SEC24C SEC24D SRD5A1 GM2A PSAP SAR1B
SCAP OXCT1 FHL2 SOAT1 SOAT2 SP1 SPHK1 SPHK2
MED21 SREBF2 STAR STARD4 STARD5 STARD6 STARD7
STS SULT2A1 MED22 SYNJ1 SYNJ2 MED24 THRAP3 MED12
MED13 TBL1XR1 TBL1X TEAD1 TEAD2 TEAD3
TEAD4 PTGES3 TAZ TBXAS1 ACOT11 THRSP ACAA1
ACAT1 ACAA2 PLIN3 RARRES3 TPTE TNFRSF21 TXNRD1
SLC25A1 VAPA VAPB HDLBP SLC27A2 LPGAT1 FIG4 YAP1
ZDHHC8 CDK8 RAN RAB4A PLD1 LBR MCEE HMGCL PNPLA3
AMN CERK TMEM55B CRLS1 DDHD1 MMAA PI4KB INPP5J
PCSK9 EPT1 SGPL1 SH3KBP1 INPP5K SIN3A SIN3B MED31
SGPP1 SGPP2 SCARB1 SUMF1 SUMF2 MED20 HSD3B7
B3GALNT1 ARNTL BMP1 COL4A3BP CHDH ACOT1 ALDH3A2
MCAT HMGCR INPP5E MGLL MYLIP PLA2G4C PIP4K2B
PLEKHA1 PLEKHA2 PLEKHA3 PLEKHA4 PLEKHA5 PLEKHA6
PPARGC1A PTDSS2 ACOT4 RUFY1 SARDH OLAH SGMS1
SGMS2 SREBF1 ACAT2 BAAT THEM4 LDLRAP1 APOA5
PIK3C3 PIK3R4 CH25H SLC27A5 TGS1 CSNK2A1 CYP4F22
MED25 MED14 MED30 MED4 AACS ABHD3 ABHD4
ABHD5 ACBD4 ACBD5 ACBD6 ACBD7 ACSBG1
ACSBG2 ACAD10 ACAD11 ACOT6 ACOXL ACSF2
ACSF3 ACSM3 ACSS3 NCEH1 AGK ANKRD1 ARSG
ARSH ARSI ARSJ ARSK ARV1 ACER2 ASAH2 ACER1 AWAT1
AWAT2 BDH2 CARM1 CBR4 CDK19 CEPT1 CHD9 CHPT1
COQ2 COQ5 COQ9 CREB3L3 SLC44A1 SLC44A2 SLC44A3 SLC44A4
SLC44A5 CUBN DDHD2 DECR2 DEGS1 DEGS2
DGAT2L6 DGAT2 HSD17B11 HSD17B12 HSD17B13 HSD17B14
PDSS2 DPEP2 DPEP3 PDSS1 ELOVL5 ELOVL6 ELOVL7 ENPP6 ENPP7
CES3 ESYT1 ESYT2 ESYT3 FAAH2 FABP9 FAR1 FAR2
FADS1 FADS2 GBA2 GDE1 GDPD1 GDPD3 GDPD5 GLB1L
GLTPD1 GPD1L GRHL1 GPIHBP1 INPP4A INPP4B
INSIG2 LIPH LIPI LIPJ LIPK LIPM LIPN PNLIPRP3 LMF1 LMF2
LSR LCLAT1 MBOAT1 MBOAT2 LPCAT3 MBOAT7 MED13L
MED10 MED11 MED18 MED19 MED28 MED29
MED9 MFSD2A MOGAT1 MOGAT2 MOGAT3 MTF1 MTMR4
MTMR14 NPC1L1 SMPD3 SMPD4 NUDT19 NUDT7
ORMDL1 ORMDL2 ORMDL3 PIK3C2A PI4K2A PI4K2B
PLA2G4D PLA2G4E PLA2G4F LPCAT1 LPCAT2 PECR PTGES2
PHOSPHO1 PIK3R5 PIK3R6 PIP4K2C PIP5K1A PIP5K1B
PIP5K1C PITPNM1 PITPNM2 PITPNM3 PLEKHA8 PLA1A
PLA2R1 PLB1 PLBD1 AGPAT6 LPCAT4 AGPAT9 PLD3
PLD4 PNPLA2 PNPLA5 PNPLA6 PNPLA7 PNPLA8 GPAT2
ACP6 PPM1L PPARGC1B PTPLAD1 PTPLAD2 PTPMT1 PEX11A
SLC27A1 SLC27A3 TECRL SRD5A3 SACM1L SCD5 INPP5D
INPPL1 SMARCD3 TNFAIP8 THEM5 TMEM86B TNFAIP8L1

```
TNFAIP8L2	TPTE2	FITM1	VAC14	WWTR1	PLD6	PTGR2	CHAT
	C10orf129	AHRR	FAM120B	HMGCLL1	PGS1	INPP5F	TNFAIP8L3
	NCOA6	CYP4A22	FABP12	PTPLA	PTPLB	HDAC3
	NCOR1	MED26	CLTA	NFYA	PCSK6	PPARG	ABCG1
	HSD17B7	CLTC	OCRL	ACACA	NR1H3	NFYC	MED1	NCOA1
	MED27	CYP2U1	TIAM2	MED8	NR1H4	MED15	MED23
	MED16	NCOR2	NCOA3	PISD	NEU4	AKR1B15".split()

Nucleotide = "NT5C2	NT5E	ADA	ADK	AGXT2	AMPD1	AMPD2	AMPD3
	APRT	UPB1	CAT	CDA	DCK	DCTD	DGUOK	DPYD	DPYS	ENTPD1
	ENTPD2	ENTPD3	ENTPD4	ENTPD5	ENTPD6	GLRX	GMPR
	GMPR2	GPX1	GMPS	GDA	HPRT1	IMPDH1	IMPDH2	AK1	AK2
	AK5	CMPK1	GUK1	TK2	DTYMK	NME1	NME2	NME4	NUDT5
	PNP	PPAT	GART	PFAS	PAICS	ADSL	ATIC	ADSS	CAD	UMPS	DHODH
	RRM1	RRM2	TXN	TXNRD1	TYMP	TYMS	UCK1	UCK2	UPP1	XDH	TK1
	ITPA	AK7	NT5C	NT5M	ADSSL1	UPP2	NT5C1A	NT5C1B	DUT
	NUDT13	POMP	NUDT1	ADAL	DDX31	ENTPD7	ENTPD8
	GSR	LHPP	NUDT15	NUDT16	CTPS2	RRM2B	DCTPP1	NUDT18
	NUDT9".split()

TCA_cycle = "ACO2	NDUFAB1	ATP5G1	ATP5G2	ATP5G3	ATP5A1
	ATP5B	ATP5D	ATP5E	ATP5F1	ATP5C1	ATP5I	ATP5J2	ATP5L
	ATP5O	ATP5H	ATP5J	ATP5S	BSG	SDHC	NDUFAF1	CS
	COX7A2L	COX5A	COX5B	COX6A1	COX6B1	COX6C
	COX7B	COX7C	COX8A	COX11	COX4I1	CYC1	CYCS
	SDHA	SDHB	SDHD	DLD	ETFA	ETFB	FH	IDH3A	IDH3B	IDH3G	IDH2	LDHA
	LDHB	LDHC	LDHAL6B	GLO1	LRPPRC	MDH2	SLC16A1	SLC16A8
	SLC16A3	NDUFA7	NDUFC2	NDUFA12	NDUFB3	NDUFA6
	NDUFB4	NDUFA13	NDUFB6	NDUFB7	NDUFB9	NDUFA2
	NDUFA3	NDUFB8	NDUFB10	NDUFB2	NDUFC1	NDUFA1
	NDUFB1	NDUFS5	NDUFB5	NNT	NDUFS1	NDUFV1	NDUFS2
	NDUFA10	NDUFA9	NDUFA5	NDUFS3	NDUFV2	NDUFS8
	NDUFS7	NDUFA4	NDUFS6	NDUFV3	NDUFA8	NDUFS4
	OGDH	DLST	DLAT	PDHA1	PDHB	PDHX	PDK1	PDK2	PDK3	PDK4	PDP1
	PDP2	PPARD	RXRA	SUCLA2	SUCLG2	SCO1	SCO2	SUCLG1
	SURF1	TRAP1	UCP1	UCP2	SLC25A27	UQCRC1	UQCRC2
	UQCRB	UQCRH	UQCRFS1	UQCRQ	UQCR10	UQCR11
	ACAD9	NDUFAF4	NUBPL	NDUFB11	COX18	TACO1
	LDHAL6A	NDUFA11	ECSIT	NDUFAF3	COX16	COX19	COQ10A
	COQ10B	D2HGDH	ADHFE1	L2HGDH	NDUFAF2	TMEM126B
	PDPR	SLC25A14	UCP3	ETFDH	HAGH".split()

Vitamin_cofactor = "NT5E	ABCD4	AOX1	AKR1B10	AKR1C1	AKR1C3
	AKR1C4	APOA1	APOA2	APOA4	APOB	APOC2	APOC3
	APOE	APOM	HLCS	BST1	BTD	MTHFD1	CD38	CTRC	ACACB	CLPS
	CYP24A1	CYP27B1	CYP8B1	CTRB1	CYB5A	SLC25A19
	DHFR	FASN	SLC19A1	FOLR2	ALDH1L1	MTHFS	SLC25A16
	GPHN	SHMT1	SHMT2	GPC1	GPC3	GPC4	GPC5	GPC6	GSTO1
	SLC2A1	SLC2A3	GIF	LDLR	LGMN	LPL	PNLIP	LRP1	LRP2	MCCC1
	MCCC2	MTR	SLC25A32	MOCS3	ABCC1	MOCS2	MTHFD2
	MTHFR	MTRR	MUT	QPRT	COASY	CYB5R3	NNMT	ENPP1	ENPP2
	ENPP3	NAMPT	PCCA	PCCB	PDXK	HSPG2	PTGS2	PANK2	PANK3
```

```
PANK4   ACP5  NADK PARP4 PTGIS PC   RBP1  RBP2  RBP4  SDC1  SDC2
SDC3  SDC4  SLC5A6   LRP12 SLC23A1   SLC23A2   TCN1  TCN2  SLC19A2
PRSS1 PRSS3 TTR   VNN1  VNN2  GC   LRAT  AMN  PARP9 BCMO1   BCO2
GSTO2   MMAA   MMAB   NMNAT1   NMNAT2   PNPO  RDH11
RFK  THTPA    AGRN LRP10 LRP8  NMNAT3   PPCS  TPK1  VKORC1
PPCDC   FLAD1   NADSYN1   FPGS  AASDHPPT  SLC19A3   SLC46A1
CYP2R1   APOA1BP   ALDH1L2   MMADHC   CD320 CTRB2   CUBN
DHFRL1   GPC2  GPIHBP1   LMBRD1   MMACHC   MOCOS   NFS1
NUDT12   PARP10   PARP14   PARP16   PARP6 PARP8 PDZD11
RFT1  PLB1  NAPRT1   SLC22A13   UBIAD1   VKORC1L1  MTHFD2L
SLC5A8   CARKD   MTHFD1L   ACACA   PANK1   MOCS1".split()


# All pathways

pathways = [["RTK_RAS", "cell_cycle", "p53", "PI3K_Akt", "b_catenin_Wnt", "Notch", "Hippo", "Myc", "TGFb", "Nrf2", "BER", "NER", "MMR", "FA", "HDR", "NHEJ", "DR", "TLS", "NP", "Others_DDR", "Spliceosome", "Ubq", "Dubq", "Amino_acid", "Carbohydrates", "Energy", "Lipid", "Nucleotide", "TCA_cycle", "Vitamin_cofactor"], [RTK_RAS, cell_cycle, p53, PI3K_Akt, b_catenin_Wnt, Notch, Hippo, Myc, TGFb, Nrf2, BER, NER, MMR, FA, HDR, NHEJ, DR, TLS, NP, Others_DDR, Spliceosome, Ubq, Dubq, Amino_acid, Carbohydrates, Energy, Lipid, Nucleotide, TCA_cycle, Vitamin_cofactor]]


path_gene_mut = pd.DataFrame(columns=["Pathway", "Gene", "Mutation rate (%)"])

c = 0
j = -1
for pathway in pathways[1]:
    j += 1
    for g in pathway:
        for ind in genes.Gene.index:
            if genes.Gene[ind] == g.upper() and genes.iloc[ind]["Driver Gene"] == "Yes":
                c += 1
                path_gene_mut.loc[c] = pathways[0][j], g.upper(), genes.iloc[ind]["Frequency"]
                break

path_gene_mut.to_csv("all_pathways_drivers.csv")
```

Program that extends the final produced matrix of the previous program in order to comprise the genes of one more pathway (immunomodulators).

```
cd ~\python programming\2

import pandas as pd

genes = pd.read_csv("gene_mutation_frequencies.csv")

i = -1
```

```
for freq in genes.Frequency:
    i += 1
    genes.Frequency[i] = float(freq.replace(",", "."))


path_gene_mut = pd.read_csv("all_pathways_drivers.csv")
path_gene_mut = path_gene_mut.drop(columns="Unnamed: 0")

# Immunomodulators

Immunomodulators = "ADORA2A   ARG1  BTLA  BTN3A1    BTN3A2    CCL5  CD27
        CD274 CD276 CD28  CD40  CD40LG    CD70  CD80  CTLA4     CX3CL1
        CXCL10    CXCL9     EDNRB     ENTPD1    GZMA HAVCR2     HLA-A
        HLA-B     HLA-C     HLA-DPA1  HLA-DPB1  HLA-DQA1  HLA-DQA2
        HLA-DQB1  HLA-DQB2  HLA-DRA   HLA-DRB1  HLA-DRB3  HLA-DRB4
        HLA-DRB5  HMGB1     ICAM1     ICOS  ICOSLG    IDO1  IFNA1 IFNA2
        IFNG  IL10  IL12A IL13  IL1A  IL1B  IL2   IL2RA IL4   ITGB2 KIR2DL1
        KIR2DL2   KIR2DL3   LAG3  MICA  MICB  PDCD1     PDCD1LG2  PRF1
        SELP  SLAMF7    TGFB1     TIGIT TLR4  TNF   TNFRSF14  TNFRSF18
        TNFRSF4   TNFRSF9   TNFSF4    TNFSF9    VEGFA     VEGFB
        VSIR  VTCN1".split()

c = len(path_gene_mut)
for g in Immunomodulators:
    for ind in genes.Gene.index:
        if genes.Gene[ind] == g.upper() and genes.iloc[ind]["Driver Gene"] == "Yes":
            path_gene_mut.loc[c] = "Immunomodulators", g.upper(), genes.iloc[ind]["Frequency"]
            c += 1
            break

path_gene_mut.to_csv("all_pathways_drivers_complete.csv")
```

Program that creates a figure containing 29 horizontal bar graphs, each showing the mutational rate of the cancer genes of a different pathway.

```
cd ~\python programming\2

import pandas as pd
import matplotlib.pyplot as plt
import numpy as np

genes = pd.read_csv("all_pathways_drivers_complete.csv")

# How many out of the 31 pathways are represented in genes Dataframe ?
len(set(list(genes.Pathway)))
# None of the genes of DR and NP pathways' gene lists are characterized as
# cancer genes by oncoKB database. Thus, from the following pathways list,
# these two pathways were excluded.
```

```
pathways = ["RTK_RAS", "cell_cycle", "p53", "PI3K_Akt", "b_catenin_Wnt", "Notch", "Hippo",
"Myc", "TGFb", "Nrf2", "BER", "NER", "MMR", "FA", "HDR", "NHEJ", "TLS", "Others_DDR",
"Spliceosome", "Ubq", "Dubq", "Amino_acid", "Carbohydrates", "Energy", "Lipid", "Nucleotide",
"TCA_cycle", "Vitamin_cofactor", "Immunomodulators"]
titles = ["RTK/RAS", "Cell cycle", "p53", "PI3K Akt", "b catenin/Wnt", "Notch", "Hippo", "Myc",
"TGFb", "Nrf2", "BER", "NER", "MMR", "FA", "HDR", "NHEJ", "TLS", "Other DDR",
"Splicing", "Ubiquitination", "Deubiquitination", "Amino acids", "Carbohydrates", "Energy",
"Lipids", "Nucleotides", "TCA cycle", "Vitamins", "Immunomodulators"]

fig, ax = plt.subplots(3, 10)
fig.set_size_inches(30, 40)
plt.subplots_adjust(wspace=3.5)
ax[2, 9].remove()

plots = []

colors = ["r", "r", "r", "r", "r", "r", "r", "r", "r", "r", "b", "b", "b", "b", "b", "b", "b", "b", "m", "g",
"g", "c", "c", "c", "c", "c", "c", "c", "y"]
ind = -1
for i in range(3):
    for j in range(10):
        if i == 2 and j == 9:
            continue
        else:
            ind += 1
            z = genes[genes.Pathway == pathways[ind]].sort_values(by="Mutation rate (%)",
ascending=False)
            g = list(z.Gene)
            y = np.arange(len(g))
            x = np.array(z["Mutation rate (%)"])
            p = ax[i][j].barh(y, x, align='center', color=colors[ind])

            # Add only the first plot of each category to plots list
            if ind in [0, 10, 18, 19, 21, 28]:
                plots.append(p)

            ax[i][j].set_yticks(y)
            ax[i][j].set_yticklabels(g)
            ax[i][j].invert_yaxis()  # labels read top-to-bottom
            ax[i][j].set_title(titles[ind], fontweight='bold', fontsize=14)
            ax[i][j].set_ylabel('Genes', fontweight='bold')
            ax[i][j].set_xlabel('Mutation rate (%)', fontweight='bold')

fig.legend(tuple(plots), ("Mitogenic", "DDR", "Splicing", "Ubiquitination", "Metabolic",
"Immunomodulators"), "lower right", prop={'size': 14})
plt.tight_layout()
plt.savefig("mut_rate.pdf")
```

Create a string "query" which contains all the different genes of pandas dataframe x, written in a

way that permits the search of only driver mutations when put on "Query by gene" search bar of cBioPortal.

```
cd ~\python programming\2

import pandas as pd

x = pd.read_csv("all_pathways_drivers_complete.csv")

query = ""
# Create a string "query" which contains all the different genes of x,
# written in a way that permits the search of only driver mutations when put
# on "Query by gene" search bar of cBioPortal.
for g in list(set(x.Gene)):
    query = query + g + ": DRIVER,"

query.strip()
query = query[:-1]
```

Program that renames all the 340 downloaded files of driver mutations, giving them the name of the gene where the driver mutations refer to. Then, it checks if all the files contain the same column names and with the same order, something critical for the right concatenation of the files in one file. Finally, it creates and saves a file containing all the driver mutations of all the genes for which there was such content at the respective file (204 out of the 340). In empty_files list, there are the file names of the genes that do not belong to the 204.

```
cd ~\python programming\2\driver mutations of our 340 cancer genes

import pandas as pd
import os
from pathlib import Path

# Sort files of the working directory (wd) by modification date (starting from
# the oldest one).
wd = 'C:\\Users\\Καλλιρρόη\\ΑΛΕΞΑΝΔΡΟΣ\\Paper E.I.E\\ΔΙΚΕΣ ΜΟΥ ΑΝΑΖΗΤΗΣΕΙΣ\\python programming\\2\\driver mutations of our 340 cancer genes'
paths = sorted(Path(wd).iterdir(), key=os.path.getmtime)

# There is a complete match at the elements order of fnames and gene_fnames.

fnames = []
for i in paths:
    fnames.append(str(i).split('\\')[-1])

gene_fnames = "TRIM24: DRIVER,DTX1: DRIVER,WRN: DRIVER,BRCA2: DRIVER,ATM: DRIVER,FANCG: DRIVER,RAD52: DRIVER,OMD: DRIVER,ZMYM3: DRIVER,JAK2: DRIVER,BAP1: DRIVER,EED: DRIVER,DDB2: DRIVER,RAD51: DRIVER,CYP17A1:
```

DRIVER,CD28: DRIVER,NBN: DRIVER,TNFAIP3: DRIVER,CYLD: DRIVER,CD276:
DRIVER,BRAF: DRIVER,KDM2B: DRIVER,NOTCH4: DRIVER,CBLB: DRIVER,POLQ:
DRIVER,CDKN1B: DRIVER,ATR: DRIVER,TP53BP1: DRIVER,PIK3C2G: DRIVER,PARP3:
DRIVER,GPC3: DRIVER,FANCE: DRIVER,ROS1: DRIVER,XPC: DRIVER,NTHL1:
DRIVER,CDK12: DRIVER,USP8: DRIVER,U2AF2: DRIVER,XRCC2: DRIVER,RPL10:
DRIVER,CHEK1: DRIVER,SF3B1: DRIVER,MED12: DRIVER,PPP1CB: DRIVER,NFE2L2:
DRIVER,BARD1: DRIVER,MAP3K1: DRIVER,PTPN13: DRIVER,ZNRF3: DRIVER,NUP214:
DRIVER,PDGFRA: DRIVER,RB1: DRIVER,NTRK2: DRIVER,SFRP2: DRIVER,RMI2:
DRIVER,NTRK3: DRIVER,SFRP1: DRIVER,SMAD4: DRIVER,KIT: DRIVER,MGA:
DRIVER,DDX3X: DRIVER,PIK3R3: DRIVER,IL2: DRIVER,ICOSLG: DRIVER,RAF1:
DRIVER,XPA: DRIVER,TLE1: DRIVER,MTAP: DRIVER,CBLC: DRIVER,ATIC:
DRIVER,CREBBP: DRIVER,PARP2: DRIVER,SOCS2: DRIVER,SPOP: DRIVER,KMT2A:
DRIVER,MET: DRIVER,ERCC2: DRIVER,FANCF: DRIVER,FGFR4: DRIVER,SDHD:
DRIVER,PER1: DRIVER,ETNK1: DRIVER,DCUN1D1: DRIVER,CCND2: DRIVER,AKT1:
DRIVER,DDX41: DRIVER,NRAS: DRIVER,CYP19A1: DRIVER,IDH1: DRIVER,TCEA1:
DRIVER,CDK4: DRIVER,NCOA2: DRIVER,GNAS: DRIVER,EXT1: DRIVER,SOCS3:
DRIVER,LRP6: DRIVER,RPTOR: DRIVER,SPRTN: DRIVER,SRSF2: DRIVER,POLE:
DRIVER,PML: DRIVER,NOTCH3: DRIVER,PIK3CG: DRIVER,DKK2: DRIVER,NUP93:
DRIVER,CCND3: DRIVER,PIK3C3: DRIVER,RANBP2: DRIVER,PPP2R1A:
DRIVER,CDKN2C: DRIVER,RNF43: DRIVER,RAD51B: DRIVER,GNB1: DRIVER,NT5C2:
DRIVER,DKK4: DRIVER,CDH1: DRIVER,TRIM27: DRIVER,MGAM: DRIVER,VHL:
DRIVER,INPP4A: DRIVER,FANCL: DRIVER,PMS2: DRIVER,CCNB1IP1: DRIVER,MDC1:
DRIVER,VTCN1: DRIVER,NCOR2: DRIVER,RELN: DRIVER,NF2: DRIVER,MDM4:
DRIVER,POLD1: DRIVER,NOTCH2: DRIVER,FYN: DRIVER,TRAF7: DRIVER,MUTYH:
DRIVER,TLE2: DRIVER,RAD51C: DRIVER,SDHC: DRIVER,FAT4: DRIVER,XIAP:
DRIVER,FURIN: DRIVER,TGFBR1: DRIVER,PMS1: DRIVER,PPP4R2: DRIVER,TP53:
DRIVER,RPL5: DRIVER,CLTC: DRIVER,CDKN1A: DRIVER,LATS1: DRIVER,GNAQ:
DRIVER,TRAF5: DRIVER,FLT3: DRIVER,NF1: DRIVER,CD274: DRIVER,APC:
DRIVER,IDH2: DRIVER,TLE3: DRIVER,VEGFA: DRIVER,PRKDC: DRIVER,YAP1:
DRIVER,GSK3B: DRIVER,BLM: DRIVER,NUP98: DRIVER,AXIN1: DRIVER,CD70:
DRIVER,TGFBR2: DRIVER,FBXO31: DRIVER,SDHB: DRIVER,U2AF1: DRIVER,SFRP4:
DRIVER,CUL4A: DRIVER,SMAD2: DRIVER,CDKN2B: DRIVER,PALB2: DRIVER,CCND1:
DRIVER,FUS: DRIVER,PSIP1: DRIVER,ACSL6: DRIVER,MSH3: DRIVER,DKK3:
DRIVER,CRBN: DRIVER,RECQL4: DRIVER,YWHAE: DRIVER,ALB: DRIVER,AXIN2:
DRIVER,HLA-C: DRIVER,DKK1: DRIVER,QKI: DRIVER,ACSL3: DRIVER,RPL22:
DRIVER,AKT2: DRIVER,RASA1: DRIVER,FAT1: DRIVER,GNA11: DRIVER,TCF7L2:
DRIVER,RAD54L: DRIVER,SPEN: DRIVER,RTEL1: DRIVER,ERCC3: DRIVER,PDCD1:
DRIVER,AMER1: DRIVER,EP300: DRIVER,ARNT: DRIVER,PIK3C2B: DRIVER,FGFR3:
DRIVER,FGFR2: DRIVER,TSC2: DRIVER,HEY1: DRIVER,MSH2: DRIVER,CUL3:
DRIVER,PIK3R1: DRIVER,PPARG: DRIVER,FANCC: DRIVER,KRAS: DRIVER,MAD2L2:
DRIVER,KMT2B: DRIVER,KDSR: DRIVER,TRIM33: DRIVER,PRKAR1A: DRIVER,E2F3:
DRIVER,CTNNB1: DRIVER,IL10: DRIVER,TSC1: DRIVER,IGF1R: DRIVER,CBL:
DRIVER,MTOR: DRIVER,WIF1: DRIVER,ALK: DRIVER,RHEB: DRIVER,ATRX:
DRIVER,CDKN2A: DRIVER,MDM2: DRIVER,RAD50: DRIVER,INPP4B: DRIVER,PC:
DRIVER,INPP5D: DRIVER,PIK3CA: DRIVER,FGFR1: DRIVER,HRAS: DRIVER,FH:
DRIVER,ERCC4: DRIVER,RAC1: DRIVER,FANCD2: DRIVER,CHEK2: DRIVER,PCBP1:
DRIVER,RICTOR: DRIVER,GPHN: DRIVER,SDC4: DRIVER,ALOX12B: DRIVER,RBM10:
DRIVER,EGFR: DRIVER,KEAP1: DRIVER,SDHA: DRIVER,PTEN: DRIVER,INPPL1:
DRIVER,UBR5: DRIVER,BRCA1: DRIVER,KDM5A: DRIVER,AKT3: DRIVER,PDK1:
DRIVER,MYC: DRIVER,FBXW7: DRIVER,ERBB2: DRIVER,FANCA: DRIVER,MSH6:
DRIVER,CDK8: DRIVER,STK11: DRIVER,NADK: DRIVER,PDCD1LG2: DRIVER,GMPS:
DRIVER,RET: DRIVER,ERCC5: DRIVER,MYCN: DRIVER,BIRC3: DRIVER,HLA-A:

```
DRIVER,WWTR1: DRIVER,AURKA: DRIVER,TPR: DRIVER,LRP5: DRIVER,PIK3R2:
DRIVER,ERRFI1: DRIVER,CPS1: DRIVER,RAD51D: DRIVER,TNFRSF14: DRIVER,MAX:
DRIVER,ZRSR2: DRIVER,FBXO11: DRIVER,TBL1XR1: DRIVER,PTPN11: DRIVER,SLX4:
DRIVER,PRKACA: DRIVER,NOTCH1: DRIVER,HLA-B: DRIVER,MAPK1: DRIVER,CAD:
DRIVER,HSD3B1: DRIVER,TRAF2: DRIVER,PIK3CB: DRIVER,CACNA1D:
DRIVER,CTLA4: DRIVER,MAP2K1: DRIVER,RXRA: DRIVER,ERBB3: DRIVER,SMAD3:
DRIVER,PARP1: DRIVER,TRAF3: DRIVER,NCOA1: DRIVER,DDX5: DRIVER,SOCS1:
DRIVER,EXT2: DRIVER,PIK3CD: DRIVER,NTRK1: DRIVER,MAP2K2: DRIVER,PRKD1:
DRIVER,USP6: DRIVER,BTLA: DRIVER,FUBP1: DRIVER,CDK6: DRIVER,ARAF:
DRIVER,MYCL: DRIVER,LATS2: DRIVER,RECQL: DRIVER,SOS1: DRIVER,CCNE1:
DRIVER,SMARCA4: DRIVER,ABL1: DRIVER,CARM1: DRIVER,ACVR2A: DRIVER,PRF1:
DRIVER,MLH1: DRIVER,RNF213: DRIVER,SLC45A3: DRIVER,BRIP1: DRIVER,CD36:
DRIVER,PRSS1: DRIVER,MIB1: DRIVER,NCOR1: DRIVER,TLE4: DRIVER,NCOA3:
DRIVER,BABAM1: DRIVER,ERBB4: DRIVER".replace(": DRIVER", ".tsv").split(",")

for n in range(len(fnames)):
    ind = os.listdir().index(fnames[n])
    os.rename(os.listdir()[ind], gene_fnames[n])

empty_files = []
c = -1
dfs = []
for file in os.listdir():
    c += 1
    x = pd.read_csv(file, sep="\t")
    if len(x) > 0:
        # There is at least one file (HLA-A.tsv) without column "Annotation".
        # If we don't fix this, the dataframes concatenation will not be correct.
        if x.columns[4] != "Annotation":
            x.insert(loc=4, column="Annotation", value="")

        # Add column "Gene" as the first column of each pd.DataFrame.
        x.insert(loc=0, column='Gene', value=os.listdir()[c].replace(".tsv", ""))
        dfs.append(x)
    else:
        # Keep the names of the files that lack entries.
        empty_files.append(file)
        continue

# Check if all DataFrames in dfs have the same column names and in the same
# order. This is very important for an appropriate concatenation.
same_columns = 0
for df in dfs:
    if len(dfs[0].columns == df.columns) == len(df.columns):
        same_columns += 1
same_columns == len(dfs)

# Concatenate pd.DataFrames
final_df = pd.DataFrame()
for df in dfs:
    final_df = pd.concat([final_df, df])
final_df.reset_index().drop(columns="index").to_csv("driver_mutations_for_204_cancer_genes.csv
```

")

Program that extends all_pathways_drivers_complete.csv file by a column called "Driver Mutation rate (%)" that contains the percentage (keeping 2 decimal places) of TCGA PanCancer Atlas projects samples that have driver mutations in the corresponding cancer gene.

```
cd ~\python programming\2

import pandas as pd

genes = pd.read_csv("all_pathways_drivers_complete.csv")
genes["Driver Mutation rate (%)"] = 0
for gene in list(set(final_df.Gene)):
    # The TCGA PanCancer Atlas Studies used for this analysis contain 10439
    # samples profiled for their mutations.
    percent_driver = len(set(final_df[final_df.Gene == gene]["Sample ID"])) / 10439 * 100

    # Format to two decimal places
    formatted_string = "{:.2f}".format(percent_driver)
    percent_driver = float(formatted_string)

    # Find the rows where Gene equals to gene and put the percent_driver in
    # "Driver Mutation rate (%)" column.
    genes.loc[genes.Gene == gene, "Driver Mutation rate (%)"] = percent_driver

genes.to_csv("all_pathways_drivers_more_complete.csv")
```

Program that creates a figure containing 27 horizontal bar graphs, each showing the driver mutational rate of the cancer genes of a different pathway.

```
cd ~\python programming\2

import pandas as pd
import matplotlib.pyplot as plt
import numpy as np

genes = pd.read_csv("all_pathways_drivers_more_complete.csv")
genes = genes.drop(columns=["Unnamed: 0", "Unnamed: 0.1"])

# How many out of the 31 pathways are represented in genes Dataframe ?
len(set(genes.Pathway))
# None of the genes of DR and NP pathways' gene lists are characterized as
# cancer genes by oncoKB database. Thus, from the following pathways list,
# these two pathways were excluded.

pathways = ["RTK_RAS", "cell_cycle", "p53", "PI3K_Akt", "b_catenin_Wnt", "Notch", "Hippo",
```

```
"Myc", "TGFb", "Nrf2", "BER", "NER", "MMR", "FA", "HDR", "NHEJ", "Others_DDR",
"Spliceosome", "Ubq", "Dubq", "Amino_acid", "Carbohydrates", "Energy", "Lipid", "Nucleotide",
"TCA_cycle", "Immunomodulators"]
titles = ["RTK/RAS", "Cell cycle", "p53", "PI3K Akt", "b catenin/Wnt", "Notch", "Hippo", "Myc",
"TGFb", "Nrf2", "BER", "NER", "MMR", "FA", "HDR", "NHEJ", "Other DDR", "Splicing",
"Ubiquitination", "Deubiquitination", "Amino acids", "Carbohydrates", "Energy", "Lipids",
"Nucleotides", "TCA cycle", "Immunomodulators"]

fig, ax = plt.subplots(3, 10)
fig.set_size_inches(30, 40)
plt.subplots_adjust(wspace=3.5)
for i in range(7, 10):
    ax[2, i].remove()

plots = []

colors = ["r", "r", "r", "r", "r", "r", "r", "r", "r", "r", "b", "b", "b", "b", "b", "b", "b", "m", "g", "g",
"c", "c", "c", "c", "c", "c", "y"]
ind = -1
for i in range(3):
    for j in range(10):
        if i == 2 and j in [7, 8, 9]:
            continue
        else:
            ind += 1
            z = genes[genes.Pathway == pathways[ind]].sort_values(by="Driver Mutation rate (%)",
ascending=False)
            g = list(z.Gene)
            y = np.arange(len(g))
            x = np.array(z["Driver Mutation rate (%)"])
            p = ax[i][j].barh(y, x, align='center', color=colors[ind])

            # Add only the first plot of each category to plots list
            if ind in [0, 10, 17, 18, 20, 26]:
                plots.append(p)

            ax[i][j].set_yticks(y)
            ax[i][j].set_yticklabels(g, fontsize=10)
            ax[i][j].invert_yaxis()  # labels read top-to-bottom
            ax[i][j].set_title(titles[ind], fontweight='bold', fontsize=14)
            ax[i][j].set_ylabel('Genes', fontweight='bold')
            ax[i][j].set_xlabel('Driver Mutation rate (%)', fontweight='bold')

fig.legend(tuple(plots), ("Mitogenic", "DDR", "Splicing", "Ubiquitination", "Metabolic",
"Immunomodulators"), "lower right", prop={'size': 14})
plt.tight_layout()
plt.savefig("driver_mut_rate.pdf")
```

Program that creates a list of tuples with each tuple containing a pathway name and the total number of driver mutations found in its genes over all the TCGA PanCancer Atlas projects samples

profiled for their mutations.

```
cd ~\python programming\2\driver mutations of our 340 cancer genes

import pandas as pd

final_df = pd.read_csv("driver_mutations_for_204_cancer_genes.csv")

cd ~\python programming\2

genes = pd.read_csv("all_pathways_drivers_more_complete.csv")
genes = genes.drop(columns=["Unnamed: 0", "Unnamed: 0.1"])

driver_mut_path = []
for path in list(set(genes.Pathway)):
    num_driver_mut = 0
    for g in list(set(genes[genes.Pathway == path]["Gene"])):
        num_driver_mut += len(final_df[final_df.Gene == g])
    driver_mut_path.append((path, num_driver_mut))

driver_mut_path = sorted(driver_mut_path, key = lambda x: x[1], reverse=True)
```

Program that calculates the percentage of samples with driver mutations for 27 cancer related pathways. The calculation refers to all the TCGA PanCancer Atlas projects samples profiled for their mutations.

```
cd ~\python programming\2\driver mutations of our 340 cancer genes

import pandas as pd

final_df = pd.read_csv("driver_mutations_for_204_cancer_genes.csv")

cd ~\python programming\2

genes = pd.read_csv("all_pathways_drivers_more_complete.csv")
genes = genes.drop(columns=["Unnamed: 0", "Unnamed: 0.1"])
genes = genes[genes["Driver Mutation rate (%)"] > 0]

paths_driver_mut = pd.DataFrame(columns=["Pathway", "Samples with driver mutations(%)"])
c = 0
for path in list(set(genes.Pathway)):
    c += 1
    samples = []
    for g in list(set(genes[genes.Pathway == path]["Gene"])):
        for s in list(final_df[final_df.Gene == g]["Sample ID"]):
            samples.append(s)

    # The TCGA PanCancer Atlas Studies used for this analysis contain 10439
    # samples profiled for their mutations.
    percent = len(set(samples)) / 10439 * 100
```

```
    # Format to two decimal places
    formatted_string = "{:.2f}".format(percent)
    percent = float(formatted_string)

    paths_driver_mut.loc[c] = path, percent
paths_driver_mut = paths_driver_mut.sort_values(by="Samples with driver mutations(%)",
ascending=False).reset_index().drop(columns="index")
paths_driver_mut.to_csv("paths_driver_mut_rate.csv")
```

Program that adds the cancer stage for each sample in "driver_mutations_for_204_cancer_genes.csv" file. Then, it creates a new file "driver_mutations_for_204_cancer_genes_and_stage.csv" with only these entries that correspond to samples for which their cancer stage is available.

```
cd ~\python programming\2\driver mutations of our 340 cancer genes

import pandas as pd

muts = pd.read_csv("driver_mutations_for_204_cancer_genes.csv").drop(columns="Unnamed: 0")
muts.insert(loc=4, column="Stage", value=0)

ids = list(set(muts["Sample ID"]))

cd ~\python programming\2

clinical = pd.read_csv("clinical_data.tsv", sep="\t")

for ID in ids:
    ind = clinical[clinical["Sample ID"] == ID].index[0]
    stage = clinical.iloc[ind]["Neoplasm Disease Stage American Joint Committee on Cancer Code"]
    muts.loc[muts["Sample ID"] == ID, "Stage"] = stage

# nan values (not a number) are float numbers. So, you can find if an item "x"
# is a nan by asking for its type --> type(x) == float. If the only float
# items are the nan items, then you can identify them this way.

dropped_ind = []
samples = []
cancer_types = []
for i in muts.index:
    if type(muts.iloc[i].Stage) == float:
        dropped_ind.append(i)
        samples.append(muts.iloc[i]["Sample ID"])
        cancer_types.append(muts.iloc[i]["Cancer Type"])
samples = set(samples)
cancer_types = set(cancer_types)
muts.drop(dropped_ind).reset_index().drop(columns="index").to_csv("driver_mutations_samples_
with_stage_available.csv")

# len(samples) equals to 2951 and cancer_types equals to 46.
# This means that for the 2951 out of 8276 samples the cancer stage is not
```

available. Also, these 2951 samples belong to 46 out of 67 cancer types. The
new file "driver_mutations_samples_with_stage_available.csv" contains only
entries with available cancer stage.

Program that isolates only the driver mutational data of the samples with known stage and creates a table containing the type of cancer, the total number of unique samples suffering from each type of cancer and the total number of samples of each stage.

```
import pandas as pd

cd ~\python programming\2

muts = pd.read_csv("driver_mutations_samples_with_stage_available.csv")

# len(set(muts["Cancer Type"])) equals to 40. So, from the 67 cancer types,
# the stage of the disease is available for patients belonging to the 40 of them.

remaining = pd.DataFrame(columns=["Cancer Type", "Number of samples"])

# The following stages are all the different stages present on "remaining"
# DataFrame. We can display them using the command set(muts.Stage).
stages = ['STAGE 0',
 'STAGE I',
 'STAGE I/II (NOS)',
 'STAGE IA',
 'STAGE IB',
 'STAGE II',
 'STAGE IIA',
 'STAGE IIB',
 'STAGE IIC',
 'STAGE III',
 'STAGE IIIA',
 'STAGE IIIB',
 'STAGE IIIC',
 'STAGE IS',
 'STAGE IV',
 'STAGE IVA',
 'STAGE IVB',
 'STAGE IVC',
 'STAGE X']

for st in stages:
    remaining[st] = 0

n = 0
c = 0
for ct in list(set(muts["Cancer Type"])):
    n += len(set(muts[muts["Cancer Type"] == ct]["Sample ID"]))
    x = muts[muts["Cancer Type"] == ct]
    remaining.loc[c, remaining.columns[:2]] = ct, len(set(x["Sample ID"]))
```

```
    for st in remaining.columns[2:]:
        remaining.loc[c, st] = len(set(x[x.Stage == st]["Sample ID"]))
    c += 1
remaining.sort_values(by="Number of samples", ascending=False).reset_index().drop(columns="index").to_csv("remaining_samples_stage_available.csv")

# Check if the sum of unique samples is right.
n == len(set(muts["Sample ID"]))
```

Program that modifies the file remaining_samples_stage_available.csv in order to include columns with the total number of samples of each stage.

```
cd ~\python programming\2

import pandas as pd

nums = pd.read_csv("remaining_samples_stage_available.csv", index_col=0)

stage_I = ["STAGE I", "STAGE IA", "STAGE IB"]
stage_II = ["STAGE II", "STAGE IIA", "STAGE IIB", "STAGE IIC"]
stage_III = ["STAGE III", "STAGE IIIA", "STAGE IIIB", "STAGE IIIC"]
stage_IV = ["STAGE IV", "STAGE IVA", "STAGE IVB", "STAGE IVC"]

stages = [stage_I, stage_II, stage_III, stage_IV]

pools = ["Pool STAGE I", "Pool STAGE II", "Pool STAGE III", "Pool STAGE IV"]

c = 0
for i in range(2, 6):
    nums.insert(loc=i, column=pools[c], value="")
    c += 1

pn = -1
for stage in stages:
    pn += 1
    for z in range(len(nums)):
        nums.loc[z, pools[pn]] = sum([nums.iloc[z][k] for k in stage])

nums.to_csv("remaining_samples_stage_available_pool.csv")
```

Program that clusters the substages (e.g. “STAGE IA” and “STAGE IB”) into the corresponding stages (e.g. I) and adds a new column “General Stage” that contains this information. This, is being done only for stages I – IV, not for the rest categories (e.g. “STAGE IS”, “STAGE X” etc). Also, it creates a new column “Change” that contains the mutated gene name and the protein change in a single column.

```
cd ~\python programming\2
```

```
import pandas as pd

muts = pd.read_csv("driver_mutations_samples_with_stage_available.csv", index_col=0)

stage_I = ["STAGE I", "STAGE IA", "STAGE IB"]
stage_II = ["STAGE II", "STAGE IIA", "STAGE IIB", "STAGE IIC"]
stage_III = ["STAGE III", "STAGE IIIA", "STAGE IIIB", "STAGE IIIC"]
stage_IV = ["STAGE IV", "STAGE IVA", "STAGE IVB", "STAGE IVC"]

stages = [("I", stage_I), ("II", stage_II), ("III", stage_III), ("IV", stage_IV)]

muts.insert(loc=5, column="General Stage", value="")
muts.insert(loc=7, column="Change", value="")

for i in range(len(muts)):

    x = muts.iloc[i]["Stage"]
    for z in range(len(stages)):
        if x in stages[z][1]:
            muts.loc[i, "General Stage"] = stages[z][0]

    ch = muts.iloc[i]["Gene"] + ", " + muts.iloc[i]["Protein Change"]
    muts.loc[i, "Change"] = ch

muts.to_csv("driver_mutations_samples_with_stage_available_fixed.csv")
```

Find mutations per stage and cancer type (alteration level analysis)

```
cd ~\python programming\2

import pandas as pd

nss = pd.read_csv("remaining_samples_stage_available_pool.csv", index_col=0)
muts = pd.read_csv("driver_mutations_samples_with_stage_available_fixed.csv", index_col=0)

# In our analysis, we exclude the following stages :
# "STAGE 0" and "STAGE IS" due to lack of samples in the cancer types of interest,
# "STAGE I/II (NOS)" and "STAGE X" due to the unclear nature of these stages.

stage_I = ["STAGE I", "STAGE IA", "STAGE IB"]
stage_II = ["STAGE II", "STAGE IIA", "STAGE IIB", "STAGE IIC"]
stage_III = ["STAGE III", "STAGE IIIA", "STAGE IIIB", "STAGE IIIC"]
stage_IV = ["STAGE IV", "STAGE IVA", "STAGE IVB", "STAGE IVC"]

cancer_types = ["Breast Invasive Ductal Carcinoma", "Lung Adenocarcinoma", "Lung Squamous
Cell Carcinoma", "Papillary Thyroid Cancer", "Head and Neck Squamous Cell Carcinoma",
"Cutaneous Melanoma", "Bladder Urothelial Carcinoma", "Colon Adenocarcinoma",
"Hepatocellular Carcinoma", "Renal Clear Cell Carcinoma", "Pancreatic Adenocarcinoma", "Breast
```

```
Invasive Lobular Carcinoma", "Stomach Adenocarcinoma", "Rectal Adenocarcinoma", "Papillary Renal Cell Carcinoma"]

dfs = []
c = -1
for can in cancer_types:
    mut_st = pd.DataFrame(columns=["Cancer Type", "Pool STAGE I", "Pool STAGE II", "Pool STAGE III", "Pool STAGE IV", "Change", "STAGE I", "STAGE I (%)", "STAGE II", "STAGE II (%)", "STAGE III", "STAGE III (%)", "STAGE IV", "STAGE IV (%)"])
    x = muts[muts["Cancer Type"] == can]
    ind = nss[nss["Cancer Type"] == can].index[0]
    a = nss.iloc[ind]["Pool STAGE I"]
    b = nss.iloc[ind]["Pool STAGE II"]
    d = nss.iloc[ind]["Pool STAGE III"]
    e = nss.iloc[ind]["Pool STAGE IV"]
    new_entry = [can, a, b, d, e]

    for change in list(set(x.Change)):
        c += 1
        z = x[x.Change == change]
        appearences = [change]
        for st in ["I", "II", "III", "IV"]:
            appears = len(z[z["General Stage"] == st])
            # Some stages are not represented by any samples so at these
            # cases the denominator equals to zero, which is mathematically wrong.
            # Format to two decimal places.
            try:
                perc = appears / len(x[x["General Stage"] == st]) * 100
                formatted_string = "{:.2f}".format(perc)
                perc = float(formatted_string)
            except ZeroDivisionError:
                perc = "not applicable"
            # The binary value of "existence" variable represents the
            # existence (=1) or absence (=0) of a given change in a given stage.
            if appears > 0:
                existence = 1
            elif appears == 0:
                existence = 0
            appearences.append(existence)
            appearences.append(perc)

        values = []
        for item in [new_entry, appearences]:
            for value in item:
                values.append(value)

        mut_st.loc[c] = values
    dfs.append(mut_st)

mut_st = pd.concat(dfs).reset_index().drop(columns="index")
mut_st.to_csv("mutations_per_cancer_type_and_stage.csv")
```

```
# Add a column showing all the stages each change occurs in.
eps = []
for i in range(len(mut_st)):
    sts = []
    for col in ['STAGE I', 'STAGE II', 'STAGE III', 'STAGE IV']:
        if mut_st.iloc[i][col] == 1:
            sts.append(col.split()[1])
    eps.append(", ".join(sts))
# "COS" stands for "Change Occurence per Stage" and each character of it
# represents the existence (=1) or absence(=0) of a given change (e.g. BRAF, V600E)
# at the samples examined for a given cancer type (e.g. 0101 means that the change)
# was observed only at stages II and IV and not at stages I and III)
mut_st["COS"] = eps
mut_st.to_csv("mutations_per_cancer_type_and_stage.csv")
```

Find mutations per stage and cancer type (gene level analysis)

```
cd ~\python programming\2

import pandas as pd

nss = pd.read_csv("remaining_samples_stage_available_pool.csv", index_col=0)
muts = pd.read_csv("driver_mutations_samples_with_stage_available_fixed.csv", index_col=0)

cancer_types = ["Breast Invasive Ductal Carcinoma", "Lung Adenocarcinoma", "Lung Squamous
Cell Carcinoma", "Papillary Thyroid Cancer", "Head and Neck Squamous Cell Carcinoma",
"Cutaneous Melanoma", "Bladder Urothelial Carcinoma", "Colon Adenocarcinoma",
"Hepatocellular Carcinoma", "Renal Clear Cell Carcinoma", "Pancreatic Adenocarcinoma", "Breast
Invasive Lobular Carcinoma", "Stomach Adenocarcinoma", "Rectal Adenocarcinoma", "Papillary
Renal Cell Carcinoma"]

dfs = []
c = -1
for can in cancer_types:
    mut_st = pd.DataFrame(columns=["Cancer Type", "Pool STAGE I", "Pool STAGE II", "Pool
STAGE III", "Pool STAGE IV", "Altered Gene", "STAGE I", "STAGE I (%)", "STAGE II",
"STAGE II (%)", "STAGE III", "STAGE III (%)", "STAGE IV", "STAGE IV (%)", "COS"])

    # "COS" stands for "Change Occurence per Stage" and represents all the stages
    # of a particular cancer type, at which at least one sample identified with
    # either a specific driver alteration (for alteration level analysis) or any
    # driver mutation at a specific gene (for gene level analysis).
```

```
x = muts[muts["Cancer Type"] == can]
ind = nss[nss["Cancer Type"] == can].index[0]
a = nss.iloc[ind]["Pool STAGE I"]
b = nss.iloc[ind]["Pool STAGE II"]
d = nss.iloc[ind]["Pool STAGE III"]
e = nss.iloc[ind]["Pool STAGE IV"]
new_entry = [can, a, b, d, e]

for gene in list(set(x.Gene)):
    c += 1
    z = x[x.Gene == gene]
    appearences = [gene]

    # Find if the gene is altered or not at each stage and the corresponding
    # stage-specific percentage (%).
    for st in ["I", "II", "III", "IV"]:
        appears = len(z[z["General Stage"] == st])
        # Some stages are not represented by any samples so at these
        # cases the denominator equals to zero, which is mathematically wrong.
        # Format "perc" to two decimal places.
        try:
            perc = appears / len(x[x["General Stage"] == st]) * 100
            formatted_string = "{:.2f}".format(perc)
            perc = float(formatted_string)
        except ZeroDivisionError:
            perc = "not applicable"
        # The binary value of "existence" variable represents the
        # existence (=1) or absence (=0) of a given change in a given stage.
        if appears > 0:
            existence = 1
        elif appears == 0:
            existence = 0
        appearences.append(existence)
        appearences.append(perc)

    # Collect the stages where the gene is altered in at least one sample.
    sts = []
    for binar in [(1, 'STAGE I'), (3, 'STAGE II'), (5, 'STAGE III'), (7, 'STAGE IV')]:
        if appearences[binar[0]] == 1:
            sts.append(binar[1].split()[1])
    sts = ", ".join(sts)
    appearences.append(sts)

    # Collect all the values you want to include in the row that refers
    # to this gene and for the particular cancer type.
    values = []
    for item in [new_entry, appearences]:
        for value in item:
            values.append(value)

    mut_st.loc[c] = values
```

```
    dfs.append(mut_st)

mut_st = pd.concat(dfs).reset_index().drop(columns="index")
mut_st.to_csv("mutated_genes_per_cancer_type_and_stage.csv")
```

Sort the rows by the occurence (%) of the alteration (protein alteration or altered gene) at the stage with the highest representation in each cancer type.

```
cd ~\python programming\2

import pandas as pd

file_names = ["mutations_per_cancer_type_and_stage.csv",
"mutated_genes_per_cancer_type_and_stage.csv"]
cancer_types = ["Breast Invasive Ductal Carcinoma", "Lung Adenocarcinoma", "Lung Squamous
Cell Carcinoma", "Papillary Thyroid Cancer", "Head and Neck Squamous Cell Carcinoma",
"Cutaneous Melanoma", "Bladder Urothelial Carcinoma", "Colon Adenocarcinoma",
"Hepatocellular Carcinoma", "Renal Clear Cell Carcinoma", "Pancreatic Adenocarcinoma", "Breast
Invasive Lobular Carcinoma", "Stomach Adenocarcinoma", "Rectal Adenocarcinoma", "Papillary
Renal Cell Carcinoma"]

for fname in file_names:
    mut_st = pd.read_csv(fname, index_col=0)
    dfs = []
    for can in cancer_types:
        x = mut_st[mut_st["Cancer Type"] == can]
        # Sort the rows by the occurence (%) of the alteration (protein
        # alteration or altered gene) at the stage with the highest
        # representation in each cancer type.
        n_samples = []
        for col in mut_st.columns[1:5]:
            # We use the zero but it doesn't matter because all the rows
            # contain the same numbers at these columns for a particular
            # cancer type.
            n_samples.append(x.iloc[0][col])
        ind = n_samples.index(max(n_samples))
        # Get rid of the string "Pool " of column name and form the name
        # of the column we want to sort the rows by.
        sort_col = mut_st.columns[1:5][ind][5:] + " (%)"
        x = x.sort_values(by=sort_col, ascending=False)
        dfs.append(x)
    final_df = pd.concat(dfs).reset_index().drop(columns="index")
    final_df.to_csv(fname[:-4] + "_sorted" + ".csv")
```

Find mutations per stage and cancer type (gene level analysis) FOR ALL CANCER TYPES (includes all 40 types). The results for each cancer type have been sorted by the values of “COS”

column.

```
cd ~\python programming\2

import pandas as pd

nss = pd.read_csv("remaining_samples_stage_available_pool.csv", index_col=0)
muts = pd.read_csv("driver_mutations_samples_with_stage_available_fixed.csv", index_col=0)

# In our analysis, we exclude the following stages :
# "STAGE 0" and "STAGE IS" due to lack of samples in the cancer types of interest,
# "STAGE I/II (NOS)" and "STAGE X" due to the unclear nature of these stages.

stage_I = ["STAGE I", "STAGE IA", "STAGE IB"]
stage_II = ["STAGE II", "STAGE IIA", "STAGE IIB", "STAGE IIC"]
stage_III = ["STAGE III", "STAGE IIIA", "STAGE IIIB", "STAGE IIIC"]
stage_IV = ["STAGE IV", "STAGE IVA", "STAGE IVB", "STAGE IVC"]

cancer_types = list(nss["Cancer Type"])

dfs = []
c = -1
for can in cancer_types:
    mut_st = pd.DataFrame(columns=["Cancer Type", "Pool STAGE I", "Pool STAGE II", "Pool
STAGE III", "Pool STAGE IV", "Altered Gene", "STAGE I", "STAGE I (%)", "STAGE II",
"STAGE II (%)", "STAGE III", "STAGE III (%)", "STAGE IV", "STAGE IV (%)", "COS"])

    # "COS" stands for "Change Occurence per Stage" and represents all the stages
    # of a particular cancer type, at which at least one sample identified with
    # either a specific driver alteration (for alteration level analysis) or any
    # driver mutation at a specific gene (for gene level analysis).

    x = muts[muts["Cancer Type"] == can]
    ind = nss[nss["Cancer Type"] == can].index[0]
    a = nss.iloc[ind]["Pool STAGE I"]
    b = nss.iloc[ind]["Pool STAGE II"]
    d = nss.iloc[ind]["Pool STAGE III"]
    e = nss.iloc[ind]["Pool STAGE IV"]
    new_entry = [can, a, b, d, e]

    for gene in list(set(x.Gene)):
        c += 1
        z = x[x.Gene == gene]
        appearences = [gene]

        # Find if the gene is altered or not at each stage and the corresponding
        # stage-specific percentage (%).
        for st in ["I", "II", "III", "IV"]:
            # "appears" is the number of samples of a particular cancer type and
            # a particular stage where a particular gene is mutated.
            # "n_samples_st_can" is the number of samples of a specific cancer
            # type that have a disease of a particular stage.
```

```
            appears = len(set(z[z["General Stage"] == st]["Sample ID"]))
            n_samples_st_can = len(set(x[x["General Stage"] == st]["Sample ID"]))
            # Some stages are not represented by any samples so at these
            # cases the denominator equals to zero, which is mathematically wrong.
            # Format "perc" to two decimal places.
            try:
                perc = appears / n_samples_st_can * 100
                formatted_string = "{:.2f}".format(perc)
                perc = float(formatted_string)
            except ZeroDivisionError:
                perc = "not applicable"
            # The binary value of "existence" variable represents the
            # existence (=1) or absence (=0) of a given change in a given stage.
            if appears > 0:
                existence = 1
            elif appears == 0:
                existence = 0
            appearences.append(existence)
            appearences.append(perc)

        # Collect the stages where the gene is altered in at least one sample.
        sts = []
        for binar in [(1, 'STAGE I'), (3, 'STAGE II'), (5, 'STAGE III'), (7, 'STAGE IV')]:
            if appearences[binar[0]] == 1:
                sts.append(binar[1].split()[1])
        sts = ", ".join(sts)
        appearences.append(sts)

        # Collect all the values you want to include in the row that refers
        # to this gene and for the particular cancer type.
        values = []
        for item in [new_entry, appearences]:
            for value in item:
                values.append(value)

        mut_st.loc[c] = values

    dfs.append(mut_st.sort_values(by="COS"))


mut_st = pd.concat(dfs).reset_index().drop(columns="index")
mut_st.to_csv("CORRECTED_mutated_genes_per_cancer_type_and_stage_all_cancers.csv")
```

Find mutations per stage and cancer type (alteration level analysis) FOR ALL CANCER TYPES (includes all 40 types). The results for each cancer type have been sorted by the values of “COS” column.

```
nss = pd.read_csv("remaining_samples_stage_available_pool.csv", index_col=0)
muts = pd.read_csv("driver_mutations_samples_with_stage_available_fixed.csv", index_col=0)

# In our analysis, we exclude the following stages :
# "STAGE 0" and "STAGE IS" due to lack of samples in the cancer types of interest,
```

```
# "STAGE I/II (NOS)" and "STAGE X" due to the unclear nature of these stages.

stage_I = ["STAGE I", "STAGE IA", "STAGE IB"]
stage_II = ["STAGE II", "STAGE IIA", "STAGE IIB", "STAGE IIC"]
stage_III = ["STAGE III", "STAGE IIIA", "STAGE IIIB", "STAGE IIIC"]
stage_IV = ["STAGE IV", "STAGE IVA", "STAGE IVB", "STAGE IVC"]

cancer_types = list(nss["Cancer Type"])

dfs = []
c = -1
for can in cancer_types:
    mut_st = pd.DataFrame(columns=["Cancer Type", "Pool STAGE I", "Pool STAGE II", "Pool
STAGE III", "Pool STAGE IV", "Change", "STAGE I", "STAGE I (%)", "STAGE II", "STAGE II
(%)", "STAGE III", "STAGE III (%)", "STAGE IV", "STAGE IV (%)", "COS"])
    x = muts[muts["Cancer Type"] == can]
    ind = nss[nss["Cancer Type"] == can].index[0]
    a = nss.iloc[ind]["Pool STAGE I"]
    b = nss.iloc[ind]["Pool STAGE II"]
    d = nss.iloc[ind]["Pool STAGE III"]
    e = nss.iloc[ind]["Pool STAGE IV"]
    new_entry = [can, a, b, d, e]

    for change in list(set(x.Change)):
        c += 1
        z = x[x.Change == change]
        appearences = [change]
        occ = []
        for st in ["I", "II", "III", "IV"]:
            # "appears" is the number of samples of a particular cancer type and
            # a particular stage where a particular mutation is found.
            # "n_samples_st_can" is the number of samples of a specific cancer
            # type that have a disease of a particular stage.
            appears = len(set(z[z["General Stage"] == st]["Sample ID"]))
            n_samples_st_can = len(set(x[x["General Stage"] == st]["Sample ID"]))
            # Some stages are not represented by any samples so at these
            # cases the denominator equals to zero, which is mathematically wrong.
            # Format "perc" to two decimal places.
            try:
                perc = appears / n_samples_st_can * 100
                formatted_string = "{:.2f}".format(perc)
                perc = float(formatted_string)
            except ZeroDivisionError:
                perc = "not applicable"
            # The binary value of "existence" variable represents the
            # existence (=1) or absence (=0) of a given change in a given stage.
            if appears > 0:
                existence = 1
                occ.append(st)
            elif appears == 0:
                existence = 0
            appearences.append(existence)
```

```
        appearences.append(perc)
     # Create a string whith all stages where the change occurs in and
     # add it into appearences.
     appearences.append(", ".join(occ))
     values = []
     for item in [new_entry, appearences]:
        for value in item:
           values.append(value)

     mut_st.loc[c] = values
  dfs.append(mut_st.sort_values(by="COS"))

mut_st = pd.concat(dfs).reset_index().drop(columns="index")
# "COS" stands for "Change Occurence per Stage" and each character of it
# represents the existence (=1) or absence(=0) of a given change (e.g. BRAF, V600E)
# at the samples examined for a given cancer type (e.g. 0101 means that the change)
# was observed only at stages II and IV and not at stages I and III)
mut_st.to_csv("CORRECTED_mutations_per_cancer_type_and_stage_all_cancers.csv")
```

Isolate only the rows of "mutated_genes_per_cancer_type_and_stage_all_cancers.csv" that refer to genes found mutated only in stage I of each cancer.

```
cd ~\python programming\2

import pandas as pd

muts = pd.read_csv("CORRECTED_mutated_genes_per_cancer_type_and_stage_all_cancers.csv",
index_col=0)
muts[muts.COS == "I"].drop(columns=["STAGE I", "STAGE II", "STAGE III", "STAGE
IV"]).to_csv("genes_mutated_only_in_stage_I_all_cancers.csv")
```

Program that organizes the information of gene driver alterations at pathway level and visualizes the data (cancer type – cancer stage- altered pathway).

```
cd ~\python programming\2

import pandas as pd
import numpy as np
import matplotlib.pyplot as plt

data = pd.read_csv("driver_mutations_samples_with_stage_available_fixed.csv", index_col=0)
```

```
pg = pd.read_csv("all_pathways_drivers_more_complete.csv", index_col=0)

cantypes =
pd.read_csv("CORRECTED_mutated_genes_per_cancer_type_and_stage_all_cancers.csv")

data = data.drop(columns=["Study", "Stage"]).drop(columns=data.columns[6:])
# Keep only the rows where "General Stage" belongs to the list ["I", "II", "III", "IV"]
no_stage_indices = []
for ind in data.index:
    if type(data.iloc[ind]["General Stage"]) == float:
        no_stage_indices.append(ind)
data = data.drop(no_stage_indices).reset_index().drop(columns="index")

data = data[["Cancer Type", "General Stage", "Sample ID", "Gene"]]
data["Pathway"] = None


# Match genes with the pathways they participate in.
for gene in list(pg["Gene"]):
    paths = ", ".join(list(pg[pg.Gene == gene]["Pathway"]))
    for i in list(data[data.Gene == gene].index):
        data.loc[i, "Pathway"] = paths

# With the following block of code we create a list of all our cancer types
# with a specific order : from the type with the most samples to this with the
# less. This happens due to the appropriate order in which registries are placed
# in cantypes.
cancer_types = []
for can in list(cantypes["Cancer Type"]):
    if can not in cancer_types:
        cancer_types.append(can)

# In patients with stage availiable, no driver mutation was found in TLS and
# Vitamin_cofactor pathways, so we exclude them from the next two variables:
pathways = ["RTK_RAS", "cell_cycle", "p53", "PI3K_Akt", "b_catenin_Wnt", "Notch", "Hippo",
"Myc", "TGFb", "Nrf2", "BER", "NER", "MMR", "FA", "HDR", "NHEJ", "Others_DDR",
"Spliceosome", "Ubq", "Dubq", "Amino_acid", "Carbohydrates", "Energy", "Lipid", "Nucleotide",
"TCA_cycle", "Immunomodulators"]
titles = ["RTK/RAS", "Cell cycle", "p53", "PI3K/Akt", "b catenin/Wnt", "Notch", "Hippo", "Myc",
"TGFb", "Nrf2", "BER", "NER", "MMR", "FA", "HDR", "NHEJ", "Other DDR", "Splicing",
"Ubiquitination", "Deubiquitination", "Amino acids", "Carbohydrates", "Energy", "Lipids",
"Nucleotides", "TCA cycle", "Immunomodulators"]

all_ds_and_titles = []
for cancer in cancer_types:
    x = data[data["Cancer Type"] == cancer].reset_index().drop(columns="index")
    cancer_d = dict()
    c = -1
    for path in pathways:
        c += 1
        indices = []
```

```
        for i in x.index:
            if path in x.iloc[i]["Pathway"]:
                indices.append(i)
        xp = x.iloc[indices]
        # "n_samples_can_path" is the number of samples of a particular cancer
        # type that harbor at least one driver mutation in genes of
        # a particular signaling pathway.
        n_samples_can_path = len(set(xp["Sample ID"]))
        if n_samples_can_path == 0:
            continue
        else:
            percentages_path = []
            for st in ["I", "II", "III", "IV"]:
                # "appears" is the number of samples of a particular cancer type
                # that harbor at least one driver mutation in genes of a particular
                # signaling pathway and belong to a particular stage of the disease.
                appears = len(set(xp[xp["General Stage"] == st]["Sample ID"]))

                # Some stages are not represented by any samples so at these
                # cases the denominator equals to zero, which is mathematically wrong.
                # Format "perc" to two decimal places.
                try:
                    perc = appears / n_samples_can_path * 100
                    formatted_string = "{:.2f}".format(perc)
                    perc = float(formatted_string)
                except ZeroDivisionError:
                    perc = "not applicable"

                percentages_path.append(perc)

            # As key of the new dictionary item, use the pathway name and the
            # respective n_samples_can_path in parenthesis.
            cancer_d[titles[c] + " (n=" + str(n_samples_can_path) + ")"] = percentages_path
    all_ds_and_titles.append((cancer, cancer_d))



# Plot the data

category_names = ["Stage I", "Stage II", "Stage III", "Stage IV"]

rows = 5
columns = 8
fig, ax = plt.subplots(rows, columns)
fig.set_size_inches(140, 60)

it = -1
for row in range(rows):
    for col in range(columns):
        it += 1
        title = all_ds_and_titles[it][0]
        results = all_ds_and_titles[it][1]
```

```
    labels = list(results.keys())
    data = np.array(list(results.values()))
    data_cum = data.cumsum(axis=1)
    category_colors = plt.get_cmap('RdYlGn')(np.linspace(0.15, 0.85, data.shape[1]))
    ax[row][col].invert_yaxis()
    ax[row][col].set_xlim(0, np.sum(data, axis=1).max())
    # Parameter "pad" refers to the offset of the title from the top of the axes.
    ax[row][col].set_title(title, fontweight='bold', fontsize=14, pad=25)

    for i, (colname, color) in enumerate(zip(category_names, category_colors)):
        widths = data[:, i]
        starts = data_cum[:, i] - widths
        rects = ax[row][col].barh(labels, widths, left=starts, height=0.5,label=colname, color=color)

    ax[row][col].legend(ncol=len(category_names), bbox_to_anchor=(0, 1),loc='lower left',
fontsize='small')
plt.savefig("can_paths_stages.pdf")
plt.show()


alldata = pd.DataFrame(columns=["Cancer Type", "Pathway", "n", "Stage I (%)", "Stage II (%)",
"Stage III (%)", "Stage IV (%)"])
counter = -1
for k in all_ds_and_titles:
    for key in list(k[1].keys()):
        counter += 1
        # "n" is the number of samples of a particular cancer
        # type that harbor at least one driver mutation in genes of
        # a particular signaling pathway.
        n = int(key[:-1].split("=")[1])
        alldata.loc[counter] = [k[0], key.split("(")[0][:-1], n, k[1][key][0], k[1][key][1], k[1][key][2],
k[1][key][3]]
alldata.to_csv("cancers_paths_stages.csv")
```

Program that organizes the information of gene driver alterations at pathway level and visualizes the data (cancer type – cancer stage- altered pathway). IT SETS A n >= 50 CUT-OFF FOR THE CANCER TYPE – SIGNALING PATHWAY PAIRS INCLUDED.

```
cd ~\python programming\2

import pandas as pd
import numpy as np
import matplotlib.pyplot as plt

data = pd.read_csv("driver_mutations_samples_with_stage_available_fixed.csv", index_col=0)
```

```
pg = pd.read_csv("all_pathways_drivers_more_complete.csv", index_col=0)

cantypes =
pd.read_csv("CORRECTED_mutated_genes_per_cancer_type_and_stage_all_cancers.csv")

data = data.drop(columns=["Study", "Stage"]).drop(columns=data.columns[6:])
# Keep only the rows where "General Stage" belongs to the list ["I", "II", "III", "IV"]
no_stage_indices = []
for ind in data.index:
    if type(data.iloc[ind]["General Stage"]) == float:
        no_stage_indices.append(ind)
data = data.drop(no_stage_indices).reset_index().drop(columns="index")

data = data[["Cancer Type", "General Stage", "Sample ID", "Gene"]]
data["Pathway"] = None


# Match genes with the pathways they participate in.
for gene in list(pg["Gene"]):
    paths = ", ".join(list(pg[pg.Gene == gene]["Pathway"]))
    for i in list(data[data.Gene == gene].index):
        data.loc[i, "Pathway"] = paths

# With the following block of code we create a list of all our cancer types
# with a specific order : from the type with the most samples to this with the
# less. This happens due to the appropriate order in which registries are placed
# in cantypes.
cancer_types = []
for can in list(cantypes["Cancer Type"]):
    if can not in cancer_types:
        cancer_types.append(can)

# In patients with stage availiable, no driver mutation was found in TLS and
# Vitamin_cofactor pathways, so we exclude them from the next two variables:
pathways = ["RTK_RAS", "cell_cycle", "p53", "PI3K_Akt", "b_catenin_Wnt", "Notch", "Hippo",
"Myc", "TGFb", "Nrf2", "BER", "NER", "MMR", "FA", "HDR", "NHEJ", "Others_DDR",
"Spliceosome", "Ubq", "Dubq", "Amino_acid", "Carbohydrates", "Energy", "Lipid", "Nucleotide",
"TCA_cycle", "Immunomodulators"]
titles = ["RTK/RAS", "Cell cycle", "p53", "PI3K/Akt", "b catenin/Wnt", "Notch", "Hippo", "Myc",
"TGFb", "Nrf2", "BER", "NER", "MMR", "FA", "HDR", "NHEJ", "Other DDR", "Splicing",
"Ubiquitination", "Deubiquitination", "Amino acids", "Carbohydrates", "Energy", "Lipids",
"Nucleotides", "TCA cycle", "Immunomodulators"]

all_ds_and_titles = []
for cancer in cancer_types:
    x = data[data["Cancer Type"] == cancer].reset_index().drop(columns="index")
    cancer_d = dict()
    c = -1
    for path in pathways:
        c += 1
        indices = []
```

```
        for i in x.index:
            if path in x.iloc[i]["Pathway"]:
                indices.append(i)
        xp = x.iloc[indices]
        # "n_samples_can_path" is the number of samples of a particular cancer
        # type that harbor at least one driver mutation in genes of
        # a particular signaling pathway.
        n_samples_can_path = len(set(xp["Sample ID"]))
        if n_samples_can_path < 50:
            continue
        else:
            percentages_path = []
            for st in ["I", "II", "III", "IV"]:
                # "appears" is the number of samples of a particular cancer type
                # that harbor at least one driver mutation in genes of a particular
                # signaling pathway and belong to a particular stage of the disease.
                appears = len(set(xp[xp["General Stage"] == st]["Sample ID"]))

                # Format "perc" to two decimal places.
                perc = appears / n_samples_can_path * 100
                formatted_string = "{:.2f}".format(perc)
                perc = float(formatted_string)

                percentages_path.append(perc)

            # As key of the new dictionary item, use the pathway name and the
            # respective n_samples_can_path in parenthesis.
            cancer_d[titles[c] + " (n=" + str(n_samples_can_path) + ")"] = percentages_path
    # Retain only cancer types for which there is at least one pathway altered
    # via driver mutation in at least 50 samples.
    if len(cancer_d.keys()) > 0:
        all_ds_and_titles.append((cancer, cancer_d))



# Plot the data

category_names = ["Stage I", "Stage II", "Stage III", "Stage IV"]

rows = 3
columns = 6
fig, ax = plt.subplots(rows, columns)
fig.set_size_inches(140, 60)

it = -1
for row in range(rows):
    for col in range(columns):
        it += 1
        title = all_ds_and_titles[it][0]
        results = all_ds_and_titles[it][1]

        labels = list(results.keys())
```

```
    data = np.array(list(results.values()))
    data_cum = data.cumsum(axis=1)
    category_colors = plt.get_cmap('RdYlGn')(np.linspace(0.15, 0.85, data.shape[1]))
    ax[row][col].invert_yaxis()
    ax[row][col].set_xlim(0, np.sum(data, axis=1).max())
    # Parameter "pad" refers to the offset of the title from the top of the axes.
    ax[row][col].set_title(title, fontweight='bold', fontsize=30, pad=35)

    for i, (colname, color) in enumerate(zip(category_names, category_colors)):
        widths = data[:, i]
        starts = data_cum[:, i] - widths
        rects = ax[row][col].barh(labels, widths, left=starts, height=0.5,label=colname, color=color)

    ax[row][col].legend(ncol=len(category_names), bbox_to_anchor=(0, 1),loc='lower left',
fontsize='medium')
plt.savefig("can_paths_stages_n_at_least_50.pdf")
plt.show()
```

IN ORDER TO SET THE CUT-OFF EQUAL TO 20 :

INSTEAD OF:
if n_samples_can_path < 50:
YOU TYPE:
if n_samples_can_path < 20:

THEN, YOU WILL HAVE INFORMATION FOR 25 PAIRS, SO:

INSTEAD OF:
rows = 3
columns = 6
YOU TYPE:
rows = 5
columns = 5

IN ORDER TO APPLY THE ANALYSIS ONLY AT THE 10 PATHWAYS WE HAVE CHOSEN, THEN WE JUST HAVE TO REPLACE :

pathways = ["RTK_RAS", "cell_cycle", "p53", "PI3K_Akt", "b_catenin_Wnt", "Notch", "Hippo", "Myc", "TGFb", "Nrf2", "BER", "NER", "MMR", "FA", "HDR", "NHEJ", "Others_DDR", "Spliceosome", "Ubq", "Dubq", "Amino_acid", "Carbohydrates", "Energy", "Lipid", "Nucleotide", "TCA_cycle", "Immunomodulators"]
titles = ["RTK/RAS", "Cell cycle", "p53", "PI3K/Akt", "b catenin/Wnt", "Notch", "Hippo", "Myc", "TGFb", "Nrf2", "BER", "NER", "MMR", "FA", "HDR", "NHEJ", "Other DDR", "Splicing", "Ubiquitination", "Deubiquitination", "Amino acids", "Carbohydrates", "Energy", "Lipids", "Nucleotides", "TCA cycle", "Immunomodulators"]

WITH :

pathways = ["RTK_RAS", "cell_cycle", "p53", "PI3K_Akt", "b_catenin_Wnt", "Notch", "HDR", "Spliceosome", "Ubq", "Lipid"]
titles = ["RTK/RAS", "Cell cycle", "p53", "PI3K/Akt", "b catenin/Wnt", "Notch", "HDR", "Splicing", "Ubiquitination", "Lipids"]

ALSO, IN ORDER TO CALCULATE THE PERCENTAGE OF SAMPLES OF EACH CANCER TYPE THAT HARBOR DRIVER MUTATION/S IN EACH PATHWAY AND ALSO ADD THIS INFORMATION INTO THE GRAPH, WE REPLACE :

```
all_ds_and_titles = []
for cancer in cancer_types:
    x = data[data["Cancer Type"] == cancer].reset_index().drop(columns="index")
    cancer_d = dict()
    c = -1
    for path in pathways:
        c += 1
        indices = []
        for i in x.index:
            if path in x.iloc[i]["Pathway"]:
                indices.append(i)
        xp = x.iloc[indices]
        # "n_samples_can_path" is the number of samples of a particular cancer
        # type that harbor at least one driver mutation in genes of
        # a particular signaling pathway.
        n_samples_can_path = len(set(xp["Sample ID"]))
        if n_samples_can_path < 50:
            continue
        else:
            percentages_path = []
            for st in ["I", "II", "III", "IV"]:
                # "appears" is the number of samples of a particular cancer type
                # that harbor at least one driver mutation in genes of a particular
                # signaling pathway and belong to a particular stage of the disease.
                appears = len(set(xp[xp["General Stage"] == st]["Sample ID"]))

                # Format "perc" to two decimal places.
                perc = appears / n_samples_can_path * 100
                formatted_string = "{:.2f}".format(perc)
                perc = float(formatted_string)

                percentages_path.append(perc)

            # As key of the new dictionary item, use the pathway name and the
            # respective n_samples_can_path in parenthesis.
            cancer_d[titles[c] + " (n=" + str(n_samples_can_path) + ")"] = percentages_path
```

```
    # Retain only cancer types for which there is at least one pathway altered
    # via driver mutation in at least 50 samples.
    if len(cancer_d.keys()) > 0:
        all_ds_and_titles.append((cancer, cancer_d))
```

WITH :

```
all_ds_and_titles = []
for cancer in cancer_types:
    x = data[data["Cancer Type"] == cancer].reset_index().drop(columns="index")
    # n_samples_can is the number of samples of a particular cancer type for
    # which there are driver mutational data for the genes of our interest.
    n_samples_can = len(set(x["Sample ID"]))
    cancer_d = dict()
    c = -1
    for path in pathways:
        c += 1
        indices = []
        for i in x.index:
            if path in x.iloc[i]["Pathway"]:
                indices.append(i)
        xp = x.iloc[indices]
        # "n_samples_can_path" is the number of samples of a particular cancer
        # type that harbor at least one driver mutation in genes of
        # a particular signaling pathway.
        n_samples_can_path = len(set(xp["Sample ID"]))

        # psdmp is the percent of samples of a particular cancer type that
        # harbor at least one driver mutation in genes of a particular
        # signaling pathway.
        psdmp = n_samples_can_path / n_samples_can * 100
        form_str = "{:.2f}".format(psdmp)
        psdmp = float(form_str)
        if n_samples_can_path < 20:
            continue
        else:
            percentages_path = []
            for st in ["I", "II", "III", "IV"]:
                # "appears" is the number of samples of a particular cancer type
                # that harbor at least one driver mutation in genes of a particular
                # signaling pathway and belong to a particular stage of the disease.
                appears = len(set(xp[xp["General Stage"] == st]["Sample ID"]))

                # Format "perc" to two decimal places.
                perc = appears / n_samples_can_path * 100
                formatted_string = "{:.2f}".format(perc)
                perc = float(formatted_string)

                percentages_path.append(perc)
```

```
            # As key of the new dictionary item, use the pathway name and the
            # respective n_samples_can_path in parenthesis.
            cancer_d[titles[c] + " (n=" + str(n_samples_can_path) + ", " + str(psdmp) + " %)"] =
percentages_path
    # Retain only cancer types for which there is at least one pathway altered
    # via driver mutation in at least 20 samples.
    if len(cancer_d.keys()) > 0:
        all_ds_and_titles.append((cancer, cancer_d))
```

Program that organizes the information of gene driver alterations at pathway level and visualizes the data (cancer type – cancer stage – altered pathway). IT SETS A n >= 20 CUT-OFF FOR THE CANCER TYPE – SIGNALING PATHWAY PAIRS INCLUDED AND IT COMPRISES ONE MORE INFORMATION : THE PERCENTAGE OF SAMPLES OF EACH CANCER TYPE (AMONG THE SAMPLES FOR WHICH THE DISEASE STAGE IS AVAILABLE), THAT HARBOR DRIVER MUTATIONS IN EACH PATHWAY. Also, the percentage of every sub-bar is shown.

```
cd C:\Users\User\Desktop\ARXEIO-1-9-2021\Paper E.I.E\ΔΙΚΕΣ ΜΟΥ ΑΝΑΖΗΤΗΣΕΙΣ\python programming\2\driver mutations of our 340 cancer genes

import pandas as pd
import numpy as np
import matplotlib.pyplot as plt

all_driver = pd.read_csv("driver_mutations_for_204_cancer_genes.csv", index_col=0)
all_driver.insert(loc=1, column="Pathways", value=None)

cd C:\Users\User\Desktop\ARXEIO-1-9-2021\Paper E.I.E\ΔΙΚΕΣ ΜΟΥ ΑΝΑΖΗΤΗΣΕΙΣ\python programming\2

pg = pd.read_csv("all_pathways_drivers_more_complete.csv", index_col=0)

data = pd.read_csv("driver_mutations_samples_with_stage_available_fixed.csv", index_col=0)

cantypes =
pd.read_csv("CORRECTED_mutated_genes_per_cancer_type_and_stage_all_cancers.csv")

data = data.drop(columns=["Study", "Stage"]).drop(columns=data.columns[6:])
# Keep only the rows where "General Stage" is available (for an unknown
# reason, there are still some rows without stage, even after the first
# cleaning attempt, so we have to clean the data one more time).
no_stage_indices = []
for ind in data.index:
    if type(data.iloc[ind]["General Stage"]) == float:
        no_stage_indices.append(ind)
data = data.drop(no_stage_indices).reset_index().drop(columns="index")

data = data[["Cancer Type", "General Stage", "Sample ID", "Gene"]]
data["Pathway"] = None
```

```
# Match genes with the pathways they participate in.
for gene in list(pg["Gene"]):
    paths = ", ".join(list(pg[pg.Gene == gene]["Pathway"]))
    for file, colpath in [(data, "Pathway"), (all_driver, "Pathways")]:
        for i in list(file[file.Gene == gene].index):
            file.loc[i, colpath] = paths

# With the following block of code we create a list of all our cancer types
# with a specific order : from the type with the most samples to this with the
# less. This happens due to the appropriate order in which registries are placed
# in cantypes.
cancer_types = []
for can in list(cantypes["Cancer Type"]):
    if can not in cancer_types:
        cancer_types.append(can)

# In patients with stage availiable, no driver mutation was found in TLS and
# Vitamin_cofactor pathways, so we exclude them from the next two variables:
pathways = ["RTK_RAS", "cell_cycle", "p53", "PI3K_Akt", "b_catenin_Wnt", "Notch", "Hippo",
"Myc", "TGFb", "Nrf2", "BER", "NER", "MMR", "FA", "HDR", "NHEJ", "Others_DDR",
"Spliceosome", "Ubq", "Dubq", "Amino_acid", "Carbohydrates", "Energy", "Lipid", "Nucleotide",
"TCA_cycle", "Immunomodulators"]
titles = ["RTK/RAS", "Cell cycle", "p53", "PI3K/Akt", "b catenin/Wnt", "Notch", "Hippo", "Myc",
"TGFb", "Nrf2", "BER", "NER", "MMR", "FA", "HDR", "NHEJ", "Other DDR", "Splicing",
"Ubiquitination", "Deubiquitination", "Amino acids", "Carbohydrates", "Energy", "Lipids",
"Nucleotides", "TCA cycle", "Immunomodulators"]

all_ds_and_titles = []
for cancer in cancer_types:
    x = data[data["Cancer Type"] == cancer].reset_index().drop(columns="index")
    # n_samples_can is the total number of samples of a particular cancer type
    # that harbor at least one driver mutation in at least one of our 204 genes
    # of interest and their stage is available (samples from TCGA PanCancer Atlas
    # studies).
    n_samples_can = len(set(x["Sample ID"]))
    cancer_d = dict()
    c = -1
    for path in pathways:
        c += 1
        indices = []
        for i in x.index:
            if path in x.iloc[i]["Pathway"]:
                indices.append(i)
        xp = x.iloc[indices]
        # "n_samples_can_path_st" is the number of samples of a particular cancer
        # type that harbor at least one driver mutation in genes of
        # a particular signaling pathway and their disease stage is available.
        n_samples_can_path_st = len(set(xp["Sample ID"]))

        if n_samples_can_path_st < 20:
            continue
```

```
        else:
            # pdm is the percentage of samples of a particular cancer
            # type that harbor at least one driver mutation in genes of
            # a particular signaling pathway and their disease stage is available.
            pdm = n_samples_can_path_st / n_samples_can * 100
            form_str = "{:.2f}".format(pdm)
            pdm = float(form_str)
            percentages_path = []
            for st in ["I", "II", "III", "IV"]:
                # "appears" is the number of samples of a particular cancer type
                # that harbor at least one driver mutation in genes of a particular
                # signaling pathway and belong to a particular stage of the disease.
                appears = len(set(xp[xp["General Stage"] == st]["Sample ID"]))

                # Format "perc" to two decimal places.
                perc = appears / n_samples_can_path_st * 100
                formatted_string = "{:.2f}".format(perc)
                perc = float(formatted_string)

                percentages_path.append(perc)

            # As key of the new dictionary item, use the pathway name and the
            # respective n_samples_can_path_st in parenthesis.
            cancer_d[titles[c] + " (n=" + str(n_samples_can_path_st) + ", pdm=" + str(pdm) + " %)"] =
percentages_path
    # Retain only cancer types for which there is at least one pathway altered
    # via driver mutation in at least 20 samples.
    if len(cancer_d.keys()) > 0:
            titl = cancer + " (N=" + str(n_samples_can) + ")"
            if len(cancer_d.keys()) == 1:
                all_ds_and_titles.append((titl, cancer_d))
            elif len(cancer_d.keys()) > 1:
                # sort cancer_d items by pdm in order to show the bars of the graph
                # in a descending pdm order.
                pn = []
                for k in cancer_d.keys():
                    pn.append((k.split("pdm=")[0], float(k.split("pdm=")[1][:-3])))
                cancer_d_sorted = {}
                # key = lambda x: x[1] ensures the classification will take place
                # using the second item of each tuple of the list.
                for s in sorted(pn, key = lambda x: x[1], reverse=True):
                    for ke in cancer_d.keys():
                        if s[0] in ke:
                            cancer_d_sorted[ke] = cancer_d[ke]
                            break
                all_ds_and_titles.append((titl, cancer_d_sorted))



# Plot the data

category_names = ["Stage I", "Stage II", "Stage III", "Stage IV"]
```

```
rows = 5
columns = 5
fig, ax = plt.subplots(rows, columns)
fig.set_size_inches(140, 60)

it = -1
for row in range(rows):
    for col in range(columns):
        it += 1
        title = all_ds_and_titles[it][0]
        results = all_ds_and_titles[it][1]

        labels = list(results.keys())
        data = np.array(list(results.values()))
        data_cum = data.cumsum(axis=1)
        category_colors = plt.get_cmap('RdYlGn')(np.linspace(0.15, 0.85, data.shape[1]))
        ax[row][col].invert_yaxis()
        ax[row][col].set_xlim(0, np.sum(data, axis=1).max())
        # Parameter "pad" refers to the offset of the title from the top of the axes.
        ax[row][col].set_title(title, fontweight='bold', fontsize=30, pad=35)

        for i, (colname, color) in enumerate(zip(category_names, category_colors)):
            widths = data[:, i]
            starts = data_cum[:, i] - widths
            rects = ax[row][col].barh(labels, widths, left=starts, height=0.5,label=colname, color=color)

            r, g, b, _ = color
            text_color = 'white' if r * g * b < 0.5 else 'darkgrey'
            ax[row][col].bar_label(rects, label_type='center', color=text_color, fontsize=10)

        ax[row][col].legend(ncol=len(category_names), bbox_to_anchor=(0, 1),loc='lower left',
fontsize=10)
plt.savefig("CORRECTED_can_paths_stages_n_at_least_20_with_percent_sorted.pdf")
plt.show()
```

Import clinical data that refer to patients profiled for their mutations and keep only entries referring to samples originating from primary tumors (not recurrences or metastases). Also, for each entry, add the general stage at which the corresponding sample belongs to.

```
cd ~\python programming\2

import pandas as pd

all_clin = pd.read_csv("clinical_data_of_samples_profilied_for_their_mutations.tsv", sep="\t")

for t in list(set(all_clin["Sample Type"])):
    print(t, len(all_clin[all_clin["Sample Type"] == t]), "\n")
    # The result is : Recurrence --> 9 samples, Primary --> 10066 samples and
```

```
    # Metastasis --> 364 samples

# Keep only data referring to primary tumors.
all_clin = all_clin[all_clin["Sample Type"] == "Primary"].reset_index().drop(columns="index")

# Ensure that there are no multiple entries for the patients.
len(all_clin) == len(set(all_clin["Patient ID"]))

stage_IS = ["STAGE IS"]
stage_I = ["STAGE I", "STAGE IA", "STAGE IB"]
stage_II = ["STAGE II", "STAGE IIA", "STAGE IIB", "STAGE IIC"]
stage_III = ["STAGE III", "STAGE IIIA", "STAGE IIIB", "STAGE IIIC"]
stage_IV = ["STAGE IV", "STAGE IVA", "STAGE IVB", "STAGE IVC"]
stage_X = ["STAGE X"]

stages = [("IS", stage_IS), ("I", stage_I), ("II", stage_II), ("III", stage_III), ("IV", stage_IV), ("X",
stage_X)]

all_clin.insert(loc=5, column="General Stage", value="")

for i in range(len(all_clin)):
    x = all_clin.iloc[i]["Neoplasm Disease Stage American Joint Committee on Cancer Code"]
    for z in range(len(stages)):
        if x in stages[z][1]:
            all_clin.loc[i, "General Stage"] = stages[z][0]

cd ~\python programming\3
all_clin.to_csv("clinical_data_of_samples_profilied_for_their_mutations_and_from_primary_origin
.csv")
```

Create a file containing driver mutations for our 204 genes of interest, but only for patients included to the new dataset as this was set in the previous program.

```
cd ~\python programming\2

drivmuts = pd.read_csv("driver_mutations_for_204_cancer_genes_includes_pathways.csv",
index_col=0)

cd ~\python programming\3

import pandas as pd

all_clin =
pd.read_csv("clinical_data_of_samples_profilied_for_their_mutations_and_from_primary_origin.cs
v", index_col=0)
```

```
# From "driver_mutations_for_204_cancer_genes_includes_pathways.csv", keep only
# entries referring to samples included in
# "clinical_data_of_samples_profilied_for_their_mutations_and_from_primary_origin.csv"
indices = []
for ID in list(set(all_clin["Sample ID"])):
    if ID in list(set(drivmuts["Sample ID"])):
        for ind in drivmuts[drivmuts["Sample ID"] == ID].index:
            indices.append(ind)
drivmuts = drivmuts.iloc[indices].reset_index().drop(columns="index")
drivmuts.to_csv("driver_mutations_for_204_cancer_genes_includes_pathways_new_dataset_prima
ry_tumors.csv")
```

Choose only cancer types for which there are at least 50 samples (=patients, because of the 1:1 ratio) available in our new dataset, find the proportion of driver mutation existence for each signaling pathway and create a heatmap in order to visualize the findings.

```
cd ~\python programming\3

import pandas as pd
import numpy as np
import matplotlib.pyplot as plt

drivmuts =
pd.read_csv("driver_mutations_for_204_cancer_genes_includes_pathways_new_dataset_primary_t
umors.csv", index_col=0)
clinical_data =
pd.read_csv("clinical_data_of_samples_profilied_for_their_mutations_and_from_primary_origin.cs
v", index_col=0)

freq = []
for ct in list(set(drivmuts["Cancer Type"])):
    freq.append((ct, len(set(drivmuts[drivmuts["Cancer Type"] == ct]["Sample ID"]))))
freq = sorted(freq, key = lambda x: x[1], reverse=True)

# Keep only cancer types with at least 50 samples (and therefore patients) available.
cancers = []
cancer_labels = []
for p in freq:
    if p[1] >= 50:
        cancers.append(p[0])
        cancer_labels.append(p[0] + " (n=" + str(p[1]) + ")")

# Isolate the useful piece of drivmuts (the one that contains only cancers with
# at least 50 available samples).
indices = []
for cancer in cancers:
    for ind in drivmuts[drivmuts["Cancer Type"] == cancer].index:
```

```
        indices.append(ind)
drivmuts = drivmuts.iloc[indices].reset_index().drop(columns="index")

# Find all the pahways implicated via driver mutation at the selected cancers.
# For each pathway, find also the number of samples affected in order to sort
# the pathways by it.
pathways = []
for paths in list(set(drivmuts.Pathways)):
    for path in paths.split(", "):
        if path not in pathways:
            samples = []
            for IND in drivmuts.index:
                if path in drivmuts.iloc[IND]["Pathways"]:
                    if drivmuts.iloc[IND]["Sample ID"] not in samples:
                        samples.append(drivmuts.iloc[IND]["Sample ID"])
            pathways.append((path, len(samples)))
# For some reason, in "pathways" there are more than one copies of each entry,
# so we have to fix this.
pathways = list(set(pathways))
pathways = sorted(pathways, key = lambda x: x[1], reverse=True)
paths_collection, junk = zip(*pathways)

cascades = ["RTK_RAS", "cell_cycle", "p53", "PI3K_Akt", "b_catenin_Wnt", "Notch", "Hippo",
"Myc", "TGFb", "Nrf2", "BER", "NER", "MMR", "FA", "HDR", "NHEJ", "Others_DDR",
"Spliceosome", "Ubq", "Dubq", "Amino_acid", "Carbohydrates", "Energy", "Lipid", "Nucleotide",
"TCA_cycle", "Immunomodulators"]
titles = ["RTK/RAS", "Cell cycle", "p53", "PI3K/Akt", "b catenin/Wnt", "Notch", "Hippo", "Myc",
"TGFb", "Nrf2", "BER", "NER", "MMR", "FA", "HDR", "NHEJ", "Other DDR", "Splicing",
"Ubiquitination", "Deubiquitination", "Amino acids", "Carbohydrates", "Energy", "Lipids",
"Nucleotides", "TCA cycle", "Immunomodulators"]

pathway_labels = []
for entry in pathways:
    pathway_labels.append(titles[cascades.index(entry[0])])



# Build our data
data_list = []
for can in cancers:
    x = drivmuts[drivmuts["Cancer Type"] == can].reset_index().drop(columns="index")
    # n_samples_can is the number of samples ( = patients) that are profiled
    # for their mutations, originate from primary tumor site and refer to a
    # particular cancer type.
    n_samples_can = len(set(clinical_data[clinical_data["Cancer Type Detailed"] == can]["Sample
ID"]))
    csl = []
    for cascade in paths_collection:
        inds = []
        for i in x.index:
            if cascade in x.iloc[i]["Pathways"]:
                inds.append(i)
```

```
        # n_samples_can_path is the number of samples ( = patients) that are
        # profiled for their mutations, originate from primary tumor site,
        # refer to a particular cancer type and are altered via driver
        # mutation in a particular pathway.
        n_samples_can_path = len(set(x.iloc[inds]["Sample ID"]))

        # Format "perc" to two decimal places.
        perc = n_samples_can_path / n_samples_can * 100
        formatted_string = "{:.2f}".format(perc)
        perc = float(formatted_string)
        csl.append(perc)
    data_list.append(csl)
data = np.array(data_list)


# Plot the data
def heatmap(data, row_labels, col_labels, ax=None,
            cbar_kw={}, cbarlabel="", **kwargs):
    """
    Create a heatmap from a numpy array and two lists of labels.

    Parameters
    ----------
    data
        A 2D numpy array of shape (N, M).
    row_labels
        A list or array of length N with the labels for the rows.
    col_labels
        A list or array of length M with the labels for the columns.
    ax
        A `matplotlib.axes.Axes` instance to which the heatmap is plotted.  If
        not provided, use current axes or create a new one.  Optional.
    cbar_kw
        A dictionary with arguments to `matplotlib.Figure.colorbar`.  Optional.
    cbarlabel
        The label for the colorbar.  Optional.
    **kwargs
        All other arguments are forwarded to `imshow`.
    """

    if not ax:
        ax = plt.gca()

    # Plot the heatmap
    im = ax.imshow(data, **kwargs)

    # Create colorbar
    cbar = ax.figure.colorbar(im, ax=ax, **cbar_kw)
    cbar.ax.set_ylabel(cbarlabel, rotation=-90, va="bottom")

    # We want to show all ticks...
    ax.set_xticks(np.arange(data.shape[1]))
```

```
    ax.set_yticks(np.arange(data.shape[0]))
    # ... and label them with the respective list entries.
    ax.set_xticklabels(col_labels)
    ax.set_yticklabels(row_labels)

    # Let the horizontal axes labeling appear on top.
    ax.tick_params(top=True, bottom=False,
                   labeltop=True, labelbottom=False)

    # Rotate the tick labels and set their alignment.
    plt.setp(ax.get_xticklabels(), rotation=-55, ha="right",
             rotation_mode="anchor")

    # Turn spines off and create white grid.
    ax.spines[:].set_visible(False)

    ax.set_xticks(np.arange(data.shape[1]+1)-.5, minor=True)
    ax.set_yticks(np.arange(data.shape[0]+1)-.5, minor=True)
    ax.grid(which="minor", color="w", linestyle='-', linewidth=3)
    ax.tick_params(which="minor", bottom=False, left=False)

    return im, cbar


def annotate_heatmap(im, data=None, valfmt="{x:.2f}",
                     textcolors=("black", "white"),
                     threshold=None, **textkw):
    """
    A function to annotate a heatmap.

    Parameters
    ----------
    im
        The AxesImage to be labeled.
    data
        Data used to annotate.  If None, the image's data is used.  Optional.
    valfmt
        The format of the annotations inside the heatmap.  This should either
        use the string format method, e.g. "$ {x:.2f}", or be a
        `matplotlib.ticker.Formatter`.  Optional.
    textcolors
        A pair of colors.  The first is used for values below a threshold,
        the second for those above.  Optional.
    threshold
        Value in data units according to which the colors from textcolors are
        applied.  If None (the default) uses the middle of the colormap as
        separation.  Optional.
    **kwargs
        All other arguments are forwarded to each call to `text` used to create
        the text labels.
    """
```

```
    if not isinstance(data, (list, np.ndarray)):
        data = im.get_array()

    # Normalize the threshold to the images color range.
    if threshold is not None:
        threshold = im.norm(threshold)
    else:
        threshold = im.norm(data.max())/2.

    # Set default alignment to center, but allow it to be
    # overwritten by textkw.
    kw = dict(horizontalalignment="center",
              verticalalignment="center")
    kw.update(textkw)

    # Get the formatter in case a string is supplied
    if isinstance(valfmt, str):
        valfmt = matplotlib.ticker.StrMethodFormatter(valfmt)

    # Loop over the data and create a `Text` for each "pixel".
    # Change the text's color depending on the data.
    texts = []
    for i in range(data.shape[0]):
        for j in range(data.shape[1]):
            kw.update(color=textcolors[int(im.norm(data[i, j]) > threshold)])
            text = im.axes.text(j, i, valfmt(data[i, j], None), **kw)
            texts.append(text)

    return texts

fig, ax = plt.subplots()
fig.set_size_inches(10, 8)

im, cbar = heatmap(data, cancer_labels, pathway_labels, ax=ax, cmap="YlGn", cbarlabel="Driver mutational rate (%)")
texts = annotate_heatmap(im, valfmt="{x:.2f}")

fig.tight_layout()
plt.show()
```

Make the same things as at the previous 3 programs, but this time for metastatic samples. Here, there is only one cancer type to deal with (Cutaneous Melanoma), so we make our visualization in the context of comparison of primary versus metastatic samples in terms of signaling pathways' driver mutations.

```
cd ~\python programming\2
```

```
import pandas as pd
import numpy as np
import matplotlib.pyplot as plt

all_clin = pd.read_csv("clinical_data_of_samples_profilied_for_their_mutations.tsv", sep="\t")
drivmuts = pd.read_csv("driver_mutations_for_204_cancer_genes_includes_pathways.csv",
index_col=0)

for t in list(set(all_clin["Sample Type"])):
    print(t, len(all_clin[all_clin["Sample Type"] == t]), "\n")
    # The result is : Recurrence --> 9 samples, Primary --> 10066 samples and
    # Metastasis --> 364 samples

# Keep only data referring to metastatic tumors.
all_clin = all_clin[all_clin["Sample Type"] == "Metastasis"].reset_index().drop(columns="index")

# Ensure that there are no multiple entries for the patients.
len(all_clin) == len(set(all_clin["Patient ID"]))

stage_IS = ["STAGE IS"]
stage_I = ["STAGE I", "STAGE IA", "STAGE IB"]
stage_II = ["STAGE II", "STAGE IIA", "STAGE IIB", "STAGE IIC"]
stage_III = ["STAGE III", "STAGE IIIA", "STAGE IIIB", "STAGE IIIC"]
stage_IV = ["STAGE IV", "STAGE IVA", "STAGE IVB", "STAGE IVC"]
stage_X = ["STAGE X"]

stages = [("IS", stage_IS), ("I", stage_I), ("II", stage_II), ("III", stage_III), ("IV", stage_IV), ("X",
stage_X)]

all_clin.insert(loc=5, column="General Stage", value="")

for i in range(len(all_clin)):
    x = all_clin.iloc[i]["Neoplasm Disease Stage American Joint Committee on Cancer Code"]
    for z in range(len(stages)):
        if x in stages[z][1]:
            all_clin.loc[i, "General Stage"] = stages[z][0]

cd ~\python programming\3
all_clin.to_csv("clinical_data_of_samples_profilied_for_their_mutations_and_from_metastatic_ori
gin.csv")




# From "driver_mutations_for_204_cancer_genes_includes_pathways.csv", keep only
# entries referring to samples included in
# "clinical_data_of_samples_profilied_for_their_mutations_and_from_metastatic_origin.csv"
indices = []
for ID in list(set(all_clin["Sample ID"])):
    if ID in list(set(drivmuts["Sample ID"])):
        for ind in drivmuts[drivmuts["Sample ID"] == ID].index:
```

```
        indices.append(ind)
drivmuts = drivmuts.iloc[indices].reset_index().drop(columns="index")
drivmuts.to_csv("driver_mutations_for_204_cancer_genes_includes_pathways_new_dataset_metas
tatic_tumors.csv")




freq = []
for ct in list(set(drivmuts["Cancer Type"])):
    freq.append((ct, len(set(drivmuts[drivmuts["Cancer Type"] == ct]["Sample ID"]))))
freq = sorted(freq, key = lambda x: x[1], reverse=True)
# From the above, it arises that there are data for 2 metastatic cancer types:
# Cutaneous Melanoma (352 samples) and Papillary Thyroid Cancer (1 sample). So,
# from now on, we will focus on Cutaneous Melanoma.
freq.pop()

drivmuts_prim =
pd.read_csv("driver_mutations_for_204_cancer_genes_includes_pathways_new_dataset_primary_t
umors.csv", index_col=0)
freq.append((freq[0][0], len(set(drivmuts_prim[drivmuts_prim["Cancer Type"] ==
freq[0][0]]["Sample ID"]))))

can = freq[0][0]
cancer_labels = []
c = 0
for p in freq:
    if c == 0:
        cancer_labels.append("Metastatic " + p[0] + " (n=" + str(p[1]) + ")")
    else:
        cancer_labels.append("Primary " + p[0] + " (n=" + str(p[1]) + ")")
    c += 1

# Isolate the piece of drivmuts and drivmuts_prim that contains Cutaneous Melanoma.

drivmuts = drivmuts.iloc[drivmuts[drivmuts["Cancer Type"] ==
can].index].reset_index().drop(columns="index")
drivmuts_prim = drivmuts_prim.iloc[drivmuts_prim[drivmuts_prim["Cancer Type"] ==
can].index].reset_index().drop(columns="index")

# Find all the pahways implicated via driver mutation at the selected cancers
# (metastatic and primary Cutaneous Melanoma). For each pathway, find also the
# number of samples affected in order to sort the pathways by it.
mix = pd.concat([drivmuts, drivmuts_prim]).reset_index().drop(columns="index")
pathways = []
for paths in list(set(mix.Pathways)):
    for path in paths.split(", "):
        if path not in pathways:
            samples = []
            for IND in mix.index:
                if path in mix.iloc[IND]["Pathways"]:
```

```
            if mix.iloc[IND]["Sample ID"] not in samples:
                samples.append(mix.iloc[IND]["Sample ID"])
        pathways.append((path, len(samples)))
# For some reason, in "pathways" there are more than one copies of each entry,
# so we have to fix this.
pathways = list(set(pathways))
pathways = sorted(pathways, key = lambda x: x[1], reverse=True)
paths_collection, junk = zip(*pathways)

cascades = ["RTK_RAS", "cell_cycle", "p53", "PI3K_Akt", "b_catenin_Wnt", "Notch", "Hippo",
"Myc", "TGFb", "Nrf2", "BER", "NER", "MMR", "FA", "HDR", "NHEJ", "Others_DDR",
"Spliceosome", "Ubq", "Dubq", "Amino_acid", "Carbohydrates", "Energy", "Lipid", "Nucleotide",
"TCA_cycle", "Immunomodulators"]
titles = ["RTK/RAS", "Cell cycle", "p53", "PI3K/Akt", "b catenin/Wnt", "Notch", "Hippo", "Myc",
"TGFb", "Nrf2", "BER", "NER", "MMR", "FA", "HDR", "NHEJ", "Other DDR", "Splicing",
"Ubiquitination", "Deubiquitination", "Amino acids", "Carbohydrates", "Energy", "Lipids",
"Nucleotides", "TCA cycle", "Immunomodulators"]

pathway_labels = []
for entry in pathways:
    pathway_labels.append(titles[cascades.index(entry[0])])

all_clin_prim =
pd.read_csv("clinical_data_of_samples_profilied_for_their_mutations_and_from_primary_origin.cs
v")

# Build our data
data_list = []
for x, y in [(drivmuts, all_clin), (drivmuts_prim, all_clin_prim)]:
    # n_samples_can is the number of samples ( = patients) that are profiled
    # for their mutations, originate from primary tumor site and refer to a
    # particular cancer type.
    n_samples_can = len(set(y[y["Cancer Type Detailed"] == can]["Sample ID"]))
    csl = []
    for cascade in paths_collection:
        inds = []
        for i in x.index:
            if cascade in x.iloc[i]["Pathways"]:
                inds.append(i)
        # n_samples_can_path is the number of samples ( = patients) that are
        # profiled for their mutations, originate from primary tumor site,
        # refer to a particular cancer type and are altered via driver
        # mutation in a particular pathway.
        n_samples_can_path = len(set(x.iloc[inds]["Sample ID"]))

        # Format "perc" to two decimal places.
        perc = n_samples_can_path / n_samples_can * 100
        formatted_string = "{:.2f}".format(perc)
        perc = float(formatted_string)
        csl.append(perc)
    data_list.append(csl)
data = np.array(data_list)
```

```
# Plot the data
def heatmap(data, row_labels, col_labels, ax=None,
            cbar_kw={}, cbarlabel="", **kwargs):
    """
    Create a heatmap from a numpy array and two lists of labels.

    Parameters
    ----------
    data
        A 2D numpy array of shape (N, M).
    row_labels
        A list or array of length N with the labels for the rows.
    col_labels
        A list or array of length M with the labels for the columns.
    ax
        A `matplotlib.axes.Axes` instance to which the heatmap is plotted.  If
        not provided, use current axes or create a new one.  Optional.
    cbar_kw
        A dictionary with arguments to `matplotlib.Figure.colorbar`.  Optional.
    cbarlabel
        The label for the colorbar.  Optional.
    **kwargs
        All other arguments are forwarded to `imshow`.
    """

    if not ax:
        ax = plt.gca()

    # Plot the heatmap
    im = ax.imshow(data, **kwargs)

    # Create colorbar
    cbar = ax.figure.colorbar(im, ax=ax, **cbar_kw)
    cbar.ax.set_ylabel(cbarlabel, rotation=-90, va="bottom")

    # We want to show all ticks...
    ax.set_xticks(np.arange(data.shape[1]))
    ax.set_yticks(np.arange(data.shape[0]))
    # ... and label them with the respective list entries.
    ax.set_xticklabels(col_labels)
    ax.set_yticklabels(row_labels)

    # Let the horizontal axes labeling appear on top.
    ax.tick_params(top=True, bottom=False,
                   labeltop=True, labelbottom=False)

    # Rotate the tick labels and set their alignment.
    plt.setp(ax.get_xticklabels(), rotation=-55, ha="right",
             rotation_mode="anchor")
```

```
    # Turn spines off and create white grid.
    ax.spines[:].set_visible(False)

    ax.set_xticks(np.arange(data.shape[1]+1)-.5, minor=True)
    ax.set_yticks(np.arange(data.shape[0]+1)-.5, minor=True)
    ax.grid(which="minor", color="w", linestyle='-', linewidth=3)
    ax.tick_params(which="minor", bottom=False, left=False)

    return im, cbar


def annotate_heatmap(im, data=None, valfmt="{x:.2f}",
                     textcolors=("black", "white"),
                     threshold=None, **textkw):
    """
    A function to annotate a heatmap.

    Parameters
    ----------
    im
        The AxesImage to be labeled.
    data
        Data used to annotate.  If None, the image's data is used.  Optional.
    valfmt
        The format of the annotations inside the heatmap.  This should either
        use the string format method, e.g. "$ {x:.2f}", or be a
        `matplotlib.ticker.Formatter`.  Optional.
    textcolors
        A pair of colors.  The first is used for values below a threshold,
        the second for those above.  Optional.
    threshold
        Value in data units according to which the colors from textcolors are
        applied.  If None (the default) uses the middle of the colormap as
        separation.  Optional.
    **kwargs
        All other arguments are forwarded to each call to `text` used to create
        the text labels.
    """

    if not isinstance(data, (list, np.ndarray)):
        data = im.get_array()

    # Normalize the threshold to the images color range.
    if threshold is not None:
        threshold = im.norm(threshold)
    else:
        threshold = im.norm(data.max())/2.

    # Set default alignment to center, but allow it to be
    # overwritten by textkw.
    kw = dict(horizontalalignment="center",
              verticalalignment="center")
```

```
    kw.update(textkw)

    # Get the formatter in case a string is supplied
    if isinstance(valfmt, str):
        valfmt = matplotlib.ticker.StrMethodFormatter(valfmt)

    # Loop over the data and create a `Text` for each "pixel".
    # Change the text's color depending on the data.
    texts = []
    for i in range(data.shape[0]):
        for j in range(data.shape[1]):
            kw.update(color=textcolors[int(im.norm(data[i, j]) > threshold)])
            text = im.axes.text(j, i, valfmt(data[i, j], None), **kw)
            texts.append(text)

    return texts

fig, ax = plt.subplots()
# fig.set_size_inches(10, 8)

im, cbar = heatmap(data, cancer_labels, pathway_labels, ax=ax, cmap="YlGn", cbarlabel="Driver mutational rate (%)")
texts = annotate_heatmap(im, valfmt="{x:.2f}")

fig.tight_layout()
plt.show()

# The most interesting differences were shown for "p53" and "Cell cycle" paths,
# with a higher proportion of driver mutations found in metastatic vs primary
# Cutaneous Melanoma samples (27,82 vs 16,88 and 21,49 vs 11,69 respectively).
```

Find all genes found mutated (via driver mutation) only in metastatic but not in the primary cutaneous melanoma patients and sort them by their occurrence percentage.

```
cd ~\python programming\3

import pandas as pd
import numpy as np
import matplotlib.pyplot as plt

mut_prim = pd.read_csv("driver_mutations_for_204_cancer_genes_includes_pathways_new_dataset_primary_tumors.csv", index_col=0)
mut_prim = mut_prim[mut_prim["Cancer Type"] == "Cutaneous Melanoma"]

mut_metast = pd.read_csv("driver_mutations_for_204_cancer_genes_includes_pathways_new_dataset_metastatic_tumors.csv", index_col=0)
mut_metast = mut_metast[mut_metast["Cancer Type"] == "Cutaneous Melanoma"]

only_metast = []
```

```
for gene in list(set(mut_metast["Gene"])):
    if gene not in set(mut_prim["Gene"]):
        # Calculate the proportion of cutaneous melanoma patients that harbor
        # driver mutation in this gene and format to two decimal places.
        perc = len(set(mut_metast[mut_metast["Gene"] == gene]["Sample ID"])) /
len(set(mut_metast["Sample ID"])) * 100
        formatted_string = "{:.2f}".format(perc)
        perc = float(formatted_string)

        paths = mut_metast[mut_metast["Gene"] == gene].iloc[0]["Pathways"]
        only_metast.append((gene, paths, perc))

only_metast =  sorted(only_metast, key = lambda x: x[2], reverse=True)
# Check the different FLT3 mutations occuring in metastatic cutaneous
# melanoma patients
set(mut_metast[mut_metast["Gene"] == "FLT3"]["Protein Change"])

# FLT3 harbors driver mutations in 4,83% of metastatic cutaneous melanoma
# patients but in no one of the primary. 13 different mutations were found, all
# inside the protein kinase domain of FLT3 gene, according to UniProt (P36888).

# Make the opposite work as well.
only_prim = []
for gene in list(set(mut_prim["Gene"])):
    if gene not in set(mut_metast["Gene"]):
        # Calculate the proportion of cutaneous melanoma patients that harbor
        # driver mutation in this gene and format to two decimal places.
        perc = len(set(mut_prim[mut_prim["Gene"] == gene]["Sample ID"])) /
len(set(mut_prim["Sample ID"])) * 100
        formatted_string = "{:.2f}".format(perc)
        perc = float(formatted_string)

        paths = mut_prim[mut_prim["Gene"] == gene].iloc[0]["Pathways"]
        only_prim.append((gene, paths, perc))

only_prim =  sorted(only_prim, key = lambda x: x[2], reverse=True)
# We found only two genes being altered via somatic driver mutation exclusively
# in primary and not in metastatic tumors. These are LRP5 and DTX1, which
# however were found only in one primary sample each.

# Check the cancer type of primary tumors (if exist) that harbor each of the
# FLT3 mutations found in metastatic cutaneous melanoma patients.
data =
pd.read_csv("driver_mutations_for_204_cancer_genes_includes_pathways_new_dataset_primary_t
umors.csv", index_col=0)
flt3 = data[data.Gene == "FLT3"]
w = []
for alt in set(mut_metast[mut_metast["Gene"] == "FLT3"]["Protein Change"]):
    w.append((alt, set(flt3[flt3["Protein Change"] == alt]["Cancer Type"])))
```

Isolate only driver mutations of Head and Neck Squamous Cell Carcinoma patients and only clinical data of HNSCC patients that harbor at least one driver mutation on our 204 selected genes of interest.

```
cd ~\python programming\3

import pandas as pd

mut_prim =
pd.read_csv("driver_mutations_for_204_cancer_genes_includes_pathways_new_dataset_primary_t
umors.csv", index_col=0)
clin =
pd.read_csv("clinical_data_of_samples_profilied_for_their_mutations_and_from_primary_origin.cs
v", index_col=0)

data = mut_prim[mut_prim["Cancer Type"] == 'Head and Neck Squamous Cell Carcinoma']
data.sort_values(by="Sample ID")

data.to_csv("driver_mutations_Head_and_Neck_Squamous_Cell_Carcinoma_includes_pathways_p
rimary_tumors.csv")

# In clin, there are entries for 515 patients with a Head and Neck Squamous
# Cell Carcinoma diagnosis. However in mut_prim, there are entries for 473
# patients. This happens because in TCGA PanCaner Atlas projects' dataset,
# there are 515 - 473 = 42 patients with the above diagnosis, that do not
# harbor any driver mutations on our 204 selected genes of interest.

indices = []
for i in clin.index:
    if clin.iloc[i]["Sample ID"] not in set(data["Sample ID"]):
        indices.append(i)
clin = clin.drop(indices)
clin.to_csv("clinical_data_Head_and_Neck_Squamous_Cell_Carcinoma_primary_tumors.csv")
```

Program that isolates for each signaling pathway the samples harboring driver mutations on this pathway and then it calculates the percentage of them that harbor driver mutation in each gene of this pathway. Finally, it sorts the data by the calculated percentages.

```
cd ~\python programming\3
import pandas as pd
data =
pd.read_csv("driver_mutations_for_204_cancer_genes_includes_pathways_new_dataset_primary_t
umors.csv", index_col=0)
cascades = ["RTK_RAS", "cell_cycle", "p53", "PI3K_Akt", "b_catenin_Wnt", "Notch", "Hippo",
```

```
"Myc", "TGFb", "Nrf2", "BER", "NER", "MMR", "FA", "HDR", "NHEJ", "Others_DDR",
"Spliceosome", "Ubq", "Dubq", "Amino_acid", "Carbohydrates", "Energy", "Lipid", "Nucleotide",
"TCA_cycle", "Immunomodulators"]

# Function to sort the list by second item of tuple
def Sort_Tuple(tup):

        # reverse = True (Sorts in Descending order)
        # key is set to sort using second element of
        # sublist lambda has been used
        return(sorted(tup, key = lambda x: x[1], reverse=True))


final = []
for casc in cascades:
   info = []
   inds = []
   for i in data.index:
      if casc in data.iloc[i]["Pathways"]:
         inds.append(i)
   casc_data = data.iloc[inds]
   for gene in list(set(casc_data.Gene)):
      # "p" is the percentage of samples affected in a particular pathway,
      # that harbor driver mutation in a particular gene of this pathway.
      p = len(set(casc_data[casc_data.Gene == gene]["Sample ID"])) / len(set(casc_data["Sample
ID"])) * 100
      formatted_string = "{:.2f}".format(p)
      p = float(formatted_string)
      info.append((gene, p))
   info = Sort_Tuple(info)
   final.append((casc, info))
```

Sort *TP53* gene alterations by their occurrence in order to find the most frequent ones. Given that almost all mutations of this gene occur in three-digit positions, the following program deals only with those positions.

```
cd ~\python programming\3

import pandas as pd
from collections import Counter

data =
pd.read_csv("driver_mutations_for_204_cancer_genes_includes_pathways_new_dataset_primary_t
umors.csv", index_col=0)
part = data[data.Gene == "TP53"].reset_index().drop(columns="index")

# Sort TP53 mutations by their frequency
mf = Counter(list(part["Protein Change"])).most_common()

xs, ys = zip(*mf)
```

```
# Find TP53 substitutions affecting R175, R273 and R248 positions.
nons = []
for pos in ["R175", "R248", "R273"]:
    case = []
    for mut in xs:
        if mut.startswith(pos) and len(mut) == 5 and "*" not in mut:
            case.append(mut)
    nons.append(case)

# Calculate the proportion of samples with mutated TP53, that harbor a
# substitution in one of the most frequently substituted positions you found above.
rank = []
for k in range(len(nons)):
    inds = []
    for i in part.index:
        if part.iloc[i]["Protein Change"] in nons[k]:
            inds.append(i)
    p = len(set(part.iloc[inds]["Sample ID"])) / len(set(part["Sample ID"])) * 100
    p = float("{:.2f}".format(p))
    rank.append((nons[k][0][:-1], p))
rank = sorted(rank, key=lambda x: x[1], reverse=True)
```

Something similar for *CDKN2A* gene.

```
cd ~\python programming\3

import pandas as pd
from collections import Counter

data =
pd.read_csv("driver_mutations_for_204_cancer_genes_includes_pathways_new_dataset_primary_t
umors.csv", index_col=0)
part = data[data.Gene == "CDKN2A"].reset_index().drop(columns="index")

# Sort CDKN2A mutations by their frequency
mf = Counter(list(part["Protein Change"])).most_common()

# Find the most frequent (at least 5 registries) nonsense mutations of CDKN2A gene.
nons = []
for mut in mf:
    if mut[1] < 5:
        continue
    else:
        # I think that mutations with an "*" in their name refer to nonsense
        # mutations. Let's become sure of that.
        if "*" in mut[0]:
            if "Nonsense_Mutation" in set(part[part["Protein Change"] == mut[0]]["Mutation Type"]):
                nons.append(mut[0])
```

```
# Calculate the proportion of samples with mutated CDKN2A, that harbor one of the
# most frequent nonsense mutations you found above.
inds = []
for i in part.index:
    if part.iloc[i]["Protein Change"] in nons:
        inds.append(i)
p = len(set(part.iloc[inds]["Sample ID"])) / len(set(part["Sample ID"])) * 100
p = float("{:.2f}".format(p))
```

Something similar for *ATM* gene.

```
cd ~\python programming\3

import pandas as pd
from collections import Counter

data =
pd.read_csv("driver_mutations_for_204_cancer_genes_includes_pathways_new_dataset_primary_t
umors.csv", index_col=0)
part = data[data.Gene == "ATM"].reset_index().drop(columns="index")

# Sort ATM mutations by their frequency
mf = Counter(list(part["Protein Change"])).most_common()

# Find ATM mutations affecting R337 position.
nons = []
for mut in mf:
    if "R337" in mut[0]:
        nons.append(mut[0])

# Calculate the proportion of samples with mutated ATM, that harbor one of the
# most frequent nonsense mutations you found above.
inds = []
for i in part.index:
    if part.iloc[i]["Protein Change"] in nons:
        inds.append(i)
p = len(set(part.iloc[inds]["Sample ID"])) / len(set(part["Sample ID"])) * 100
p = float("{:.2f}".format(p))

# Find the most frequent Mutation Type in ATM gene. (We have checked that each
# mutation belongs only to one Mutation Type). Due to the presence of more than
# one mutations in ATM gene in some patients, the sum of the percentages calculated
# here exceeds 100 %.
rank = []
for mt in list(set(part["Mutation Type"])):
    z = len(set(part[part["Mutation Type"] == mt]["Sample ID"])) / len(set(part["Sample ID"])) * 100
    z = float("{:.2f}".format(z))
    rank.append((mt, z))
rank = sorted(rank, key=lambda x: x[1], reverse=True)
```

RTK-RAS pathway driver mutations: calculate the proportion of patients harboring driver mutations only in receptor tyrosine kinase – encoding genes of the pathway (all combined, not separately).

```
cd ~\python programming\3

import pandas as pd

data =
pd.read_csv("driver_mutations_for_204_cancer_genes_includes_pathways_new_dataset_primary_t
umors.csv", index_col=0)

rtks = ["EGFR", "ERBB2", "FGFR3", "FLT3", "FGFR2", "ERBB3", "RET", "KIT", "MET",
"ERBB4", "PDGFRA", "ALK", "FGFR1", "NTRK3", "NTRK1", "ROS1", "NTRK2", "IGF1R",
"FGFR4"]

# Isolate only data referring to RTK-RAS pathway alterations (some genes
# may also participate in another pathway).
inds = []
for i in data.index:
    if "RTK_RAS" in data.iloc[i]["Pathways"]:
        inds.append(i)
drtk = data.iloc[inds].reset_index().drop(columns="index")

# Find all different sample IDs of RTK-RAS affected patients, that carry a
# driver mutation in one of the receptor tyrosine kinases of this pathway.
ids = []
for k in drtk.index:
    if drtk.iloc[k].Gene in rtks:
        if drtk.iloc[k]["Sample ID"] not in ids:
            ids.append(drtk.iloc[k]["Sample ID"])

p = len(ids) / len(set(drtk["Sample ID"])) * 100
p = float("{:.2f}".format(p))
```

KRAS

```
cd ~\python programming\3

import pandas as pd
from collections import Counter

data =
pd.read_csv("driver_mutations_for_204_cancer_genes_includes_pathways_new_dataset_primary_t
umors.csv", index_col=0)
part = data[data.Gene == "KRAS"].reset_index().drop(columns="index")

# Sort KRAS mutations by their frequency
```

```
mf = Counter(list(part["Protein Change"])).most_common()

# Sort the 4 most frequently altered KRAS positions by their mutational frequency.
# We 've checked that no other positions start with the same code as the four
# positions of interest (e.g. for G12 there is no G126).
nons = []
for pos in ["G12", "G13", "Q61", "A146"]:
    # "c" counts the affected registries for each position
    c = 0
    for mut in mf:
        if pos in mut[0]:
            c += mut[1]
    nons.append((pos, c))
nons = sorted(nons, key= lambda x: x[1], reverse=True)

# Calculate the proportion of samples with mutated KRAS, that carry an alteration
# in each of the four most frequently mutated positions of the gene.
rank = []
x, y = zip(*nons)
for thesi in x:
    inds = []
    for i in part.index:
        if part.iloc[i]["Protein Change"].startswith(thesi):
            inds.append(i)
    p = len(set(part.iloc[inds]["Sample ID"])) / len(set(part["Sample ID"])) * 100
    p = float("{:.2f}".format(p))
    rank.append((thesi, p))

# Calculate the total proportion of samples with mutated KRAS, that carry an
# alteration in one of the four most frequently mutated positions of the gene. This
# proportion may slightly differ from the sum of the proportions calculated above,
# because some patients may carry more than one of these mutations in KRAS gene.
inds = []
for thesi in x:
    for i in part.index:
        if thesi in part.iloc[i]["Protein Change"]:
            inds.append(i)
p = len(set(part.iloc[inds]["Sample ID"])) / len(set(part["Sample ID"])) * 100
p = float("{:.2f}".format(p))
```

BRAF

```
cd ~\python programming\3

import pandas as pd
from collections import Counter

data =
pd.read_csv("driver_mutations_for_204_cancer_genes_includes_pathways_new_dataset_primary_t
umors.csv", index_col=0)
part = data[data.Gene == "BRAF"].reset_index().drop(columns="index")
```

```
# Sort BRAF mutations by their frequency
mf = Counter(list(part["Protein Change"])).most_common()


# Calculate the proportion of samples bearing BRAF mutations at position V600.
inds = []
for i in part.index:
    if part.iloc[i]["Protein Change"].startswith("V600"):
        inds.append(i)
p = len(set(part.iloc[inds]["Sample ID"])) / len(set(part["Sample ID"])) * 100
p = float("{:.2f}".format(p))

# Isolate BRAF rearrangement partner genes. Only in rearrangement names there
# is the "-" symbol, which separates the participating genes.
x, y = zip(*mf)
partners = []
for mut in x:
    if "-" in mut:
        for par in mut.split("-"):
            if par != "BRAF" and par not in partners:
                partners.append(par)

# Calculate the proportion of samples bearing BRAF rearrangements.
indices = []
for i in part.index:
    if "-" in part.iloc[i]["Protein Change"]:
        indices.append(i)
perc = len(set(part.iloc[indices]["Sample ID"])) / len(set(part["Sample ID"])) * 100
perc = float("{:.2f}".format(perc))
```

NF1

```
cd ~\python programming\3

import pandas as pd
from collections import Counter

data =
pd.read_csv("driver_mutations_for_204_cancer_genes_includes_pathways_new_dataset_primary_t
umors.csv", index_col=0)
part = data[data.Gene == "NF1"].reset_index().drop(columns="index")

# Sort NF1 mutations by their frequency
mf = Counter(list(part["Protein Change"])).most_common()

# Find the most frequent Mutation Type in NF1 gene. (We have checked that each
# mutation belongs only to one Mutation Type). Calculate the occurence of each
# type utilizing mutation registries, not sample IDs, because some patients
```

```
# carry more than 1 mutations in NF1 gene, so using sample IDs the results
# will be onerlapped.
rank = []
for mt in list(set(part["Mutation Type"])):
    z = len(part[part["Mutation Type"] == mt]) / len(part) * 100
    z = float("{:.2f}".format(z))
    rank.append((mt, z))
rank = sorted(rank, key=lambda x: x[1], reverse=True)

# Calculate the occurence of "R2450*" mutation against all somatic NF1 mutations.
# (our data include only somatic and no germline mutations)
inds = []
for i in part.index:
    if part.iloc[i]["Protein Change"] == "R2450*":
        inds.append(i)
p = len(part.iloc[inds]) / len(part) * 100
p = float("{:.2f}".format(p))
```

Lipids metabolism: sort genes of the pathway by the proportion of patients affected (among all patients with driver mutation/s in this pathway). Then, depending on the gene case, make the appropriate calculations needed for the paper.

```
cd ~\python programming\3

import pandas as pd
from collections import Counter

data =
pd.read_csv("driver_mutations_for_204_cancer_genes_includes_pathways_new_dataset_primary_t
umors.csv", index_col=0)

# Isolate only data referring to Lipids pathway alterations (some genes
# may also participate in another pathway).
inds = []
for i in data.index:
    if "Lipid" in data.iloc[i]["Pathways"]:
        inds.append(i)
part = data.iloc[inds].reset_index().drop(columns="index")

# Sort Lipids metabolism's genes by the number of identified driver mutations.
mf = Counter(list(part.Gene)).most_common()
x, y = zip(*mf)

# Find all different sample IDs of Lipid metabolism affected patients, that carry a
# driver mutation in each of the mutated genes of this pathway. Then, calculate
# the corresponding proportion.
rank = []
for gene in x:
    ids = [sid for sid in set(part[part.Gene == gene]["Sample ID"])]
    p = len(ids) / len(set(part["Sample ID"])) * 100
```

```
    p = float("{:.2f}".format(p))
    rank.append((gene, p))

# Isolate the 3 top mutated genes of the Lipids metabolism pathway.
z, h = zip(*rank[:3])
# For each of these genes, sort their mutations by the number of their registries.
rm = []
for gene in z:
    part = data[data.Gene == gene].reset_index().drop(columns="index")
    mf = Counter(list(part["Protein Change"])).most_common()
    rm.append((gene, mf))


# PIK3CA'S RESULTS MANAGEMENT

r1 = Counter(data[data.Gene == "PIK3CA"]["Mutation Type"]).most_common()
pik3ca = data[data.Gene == "PIK3CA"]
# Proportion of missense mutations among all PIK3CA mutations
r1[0][1] / len(pik3ca) *100
# Sum the registries of the 3 most frequent mutations of PIK3CA gene
x, y = zip(*rm[0][1][:3])
sum(y) / len(pik3ca[pik3ca["Mutation Type"] == "Missense_Mutation"]) *100

# PTEN's RESULTS MANAGEMENT

r2 = Counter(data[data.Gene == "PTEN"]["Mutation Type"]).most_common()
pten = data[data.Gene == "PTEN"].reset_index().drop(columns="index")
x, y = zip(*r2)
# Calculate the total proportion of PTEN carriers with missense, frameshift
# indels and nonsense mutations.
inds = []
for i in pten.index:
    if pten.iloc[i]["Mutation Type"] in x[:4]:
        inds.append(i)
p = len(set(pten.iloc[inds]["Sample ID"])) / len(set(pten["Sample ID"])) * 100
p = float("{:.2f}".format(p))

# Sort the first 3 hotspots by their mutational occurence.
rank = []
for mut in ["R130", "R233", "T319"]:
    indices = []
    for i in pten.index:
        if pten.iloc[i]["Protein Change"].startswith(mut):
            indices.append(i)
    p = len(set(pten.iloc[indices]["Sample ID"])) / len(set(pten["Sample ID"])) * 100
    p = float("{:.2f}".format(p))
    rank.append((mut, p))
rank = sorted(rank, key = lambda x : x[1], reverse=True)

# IDH1's RESULTS MANAGEMENT

# Find all IDH1 mutation registries. From rm[2][1] arises that almost all mutants
```

```
# affect R132 position.
x, y = zip(*rm[2][1])
sum(y)
# Calculate the number of patients where these mutations are present (in data
# there is one sample per patient so each Sample ID represents a different patient)
len(set(data[data.Gene == "IDH1"]["Sample ID"]))
```

PI3K/Akt: sort genes of the pathway by the proportion of patients affected (among all patients with driver mutation/s in this pathway). Then, depending on the gene case, make the appropriate calculations needed for the paper.

```
cd ~\python programming\3

import pandas as pd
from collections import Counter

data =
pd.read_csv("driver_mutations_for_204_cancer_genes_includes_pathways_new_dataset_primary_t
umors.csv", index_col=0)

# Isolate mutation registries referring to PI3K/Akt pathway genes (some genes
# also participate in other pathways).
inds = []
for i in data.index:
    if "PI3K_Akt" in data.iloc[i].Pathways:
        inds.append(i)
part = data.iloc[inds].reset_index().drop(columns="index")

# Get an idea of the most frequently mutated genes of the pathway (by counting
# the mutation registries for each of them). However, this is not the best way
# to conclude to the sorted gene list, given that some patients may harbor more
# than one mutation in the same gene (even though this is somewhat rare).
# Sort Lipids metabolism's genes by the number of identified driver mutations.
mf = Counter(part.Gene).most_common()
x, y = zip(*mf)


# Find all different sample IDs of PI3K/Akt affected patients, that carry a
# driver mutation in each of the mutated genes of this pathway. Then, calculate
# the corresponding proportion.
rank = []
for gene in x:
    ids = [sid for sid in set(part[part.Gene == gene]["Sample ID"])]
    p = len(ids) / len(set(part["Sample ID"])) * 100
    p = float("{:.2f}".format(p))
    rank.append((gene, p))
rank = sorted(rank, key=lambda x : x[1], reverse=True)

# Calculate the proportion of PI3K/Akt-deregulated patients harboring mutations
# in at least one of the three top mutated genes of the pathway (PIK3CA, PTEN
```

```
# and PIK3R1)
unique = []
for gene in x[:3]:
    ids = [sid for sid in set(part[part.Gene == gene]["Sample ID"])]
    for patient in ids:
        if patient not in unique:
            unique.append(patient)
t3 = len(unique) / len(set(part["Sample ID"])) * 100
t3 = float("{:.2f}".format(t3))

# Calculate the proportion of PI3K/Akt-deregulated patients harboring mutations
# in at least two of the three top mutated genes of the pathway (PIK3CA, PTEN
# and PIK3R1)
two = 0
three = 0
for patient in unique:
    score = 0
    mutg = list(part[part["Sample ID"] == patient].Gene)
    for top in x[:3]:
        if top in mutg:
            score += 1
    if score == 2:
        two += 1
    elif score == 3:
        three += 1
two_and_three = (two + three) / len(set(part["Sample ID"])) * 100
two_and_three = float("{:.2f}".format(two_and_three))

# PIK3R1's RESULTS MANAGEMENT
pik3r1 = part[part.Gene == "PIK3R1"].reset_index().drop(columns="index")
pch = Counter(pik3r1["Protein Change"]).most_common()
ch, fr = zip(*pch)

# Sort protein changes of PIK3R1 by their occurrence rate
rank = []
for mut in ch:
    indices = []
    for i in pik3r1.index:
        if pik3r1.iloc[i]["Protein Change"] == mut:
            indices.append(i)
    p = len(set(pik3r1.iloc[indices]["Sample ID"])) / len(set(pik3r1["Sample ID"])) * 100
    p = float("{:.2f}".format(p))
    rank.append((mut, p))
rank = sorted(rank, key = lambda x : x[1], reverse=True)
```

Ubiquitin pathway. Depending on the gene case, make the appropriate calculations needed for the paper.

```
cd ~\python programming\3

import pandas as pd
from collections import Counter
```

```
data =
pd.read_csv("driver_mutations_for_204_cancer_genes_includes_pathways_new_dataset_primary_t
umors.csv", index_col=0)

# Isolate mutation registries referring to ubiquitination or deubiquitination
# pathways' genes (some genes also participate in other pathways).
inds = []
for i in data.index:
    if "Dubq" in data.iloc[i].Pathways or "Ubq" in data.iloc[i].Pathways:
        inds.append(i)
part = data.iloc[inds].reset_index().drop(columns="index")

rank = []
for gene in set(part.Gene):
    ids = [sid for sid in set(part[part.Gene == gene]["Sample ID"])]
    p = len(ids) / len(set(part["Sample ID"])) * 100
    p = float("{:.2f}".format(p))
    rank.append((gene, p))
rank = sorted(rank, key=lambda x : x[1], reverse=True)


# FBXW7's RESULTS MANAGEMENT
fbxw7 = part[part.Gene == "FBXW7"].reset_index().drop(columns="index")
pch = Counter(fbxw7["Protein Change"]).most_common()
ch, fr = zip(*pch)

# Sort protein changes of FBXW7 by their occurrence rate
rank = []
for mut in ch:
    indices = []
    for i in fbxw7.index:
        if fbxw7.iloc[i]["Protein Change"] == mut:
            indices.append(i)
    p = len(set(fbxw7.iloc[indices]["Sample ID"])) / len(set(fbxw7["Sample ID"])) * 100
    p = float("{:.2f}".format(p))
    rank.append((mut, p))
rank = sorted(rank, key = lambda x : x[1], reverse=True)

# Calculate the proportion of samples bearing FBXW7 mutations at positions R465,
# R505, R479 and all together.
rankpos = []
unique = []
for pos in ["R465", "R505", "R479"]:
    sids = []
    for i in fbxw7.index:
        if fbxw7.iloc[i]["Protein Change"].startswith(pos):
            sids.append(fbxw7.iloc[i]["Sample ID"])
            if fbxw7.iloc[i]["Sample ID"] not in unique:
                unique.append(fbxw7.iloc[i]["Sample ID"])
    p = len(set(sids)) / len(set(fbxw7["Sample ID"])) * 100
    p = float("{:.2f}".format(p))
```

```
    rankpos.append((pos, p))
p = len(set(unique)) / len(set(fbxw7["Sample ID"])) * 100
p = float("{:.2f}".format(p))
rankpos.append(("ALL", p))

# EP300's RESULTS MANAGEMENT
ep300 = part[part.Gene == "EP300"].reset_index().drop(columns="index")
pch = Counter(ep300["Protein Change"]).most_common()
ch, fr = zip(*pch)

# Sort protein changes of EP300 by their occurrence rate
rank = []
for mut in ch:
    indices = []
    for i in ep300.index:
        if ep300.iloc[i]["Protein Change"] == mut:
            indices.append(i)
    p = len(set(ep300.iloc[indices]["Sample ID"])) / len(set(ep300["Sample ID"])) * 100
    p = float("{:.2f}".format(p))
    rank.append((mut, p))
rank = sorted(rank, key = lambda x : x[1], reverse=True)

# Get an idea of the frequency of each mutation type affecting EP300 gene.
Counter(ep300["Mutation Type"]).most_common()

# CREBBP's RESULTS MANAGEMENT
crebbp = part[part.Gene == "CREBBP"].reset_index().drop(columns="index")
pch = Counter(crebbp["Protein Change"]).most_common()
ch, fr = zip(*pch)

# Sort protein changes of CREBBP by their occurrence rate
rank = []
for mut in ch:
    indices = []
    for i in crebbp.index:
        if crebbp.iloc[i]["Protein Change"] == mut:
            indices.append(i)
    p = len(set(crebbp.iloc[indices]["Sample ID"])) / len(set(crebbp["Sample ID"])) * 100
    p = float("{:.2f}".format(p))
    rank.append((mut, p))
rank = sorted(rank, key = lambda x : x[1], reverse=True)

# Get an idea of the frequency of each mutation type affecting crebbp gene.
Counter(crebbp["Mutation Type"]).most_common()
```

WNT/b catenin pathway. Depending on the gene case, make the appropriate calculations needed for the paper.

```
cd ~\python programming\3
```

```
import pandas as pd
from collections import Counter
import re

data =
pd.read_csv("driver_mutations_for_204_cancer_genes_includes_pathways_new_dataset_primary_t
umors.csv", index_col=0)

# Isolate mutation registries referring to WNT/b catenin pathways' genes (some
# genes also participate in other pathways).
inds = []
for i in data.index:
    if "b_catenin_Wnt" in data.iloc[i].Pathways:
        inds.append(i)
part = data.iloc[inds].reset_index().drop(columns="index")

rank = []
for gene in set(part.Gene):
    ids = [sid for sid in set(part[part.Gene == gene]["Sample ID"])]
    p = len(ids) / len(set(part["Sample ID"])) * 100
    p = float("{:.2f}".format(p))
    rank.append((gene, p))
rank = sorted(rank, key=lambda x : x[1], reverse=True)

# APC's RESULTS MANAGEMENT
apc = part[part.Gene == "APC"].reset_index().drop(columns="index")
pch = Counter(apc["Protein Change"]).most_common()
ch, fr = zip(*pch)

# Sort protein changes of APC by their occurrence rate
rank = []
for mut in ch:
    indices = []
    for i in apc.index:
        if apc.iloc[i]["Protein Change"] == mut:
            indices.append(i)
    p = len(set(apc.iloc[indices]["Sample ID"])) / len(set(apc["Sample ID"])) * 100
    p = float("{:.2f}".format(p))
    rank.append((mut, p))
rank = sorted(rank, key = lambda x : x[1], reverse=True)

# Get an idea of the frequency of each mutation type affecting APC gene.
Counter(apc["Mutation Type"]).most_common()

# Find the most frequent Mutation Type in APC gene. (We have checked that each
# mutation belongs only to one Mutation Type). Due to the presence of more than
# one mutations in APC gene in some patients, the sum of the percentages calculated
# here exceeds 100 %.
rankmt = []
for mt in list(set(part["Mutation Type"])):
    z = len(set(part[part["Mutation Type"] == mt]["Sample ID"])) / len(set(part["Sample ID"])) * 100
    z = float("{:.2f}".format(z))
```

```
    rankmt.append((mt, z))
rankmt = sorted(rankmt, key=lambda x: x[1], reverse=True)

# CTNNB1's RESULTS MANAGEMENT
ctnnb1 = part[part.Gene == "CTNNB1"].reset_index().drop(columns="index")
pch = Counter(ctnnb1["Protein Change"]).most_common()
ch, fr = zip(*pch)

# Sort protein changes of CTNNB1 by their occurrence rate
rank = []
for mut in ch:
    indices = []
    for i in ctnnb1.index:
        if ctnnb1.iloc[i]["Protein Change"] == mut:
            indices.append(i)
    p = len(set(ctnnb1.iloc[indices]["Sample ID"])) / len(set(ctnnb1["Sample ID"])) * 100
    p = float("{:.2f}".format(p))
    rank.append((mut, p))
rank = sorted(rank, key = lambda x : x[1], reverse=True)

# Find all substitution positions of CTNNB1 gene. We have checked that missense
# mutations of this gene happen in two- or three-digit positions, so the
# alterations consist of up to 5 characters. Contrariwise, protein changes that
# belong to other mutation types (here, only deletions) consist of at least 6
# characters (this is the case for the mutations we have identified in CTNNB1)
positions = []
for pc in ch:
    if len(pc) <= 5:
        if re.findall("[0-9]+",pc)[0] not in positions:
            positions.append(re.findall("[0-9]+",pc)[0])

# Calculate the proportion of samples bearing CTNNB1 mutations at each
# substitution position, as well as the total proportion of the 6 most prevalent
# (in our results, these are 33, 37, 32, 45, 41 and 34).
rankpos = []
unique = []
for pos in positions:
    sids = []
    # We have to avoid overlapped counting. For example, when counting Sample IDs
    # harboring substitutions at position 33, we don't want to include Sample IDs
    # that harbor substitution at position 335 (which includes term 33).
    if len(pos) == 2:
        for i in ctnnb1.index:
            if len(ctnnb1.iloc[i]["Protein Change"]) == 4 and pos in ctnnb1.iloc[i]["Protein Change"]:
                sids.append(ctnnb1.iloc[i]["Sample ID"])
    elif len(pos) == 3:
        for i in ctnnb1.index:
            if len(ctnnb1.iloc[i]["Protein Change"]) == 5 and pos in ctnnb1.iloc[i]["Protein Change"]:
                sids.append(ctnnb1.iloc[i]["Sample ID"])

    if pos in ["33", "37", "32", "45", "41", "34"]:
        for s in sids:
```

```
        if s not in unique:
            unique.append(s)
    p = len(set(sids)) / len(set(ctnnb1["Sample ID"])) * 100
    p = float("{:.2f}".format(p))
    rankpos.append((pos, p))
p = len(set(unique)) / len(set(ctnnb1["Sample ID"])) * 100
p = float("{:.2f}".format(p))
rankpos.append(("Hottest 6 positions", p))
rankpos = sorted(rankpos, key= lambda x : x[1], reverse=True)

# Get an idea of the frequency of each mutation type affecting CTNNB1 gene.
Counter(ctnnb1["Mutation Type"]).most_common()


# RNF43's RESULTS MANAGEMENT
rnf43 = part[part.Gene == "RNF43"].reset_index().drop(columns="index")
pch = Counter(rnf43["Protein Change"]).most_common()
ch, fr = zip(*pch)

# Sort protein changes of RNF43 by their occurrence rate
rank = []
for mut in ch:
    indices = []
    for i in rnf43.index:
        if rnf43.iloc[i]["Protein Change"] == mut:
            indices.append(i)
    p = len(set(rnf43.iloc[indices]["Sample ID"])) / len(set(rnf43["Sample ID"])) * 100
    p = float("{:.2f}".format(p))
    rank.append((mut, p))
rank = sorted(rank, key = lambda x : x[1], reverse=True)

# Find the most frequent Mutation Type in RNF43 gene. (We have checked that each
# mutation belongs only to one Mutation Type). Due to the presence of more than
# one mutations in RNF43 gene in some patients, the sum of the percentages calculated
# here may exceed 100 %.
rankmt = []
for mt in list(set(rnf43["Mutation Type"])):
    z = len(set(rnf43[rnf43["Mutation Type"] == mt]["Sample ID"])) / len(set(rnf43["Sample ID"])) *
100
    z = float("{:.2f}".format(z))
    rankmt.append((mt, z))
rankmt = sorted(rankmt, key=lambda x: x[1], reverse=True)
```

Cell cycle pathway. Depending on the gene case, make the appropriate calculations needed for the paper.

```
import os
os.chdir("~\\python programming\\3")

import pandas as pd
from collections import Counter
```

```
import re

data =
pd.read_csv("driver_mutations_for_204_cancer_genes_includes_pathways_new_dataset_primary_t
umors.csv", index_col=0)

# Isolate mutation registries referring to cell cycle pathways' genes (some
# genes also participate in other pathways).
inds = []
for i in data.index:
    if "cell_cycle" in data.iloc[i].Pathways:
        inds.append(i)
part = data.iloc[inds].reset_index().drop(columns="index")

# RB1's RESULTS MANAGEMENT
rb1 = part[part.Gene == "RB1"].reset_index().drop(columns="index")
pch = Counter(rb1["Protein Change"]).most_common()
ch, fr = zip(*pch)

# Sort protein changes of RB1 by their occurrence rate
rank = []
for mut in ch:
    indices = []
    for i in rb1.index:
        if rb1.iloc[i]["Protein Change"] == mut:
            indices.append(i)
    p = len(set(rb1.iloc[indices]["Sample ID"])) / len(set(rb1["Sample ID"])) * 100
    p = float("{:.2f}".format(p))
    rank.append((mut, p))
rank = sorted(rank, key = lambda x : x[1], reverse=True)

# Find the most frequent Mutation Type in RB1 gene. (We have checked that each
# mutation belongs only to one Mutation Type). Due to the presence of more than
# one mutations in RB1 gene in some patients, the sum of the percentages calculated
# here may exceed 100 %.
rankmt = []
for mt in list(set(rb1["Mutation Type"])):
    z = len(set(rb1[rb1["Mutation Type"] == mt]["Sample ID"])) / len(set(rb1["Sample ID"])) * 100
    z = float("{:.2f}".format(z))
    rankmt.append((mt, z))
rankmt = sorted(rankmt, key=lambda x: x[1], reverse=True)

# Find all non-rearrangement (non-fusion) positions of RB1 gene. Fusion names
# are the only ones including a "-" in them. We exclude them because we want to
# rank specific mutational positions for their alteration rate (fusion names
# just include the implicated gene names).
positions = []
for pc in ch:
    if "-" not in pc:
        if re.findall("[0-9]+",pc)[0] not in positions:
            positions.append(re.findall("[0-9]+",pc)[0])
```

```
# Calculate the proportion of samples bearing RB1 mutations at each of the above
# positions.
rankpos = []
unique = []
for pos in positions:
    sids = []
    # We have to avoid overlapped counting. For example, when counting Sample IDs
    # harboring substitutions at position 33, we don't want to include Sample IDs
    # that harbor substitution at position 335 (which includes term 33).
    for i in rb1.index:
        if "-" in rb1.iloc[i]["Protein Change"]:
            continue
        else:
            if int(pos) == int(re.findall("[0-9]+", rb1.iloc[i]["Protein Change"])[0]):
                sids.append(rb1.iloc[i]["Sample ID"])
    p = len(set(sids)) / len(set(rb1["Sample ID"])) * 100
    p = float("{:.2f}".format(p))
    rankpos.append((pos, p))
rankpos = sorted(rankpos, key= lambda x : x[1], reverse=True)

# CDKN1A's RESULTS MANAGEMENT
cdkn1a = part[part.Gene == "CDKN1A"].reset_index().drop(columns="index")
pch = Counter(cdkn1a["Protein Change"]).most_common()
ch, fr = zip(*pch)

# Sort protein changes of CDKN1A by their occurrence rate
rank = []
for mut in ch:
    indices = []
    for i in cdkn1a.index:
        if cdkn1a.iloc[i]["Protein Change"] == mut:
            indices.append(i)
    p = len(set(cdkn1a.iloc[indices]["Sample ID"])) / len(set(cdkn1a["Sample ID"])) * 100
    p = float("{:.2f}".format(p))
    rank.append((mut, p))
rank = sorted(rank, key = lambda x : x[1], reverse=True)

# Find the most frequent Mutation Type in CDKN1A gene. (We have checked that each
# mutation belongs only to one Mutation Type). Due to the presence of more than
# one mutations in CDKN1A gene in some patients, the sum of the percentages calculated
# here may exceed 100 %.
rankmt = []
for mt in list(set(cdkn1a["Mutation Type"])):
    z = len(set(cdkn1a[cdkn1a["Mutation Type"] == mt]["Sample ID"])) / len(set(cdkn1a["Sample
ID"])) * 100
    z = float("{:.2f}".format(z))
    rankmt.append((mt, z))
rankmt = sorted(rankmt, key=lambda x: x[1], reverse=True)

# Check the cancer types where CDKN1A mutations occur and their rate
Counter(cdkn1a["Cancer Type"]).most_common()
```

HDR pathway. Depending on the gene case, make the appropriate calculations needed for the paper.

```
import os
os.chdir("~\\python programming\\3")

import pandas as pd
from collections import Counter
import re

data =
pd.read_csv("driver_mutations_for_204_cancer_genes_includes_pathways_new_dataset_primary_t
umors.csv", index_col=0)

# Isolate mutation registries referring to HDR pathways' genes (some
# genes also participate in other pathways).
inds = []
for i in data.index:
    if "HDR" in data.iloc[i].Pathways:
        inds.append(i)
part = data.iloc[inds].reset_index().drop(columns="index")

# BRCA2's RESULTS MANAGEMENT
brca2 = part[part.Gene == "BRCA2"].reset_index().drop(columns="index")
pch = Counter(brca2["Protein Change"]).most_common()
ch, fr = zip(*pch)

# Sort protein changes of BRCA2 by their occurrence rate
rank = []
for mut in ch:
    indices = []
    for i in brca2.index:
        if brca2.iloc[i]["Protein Change"] == mut:
            indices.append(i)
    p = len(set(brca2.iloc[indices]["Sample ID"])) / len(set(brca2["Sample ID"])) * 100
    p = float("{:.2f}".format(p))
    rank.append((mut, p))
rank = sorted(rank, key = lambda x : x[1], reverse=True)

# Find the most frequent Mutation Type in BRCA2 gene. (We have checked that each
# mutation belongs only to one Mutation Type). Due to the presence of more than
# one mutations in BRCA2 gene in some patients, the sum of the percentages calculated
# here may exceed 100 %.
rankmt = []
for mt in list(set(brca2["Mutation Type"])):
    z = len(set(brca2[brca2["Mutation Type"] == mt]["Sample ID"])) / len(set(brca2["Sample ID"]))
* 100
    z = float("{:.2f}".format(z))
    rankmt.append((mt, z))
rankmt = sorted(rankmt, key=lambda x: x[1], reverse=True)
```

```
# Check the cancer types where BRCA2 mutations occur and their rate
Counter(brca2["Cancer Type"]).most_common()

# Find all non-rearrangement (non-fusion) positions of BRCA2 gene. Fusion names
# are the only ones including a "-" in them. We exclude them because we want to
# rank specific mutational positions for their alteration rate (fusion names
# just include the implicated gene names).
positions = []
for pc in ch:
   if "-" not in pc:
      if re.findall("[0-9]+",pc)[0] not in positions:
         positions.append(re.findall("[0-9]+",pc)[0])

# Calculate the proportion of samples bearing BRCA2 mutations at each of the above
# positions.
rankpos = []
unique = []
for pos in positions:
   sids = []
   # We have to avoid overlapped counting. For example, when counting Sample IDs
   # harboring substitutions at position 33, we don't want to include Sample IDs
   # that harbor substitution at position 335 (which includes term 33).
   for i in brca2.index:
      if "-" in brca2.iloc[i]["Protein Change"]:
         continue
      else:
         if int(pos) == int(re.findall("[0-9]+", brca2.iloc[i]["Protein Change"])[0]):
            sids.append(brca2.iloc[i]["Sample ID"])
   p = len(set(sids)) / len(set(brca2["Sample ID"])) * 100
   p = float("{:.2f}".format(p))
   rankpos.append((pos, p))
rankpos = sorted(rankpos, key= lambda x : x[1], reverse=True)

# Find all nonsense mutation positions of BRCA2 gene.
positions = []
for pc in set(brca2[brca2["Mutation Type"] == "Nonsense_Mutation"]["Protein Change"]):
   if re.findall("[0-9]+",pc)[0] not in positions:
      positions.append(re.findall("[0-9]+",pc)[0])

# Find how many of them lose the C-terminal ~1000 amino acid residues. BRCA2
# protein has 3418 amino acids (UniProtKB - P51587) and its DNA-binding domain
# starts from position 2479 (PMID: 25447315). Compare the result with
# len(positions).
c = 0
for p in positions:
   if int(p)<2479:
      c+=1


# BRCA1's RESULTS MANAGEMENT
brca1 = part[part.Gene == "BRCA1"].reset_index().drop(columns="index")
pch = Counter(brca1["Protein Change"]).most_common()
```

```
ch, fr = zip(*pch)

# Sort protein changes of BRCA1 by their occurrence rate
rank = []
for mut in ch:
    indices = []
    for i in brca1.index:
        if brca1.iloc[i]["Protein Change"] == mut:
            indices.append(i)
    p = len(set(brca1.iloc[indices]["Sample ID"])) / len(set(brca1["Sample ID"])) * 100
    p = float("{:.2f}".format(p))
    rank.append((mut, p))
rank = sorted(rank, key = lambda x : x[1], reverse=True)

# Find the most frequent Mutation Type in BRCA1 gene. (We have checked that each
# mutation belongs only to one Mutation Type). Due to the presence of more than
# one mutations in BRCA1 gene in some patients, the sum of the percentages calculated
# here may exceed 100 %.
rankmt = []
for mt in list(set(brca1["Mutation Type"])):
    z = len(set(brca1[brca1["Mutation Type"] == mt]["Sample ID"])) / len(set(brca1["Sample ID"]))
* 100
    z = float("{:.2f}".format(z))
    rankmt.append((mt, z))
rankmt = sorted(rankmt, key=lambda x: x[1], reverse=True)


# TP53BP1's RESULTS MANAGEMENT
tp53bp1 = part[part.Gene == "TP53BP1"].reset_index().drop(columns="index")
pch = Counter(tp53bp1["Protein Change"]).most_common()
ch, fr = zip(*pch)

# Sort protein changes of TP53BP1 by their occurrence rate
rank = []
for mut in ch:
    indices = []
    for i in tp53bp1.index:
        if tp53bp1.iloc[i]["Protein Change"] == mut:
            indices.append(i)
    p = len(set(tp53bp1.iloc[indices]["Sample ID"])) / len(set(tp53bp1["Sample ID"])) * 100
    p = float("{:.2f}".format(p))
    rank.append((mut, p))
rank = sorted(rank, key = lambda x : x[1], reverse=True)

# Find the most frequent Mutation Type in TP53BP1 gene. (We have checked that each
# mutation belongs only to one Mutation Type). Due to the presence of more than
# one mutations in TP53BP1 gene in some patients, the sum of the percentages calculated
# here may exceed 100 %.
rankmt = []
for mt in list(set(tp53bp1["Mutation Type"])):
    z = len(set(tp53bp1[tp53bp1["Mutation Type"] == mt]["Sample ID"])) / len(set(tp53bp1["Sample
ID"])) * 100
```

```
    z = float("{:.2f}".format(z))
    rankmt.append((mt, z))
rankmt = sorted(rankmt, key=lambda x: x[1], reverse=True)

# Calculate the proportion of TP53BP1-affected patients harboring nonsense or
# frameshift deletion.
sids = []
for selmt in [rankmt[0][0], rankmt[1][0]]:
    sids = sids + list(tp53bp1[tp53bp1["Mutation Type"] == selmt]["Sample ID"])
x = len(set(sids)) / len(set(tp53bp1["Sample ID"])) * 100
x = float("{:.2f}".format(x))
```

Splicing pathway. Depending on the gene case, make the appropriate calculations needed for the paper.

```
import os
os.chdir("~\\python programming\\3")

import pandas as pd
from collections import Counter
import re

data =
pd.read_csv("driver_mutations_for_204_cancer_genes_includes_pathways_new_dataset_primary_t
umors.csv", index_col=0)

# Isolate mutation registries referring to HDR pathways' genes (some
# genes also participate in other pathways).
inds = []
for i in data.index:
    if "Spliceosome" in data.iloc[i].Pathways:
        inds.append(i)
part = data.iloc[inds].reset_index().drop(columns="index")

# SPEN's RESULTS MANAGEMENT
spen = part[part.Gene == "SPEN"].reset_index().drop(columns="index")
pch = Counter(spen["Protein Change"]).most_common()
ch, fr = zip(*pch)

# Sort protein changes of SPEN by their occurrence rate
rank = []
for mut in ch:
    indices = []
    for i in spen.index:
        if spen.iloc[i]["Protein Change"] == mut:
            indices.append(i)
    p = len(set(spen.iloc[indices]["Sample ID"])) / len(set(spen["Sample ID"])) * 100
    p = float("{:.2f}".format(p))
    rank.append((mut, p))
rank = sorted(rank, key = lambda x : x[1], reverse=True)
```

```
# Find the most frequent Mutation Type in SPEN gene. (We have checked that each
# mutation belongs only to one Mutation Type). Due to the presence of more than
# one mutations in SPEN gene in some patients, the sum of the percentages calculated
# here may exceed 100 %.
rankmt = []
for mt in list(set(spen["Mutation Type"])):
    z = len(set(spen[spen["Mutation Type"] == mt]["Sample ID"])) / len(set(spen["Sample ID"])) *
100
    z = float("{:.2f}".format(z))
    rankmt.append((mt, z))
rankmt = sorted(rankmt, key=lambda x: x[1], reverse=True)

# Calculate the proportion of SPEN-affected patients harboring nonsense or
# frameshift deletion.
sids = []
for selmt in [rankmt[0][0], rankmt[1][0]]:
    sids = sids + list(spen[spen["Mutation Type"] == selmt]["Sample ID"])
x = len(set(sids)) / len(set(spen["Sample ID"])) * 100
x = float("{:.2f}".format(x))

# CDK12's RESULTS MANAGEMENT
cdk12 = part[part.Gene == "CDK12"].reset_index().drop(columns="index")
pch = Counter(cdk12["Protein Change"]).most_common()
ch, fr = zip(*pch)

# Sort protein changes of CDK12 by their occurrence rate
rank = []
for mut in ch:
    indices = []
    for i in cdk12.index:
        if cdk12.iloc[i]["Protein Change"] == mut:
            indices.append(i)
    p = len(set(cdk12.iloc[indices]["Sample ID"])) / len(set(cdk12["Sample ID"])) * 100
    p = float("{:.2f}".format(p))
    rank.append((mut, p))
rank = sorted(rank, key = lambda x : x[1], reverse=True)

# Find the most frequent Mutation Type in CDK12 gene. (We have checked that each
# mutation belongs only to one Mutation Type). Due to the presence of more than
# one mutations in CDK12 gene in some patients, the sum of the percentages calculated
# here may exceed 100 %.
rankmt = []
for mt in list(set(cdk12["Mutation Type"])):
    z = len(set(cdk12[cdk12["Mutation Type"] == mt]["Sample ID"])) / len(set(cdk12["Sample
ID"])) * 100
    z = float("{:.2f}".format(z))
    rankmt.append((mt, z))
rankmt = sorted(rankmt, key=lambda x: x[1], reverse=True)

# Calculate the proportion of CDK12-affected patients harboring mutation of
# one of the 3 most frequent mutation types of this gene (fusion, frameshift
# deletion or nonsense mutation).
```

```
sids = []
for i in range(3):
    sids = sids + list(cdk12[cdk12["Mutation Type"] == rankmt[i][0]]["Sample ID"])
x = len(set(sids)) / len(set(cdk12["Sample ID"])) * 100
x = float("{:.2f}".format(x))

# FUBP1's RESULTS MANAGEMENT
fubp1 = part[part.Gene == "FUBP1"].reset_index().drop(columns="index")
pch = Counter(fubp1["Protein Change"]).most_common()
ch, fr = zip(*pch)

# Sort protein changes of FUBP1 by their occurrence rate
rank = []
for mut in ch:
    indices = []
    for i in fubp1.index:
        if fubp1.iloc[i]["Protein Change"] == mut:
            indices.append(i)
    p = len(set(fubp1.iloc[indices]["Sample ID"])) / len(set(fubp1["Sample ID"])) * 100
    p = float("{:.2f}".format(p))
    rank.append((mut, p))
rank = sorted(rank, key = lambda x : x[1], reverse=True)

# Find the most frequent Mutation Type in FUBP1 gene. (We have checked that each
# mutation belongs only to one Mutation Type). Due to the presence of more than
# one mutations in FUBP1 gene in some patients, the sum of the percentages calculated
# here may exceed 100 %.
rankmt = []
for mt in list(set(fubp1["Mutation Type"])):
    z = len(set(fubp1[fubp1["Mutation Type"] == mt]["Sample ID"])) / len(set(fubp1["Sample ID"]))
* 100
    z = float("{:.2f}".format(z))
    rankmt.append((mt, z))
rankmt = sorted(rankmt, key=lambda x: x[1], reverse=True)

# Find all non-rearrangement (non-fusion) positions of FUBP1 gene. Fusion names
# are the only ones including a "-" in them. We exclude them because we want to
# rank specific mutational positions for their alteration rate (fusion names
# just include the implicated gene names).
positions = []
for pc in ch:
    if "-" not in pc:
        if re.findall("[0-9]+",pc)[0] not in positions:
            positions.append(re.findall("[0-9]+",pc)[0])

# Calculate the proportion of samples bearing FUBP1 mutations at each of the above
# positions.
rankpos = []
unique = []
for pos in positions:
    sids = []
    # We have to avoid overlapped counting. For example, when counting Sample IDs
```

```
    # harboring substitutions at position 33, we don't want to include Sample IDs
    # that harbor substitution at position 335 (which includes term 33).
    for i in fubp1.index:
        if "-" in fubp1.iloc[i]["Protein Change"]:
            continue
        else:
            if int(pos) == int(re.findall("[0-9]+", fubp1.iloc[i]["Protein Change"])[0]):
                sids.append(fubp1.iloc[i]["Sample ID"])
    p = len(set(sids)) / len(set(fubp1["Sample ID"])) * 100
    p = float("{:.2f}".format(p))
    rankpos.append((pos, p))
rankpos = sorted(rankpos, key= lambda x : x[1], reverse=True)

# Find all the different missense mutations of FUBP1 gene. We found that among
# the 96 FUBP1-affected patients, the only missense mutation was R340C !
set(fubp1[fubp1["Mutation Type"] == "Missense_Mutation"]["Protein Change"])
len(set(fubp1["Sample ID"]))
```

DISCUSSION

```
import os
os.chdir("~\\python programming\\3")
import pandas as pd

data =
pd.read_csv("driver_mutations_for_204_cancer_genes_includes_pathways_new_dataset_primary_t
umors.csv", index_col=0)
combinations = []
sids = list(set(data[data["Cancer Type"] == "Mucinous Adenocarcinoma of the Colon and
Rectum"]["Sample ID"]))
for sid in sids:
    # Create a string containing all the pathways where the altered genes
    # of each patient participate in.
    paths = []
    for pathlab in list(set(data[data["Sample ID"] == sid]["Pathways"])):
        for part in pathlab.split(", "):
            if part not in paths:
                paths.append(part)
    paths.sort()
    paths = " ".join(paths)
    combinations.append(paths)
Counter(combinations).most_common()

# Check how many of the 55 Mucinous Adenocarcinoma of the Colon and Rectum
# patients, harbor driver somatic alterations in the three most frequently
# mutated pathways of this cancer type simultaneously !
c = 0
for comb in combinations:
```

```
    if "RTK_RAS" in comb and "b_catenin_Wnt" in comb and "Lipid" in comb:
        c += 1

import os
os.chdir("~\\python programming\\3")
import pandas as pd
from collections import Counter

data =
pd.read_csv("driver_mutations_for_204_cancer_genes_includes_pathways_new_dataset_primary_t
umors.csv", index_col=0)
g = data[data.Gene == "CDKN1A"]

# Check how often Q10* and M38Nfs*10 happen in each cancer type (we are
# particularly interested in Bladder Urothelial Carcinoma)
Counter(list(g[g["Protein Change"] == "Q10*"]["Cancer Type"])).most_common()
Counter(list(g[g["Protein Change"] == "M38Nfs*10"]["Cancer Type"])).most_common()


fubp1 = data[data.Gene == "FUBP1"]
# Calculate the proportion of FUBP1-affected patients harboring at least one of
# the following mutations: S11Lfs*43, R430C, I301Yfs*22, I301Nfs*22 and I301Nfs*4
sids = []
for i in ["S11Lfs*43", "R430C", "I301Yfs*22", "I301Nfs*22","I301Nfs*4"]:
    sids = sids + list(fubp1[fubp1["Protein Change"] == i]["Sample ID"])
x = len(set(sids)) / len(set(fubp1["Sample ID"])) * 100
x = float("{:.2f}".format(x))


import os
os.chdir("~\\python programming\\3")
import pandas as pd

data =
pd.read_csv("driver_mutations_for_204_cancer_genes_includes_pathways_new_dataset_primary_t
umors.csv", index_col=0)

sids = list(set(data[data.Gene == "TP53BP1"]["Sample ID"]))
mutexc = 0
# Check the TP53BP1-affected patients for mutual exclusivity between TP53BP1
# and BRCA1/2 driver mutations.
for s in sids:
    x = set(data[data["Sample ID"] == s].Gene)
    if "BRCA2" not in x and "BRCA1" not in x:
        mutexc += 1
p = mutexc / len(sids) * 100
```
