# Supplementary material for: Targeting Oncogenic Pathways in the Era of Personalized Oncology: A Systemic Analysis Reveals Highly Mutated Signaling Pathways in Cancer Patients and Potential Therapeutic Targets
Source: Cancers (Basel). 2022 Jan 28;14(3):664. doi: 10.3390/cancers14030664 (PMC8833388; doi:10.3390/cancers14030664)
Supplement: Supplementary file 1 [file cancers-14-00664-s001.zip › Supplementary Material S2 (Query for searching for driver events of the genes of interest using cBioPortal Onco Query Language, OQL).pdf]

PDGFRA: DRIVER,FBXO11: DRIVER,FANCF: DRIVER,SPEN: DRIVER,DKK3: DRIVER,TRAF5: DRIVER,ERCC3: DRIVER,PALB2: DRIVER,USP8: DRIVER,CCNE1: DRIVER,RAD51B: DRIVER,GNB1: DRIVER,XRCC2: DRIVER,ERRFI1: DRIVER,PIK3R1: DRIVER,MET: DRIVER,RICTOR: DRIVER,E2F3: DRIVER,CREBBP: DRIVER,CACNA1D: DRIVER,PARP1: DRIVER,MLH1: DRIVER,NOTCH4: DRIVER,NTRK1: DRIVER,FGFR3: DRIVER,CDH1: DRIVER,CBLC: DRIVER,BRCA2: DRIVER,MYCL: DRIVER,BRAF: DRIVER,USP6: DRIVER,RAF1: DRIVER,ERBB3: DRIVER,CDKN2B: DRIVER,U2AF2: DRIVER,MSH6: DRIVER,FH: DRIVER,FYN: DRIVER,PRKACA: DRIVER,YAP1: DRIVER,CD36: DRIVER,CAD: DRIVER,RPTOR: DRIVER,ROS1: DRIVER,CLTC: DRIVER,INPP5D: DRIVER,MYC: DRIVER,INPPL1: DRIVER,SRSF2: DRIVER,ACVR2A: DRIVER,RAD51C: DRIVER,PER1: DRIVER,SDHB: DRIVER,PTPN11: DRIVER,TRIM27: DRIVER,MSH3: DRIVER,EXT1: DRIVER,NOTCH3: DRIVER,PIK3R3: DRIVER,PIK3CG: DRIVER,FBXW7: DRIVER,AURKA: DRIVER,CYP19A1: DRIVER,PDK1: DRIVER,RECQL: DRIVER,CD70: DRIVER,TP53: DRIVER,MAP2K1: DRIVER,XIAP: DRIVER,SMARCA4: DRIVER,JAK2: DRIVER,LRP5: DRIVER,SMAD3: DRIVER,PTPN13: DRIVER,RECQL4: DRIVER,PML: DRIVER,ERBB4: DRIVER,CDK6: DRIVER,KIT: DRIVER,PSIP1: DRIVER,CHEK2: DRIVER,RMI2: DRIVER,TNFRSF14: DRIVER,CDKN2C: DRIVER,SOCS2: DRIVER,ERCC2: DRIVER,TCEA1: DRIVER,TSC1: DRIVER,CCNB1IP1: DRIVER,MYCN: DRIVER,CTLA4: DRIVER,XPC: DRIVER,ATRX: DRIVER,RAD50: DRIVER,SOS1: DRIVER,RXRA: DRIVER,VTCN1: DRIVER,AXIN1: DRIVER,NCOR1: DRIVER,BLM: DRIVER,CDK8: DRIVER,CCND2: DRIVER,IGF1R: DRIVER,ALOX12B: DRIVER,MTOR: DRIVER,CARM1: DRIVER,NTHL1: DRIVER,FANCD2: DRIVER,BARD1: DRIVER,ZRSR2: DRIVER,BABAM1: DRIVER,ERCC4: DRIVER,TCF7L2: DRIVER,PIK3C2G: DRIVER,POLQ: DRIVER,PDCD1LG2: DRIVER,NUP93: DRIVER,PARP2: DRIVER,CD28: DRIVER,TRIM33: DRIVER,EED: DRIVER,EXT2: DRIVER,AXIN2: DRIVER,RAD54L: DRIVER,FANCC: DRIVER,PIK3CB: DRIVER,LATS1: DRIVER,MAPK1: DRIVER,NCOA2: DRIVER,MIB1: DRIVER,HRAS: DRIVER,DTX1: DRIVER,U2AF1: DRIVER,EP300: DRIVER,CYLD: DRIVER,TLE4: DRIVER,TRAF2: DRIVER,MTAP: DRIVER,POLD1: DRIVER,NTRK2: DRIVER,SFRP4: DRIVER,RTEL1: DRIVER,LRP6: DRIVER,BRCA1: DRIVER,SLC45A3: DRIVER,FANCE: DRIVER,SPRTN: DRIVER,RPL22: DRIVER,INPP4A: DRIVER,FBXO31: DRIVER,GSK3B: DRIVER,NCOA1: DRIVER,FGFR1: DRIVER,AKT1: DRIVER,CDK4: DRIVER,ICOSLG: DRIVER,SDHA: DRIVER,ATM: DRIVER,MAX: DRIVER,DDX5: DRIVER,PPP1CB: DRIVER,NCOA3: DRIVER,ALK: DRIVER,ERCC5: DRIVER,SMAD4: DRIVER,PRKDC: DRIVER,TRAF3: DRIVER,PPP2R1A: DRIVER,TBL1XR1: DRIVER,SFRP2: DRIVER,PARP3: DRIVER,DKK4: DRIVER,CDK12: DRIVER,STK11: DRIVER,DDB2: DRIVER,NF2: DRIVER,NFE2L2: DRIVER,KMT2B: DRIVER,CPS1: DRIVER,TSC2: DRIVER,PTEN: DRIVER,CCND3: DRIVER,NUP214: DRIVER,FURIN: DRIVER,ERBB2: DRIVER,IDH2: DRIVER,AKT3: DRIVER,RET: DRIVER,TP53BP1: DRIVER,FUBP1: DRIVER,CUL3: DRIVER,RANBP2: DRIVER,EGFR: DRIVER,PRF1: DRIVER,RAD51: DRIVER,SOCS3: DRIVER,PIK3R2: DRIVER,GNAS: DRIVER,FGFR2: DRIVER,RHEB: DRIVER,WRN: DRIVER,WIF1: DRIVER,APC: DRIVER,FAT4: DRIVER,SLX4: DRIVER,VHL: DRIVER,NBN: DRIVER,CYP17A1: DRIVER,RPL10: DRIVER,CBLB: DRIVER,XPA: DRIVER,OMD: DRIVER,TRAF7: DRIVER,HLA-A: DRIVER,HLA-B: DRIVER,HSD3B1: DRIVER,CDKN1A: DRIVER,PDCD1: DRIVER,CD276: DRIVER,TGFBR1: DRIVER,PPP4R2: DRIVER,LATS2: DRIVER,RNF213: DRIVER,MED12: DRIVER,FAT1: DRIVER,PCBP1: DRIVER,KEAP1: DRIVER,UBR5: DRIVER,ARAF: DRIVER,PMS2: DRIVER,PRSS1: DRIVER,IDH1: DRIVER,RAD51D: DRIVER,GPC3: DRIVER,CRBN: DRIVER,MAD2L2: DRIVER,DDX41: DRIVER,KRAS: DRIVER,RELN: DRIVER,POLE: DRIVER,NCOR2: DRIVER,SF3B1: DRIVER,DKK1: DRIVER,ETNK1: DRIVER,KDSR: DRIVER,TNFAIP3: DRIVER,IL10: DRIVER,RAC1: DRIVER,CBL: DRIVER,CDKN1B: DRIVER,NT5C2: DRIVER,MAP3K1: DRIVER,DDX3X: DRIVER,TLE3: DRIVER,BAP1: DRIVER,PIK3C2B: DRIVER,NOTCH1: DRIVER,MSH2: DRIVER,YWHAE: DRIVER,NUP98: DRIVER,SPOP: DRIVER,QKI: DRIVER,ARNT: DRIVER,RBM10: DRIVER,KDM5A:

DRIVER,DCUN1D1: DRIVER,RNF43: DRIVER,PIK3CD: DRIVER,FUS: DRIVER,GNA11: DRIVER,ALB: DRIVER,ABL1: DRIVER,NTRK3: DRIVER,CTNNB1: DRIVER,PMS1: DRIVER,CDKN2A: DRIVER,SOCS1: DRIVER,RPL5: DRIVER,GPHN: DRIVER,PIK3C3: DRIVER,MAP2K2: DRIVER,MDM4: DRIVER,RAD52: DRIVER,MUTYH: DRIVER,ATR: DRIVER,CHEK1: DRIVER,CUL4A: DRIVER,ZNRF3: DRIVER,NF1: DRIVER,GNAQ: DRIVER,BRIP1: DRIVER,ZMYM3: DRIVER,PRKAR1A: DRIVER,DKK2: DRIVER,ATIC: DRIVER,TGFBR2: DRIVER,KDM2B: DRIVER,VEGFA: DRIVER,FLT3: DRIVER,TPR: DRIVER,WWTR1: DRIVER,NRAS: DRIVER,SDHC: DRIVER,RB1: DRIVER,MDM2: DRIVER,AKT2: DRIVER,RASA1: DRIVER,PRKD1: DRIVER,TLE2: DRIVER,PIK3CA: DRIVER,ACSL3: DRIVER,FANCA: DRIVER,HEY1: DRIVER,GMPS: DRIVER,MDC1: DRIVER,BIRC3: DRIVER,FGFR4: DRIVER,TRIM24: DRIVER,MGAM: DRIVER,SDC4: DRIVER,ACSL6: DRIVER,HLA-C: DRIVER,TLE1: DRIVER,NADK: DRIVER,CD274: DRIVER,BTLA: DRIVER,PC: DRIVER,FANCG: DRIVER,IL2: DRIVER,PPARG: DRIVER,KMT2A: DRIVER,NOTCH2: DRIVER,SFRP1: DRIVER,AMER1: DRIVER,SMAD2: DRIVER,INPP4B: DRIVER,SDHD: DRIVER,MGA: DRIVER,FANCL: DRIVER,CCND1: DRIVER
